# Intermittent convection-enhanced delivery of GDNF into rhesus monkey putamen: absence of local or cerebellar toxicity

**DOI:** 10.1007/s00204-018-2222-z

**Published:** 2018-05-22

**Authors:** Matthias Luz, Philip C. Allen, John Bringas, Chris Boiko, Diane E. Stockinger, Kristen J. Nikula, Owen Lewis, Max Woolley, H. Christian Fibiger, Krystof Bankiewicz, Erich Mohr

**Affiliations:** 1 MedGenesis Therapeutix Inc., Victoria, BC Canada; 2 Valley Biosystems Inc., West Sacramento, CA USA; 3 University of California, San Francisco, CA USA; 4 Seventh Wave Laboratories LLC, Maryland Heights, MO USA; 5 Neurological Applications Department, Renishaw plc, Wotton-under-Edge, Gloucestershire UK

**Keywords:** GDNF, Parkinson’s disease, Toxicology, Cerebellum, Purkinje cells, Putamen, Rhesus monkey, Convection-enhanced delivery

## Abstract

Glial cell line-derived neurotrophic factor (GDNF) has demonstrated neurorestorative and neuroprotective effects in rodent and nonhuman primate models of Parkinson’s disease. However, continuous intraputamenal infusion of GDNF (100 µg/day) resulted in multifocal cerebellar Purkinje cell loss in a 6-month toxicity study in rhesus monkeys. It was hypothesized that continuous leakage of GDNF into the cerebrospinal fluid compartment during the infusions led to down-regulation of GDNF receptors on Purkinje cells, and that subsequent acute withdrawal of GDNF then mediated the observed cerebellar lesions. Here we present the results of a 9-month toxicity study in which rhesus monkeys received intermittent intraputamenal infusions via convection-enhanced delivery. Animals were treated with GDNF (87.1 µg; *N* = 14) or vehicle (*N* = 6) once every 4 weeks for a total of 40 weeks (11 treatments). Four of the GDNF-treated animals were utilized in a satellite study assessing the impact of concomitant catheter repositioning prior to treatment. In the main study, eight animals (5 GDNF, 3 control) were euthanized at the end of the treatment period, along with the four satellite study animals, while the remaining eight animals (5 GDNF, 3 control) were euthanized at the end of a 12-week recovery period. There were no GDNF-related adverse effects and in particular, no GDNF-related microscopic findings in the brain, spinal cord, dorsal root ganglia, or trigeminal ganglia. Therefore, 87.1 µg/4 weeks is considered the no observed adverse effect level for GDNF in rhesus monkeys receiving intermittent, convection-enhanced delivery of GDNF for 9 months.

**Electronic supplementary material:**

The online version of this article (10.1007/s00204-018-2222-z) contains supplementary material, which is available to authorized users.

## Introduction

Glial cell line-derived neurotrophic factor (GDNF), originally isolated from a rat glioma cell line (Lin et al. 1993), is a neurotrophic factor with potent effects on dopaminergic, serotonergic, noradrenergic and cholinergic neurons (Airaksinen and Saarma 2002). GDNF levels in adult human brain are generally low; the highest concentrations are found in the caudate nucleus, putamen and substantia nigra (43–70 pg/mg protein), followed by cerebellum and frontal cortex (10–15 pg/mg protein) (Mogi et al. 2001). Upon binding to the membrane-anchored GDNF family ligand receptor (GFR)α-1 (at lower affinities also to GFRα-2 and GFRα-3), GDNF signals through the RET receptor tyrosine kinase (Airaksinen and Saarma 2002). Effective GDNF signaling additionally requires the presence of heparan sulfate glycosaminoglycans which serve as high abundance, low-affinity receptors on the cell surface and in the extracellular matrix (Barnett et al. 2002; Tanaka et al. 2002).

As a large molecule, GDNF cannot cross the blood–brain barrier and, therefore, has to be administered directly to its central nervous system (CNS) targets to achieve meaningful tissue levels (Allen et al. 2013). Following intraventricular, intrastriatal or intranigral delivery, GDNF has reproducibly demonstrated neurorestorative and neuroprotective effects in standard toxin-induced rodent and nonhuman primate models of Parkinson’s disease (PD) (Bjorklund et al. 1997; Gash et al. 1996; Grondin et al. 2002; Tomac et al. 1995; Zhang et al. 1997). Specifically, GDNF increased the number and perikaryal size of dopaminergic neurons in the substantia nigra, enhanced striatal dopamine metabolism, and increased tyrosine hydroxylase-positive fiber density in the striatum of nonhuman primates (Gash et al. 1996; Grondin et al. 2002). These phenotypic changes were associated with improved motor function (Allen et al. 2013). Based on these effects, GDNF has been considered a promising candidate for the disease-modifying treatment of PD.

The toxicology program undertaken to support the clinical development of recombinant-methionyl human GDNF (also referred to as GDNF in this article, unless differentiation from native GDNF is needed) included several studies in rhesus monkeys to examine the invasive drug delivery into the CNS. Altogether, 11 studies involving 205 animals were performed, including 6 studies testing the intracerebroventricular (ICV) route of administration (*N* = 86), 1 intrathecal (IT) study (*N* = 17) and 4 intraputamenal (IPu) studies (*N* = 102); the findings from these studies have recently been reviewed (Luz et al. 2016).

The primary GDNF toxicity of potential human relevance was cerebellar Purkinje cell loss which was observed in a chronic continuous IPu dosing study (Hovland et al. 2007). In that study, 72 rhesus monkeys were treated with vehicle or 15, 30 or 100 µg GDNF per day [GDNF concentration in the infusate (*C*_i_): 0, 0.1, 0.2, or 0.67 µg/µL] at an infusion rate of 6.25 µL/h for 6 months (6/sex/group), followed by a 3-month recovery period (3/sex/group). GDNF was generally well-tolerated based on the lack of adverse clinical signs, electrocardiogram alterations, neurological examination findings, changes in body weight, food consumption, and clinical laboratory evaluations at all doses. However, histopathologically, multifocal cerebellar Purkinje cell lesions affecting 1–21% of the cerebellar cortex were found in 4 of 15 (26.7%) high-dose animals. Lesion severity in the affected animals was generally minimal to moderate, but varied considerably by focus and by animal. Microscopic findings ranged from patchy Purkinje cell loss with proliferation of Bergman’s glia, astrocytosis and vacuolation in the molecular layer to more extensive lesions with translobar, nearly full thickness loss of Purkinje cells and granule cells, along with attenuation of the molecular layer (suggesting degeneration of Purkinje cell dendrites).

No cerebellar lesions were detected in any low- or medium-dose animals in the same study, or in any animal treated in one of three other subchronic or chronic toxicity studies [IT: one 3-month study (*N* = 17); ICV: one 3-month study with 1-month recovery (*N* = 24) and one 6-month study with 3-month recovery (*N* = 27)] (Hovland et al. 2007; Luz et al. 2016). Continuous dosing of GDNF in the culprit IPu study was found to be associated with significant dose-dependent GDNF leakage into the cerebrospinal fluid (CSF) compartment (Hovland et al. 2007). Based on a detailed review of the study data and research conducted since, it was hypothesized that extended leakage-induced exposure to high GDNF concentrations in CSF (*C*_CSF_) > 1700 pg/mL led to down-regulation of GDNF receptors on Purkinje cells, and that subsequent acute withdrawal of GDNF then resulted in the observed cerebellar lesions (Luz et al. 2016).

The present study was performed to determine the potential local and cerebellar toxicity of GDNF when given to rhesus monkeys by intermittent IPu convection-enhanced delivery (CED) for 40 weeks. CED is a method of delivering large-molecule drugs with a pressurized infusion system that induces directed bulk flow and leads to predictable, homogeneous drug distribution across large, clinically relevant volumes of tissue (Bobo et al. 1994; Fiandaca et al. 2008). The treatment protocol in the present study was designed to maximize the chances of reproducing the cerebellar findings observed in the continuous IPu dosing study, thus testing the hypothesis that intermittent dosing of GDNF would effectively eliminate the risk of cerebellar toxicity. This included using the same GDNF *C*_i_ as in the high-dose group of the continuous IPu dosing study (0.67 µg/µL), switching from unilateral to bilateral drug administration, and increasing the infusion volume per putamen to a level that was expected to be associated with limited leakage of infusate into the CSF compartment. In addition, the impact on the study outcome of added mechanical tissue damage from catheter repositioning prior to the start of treatment was assessed in a satellite study.

## Materials and methods

The study was performed as part of the nonclinical toxicology program supporting the clinical development of GDNF for the treatment of patients with PD. It was conducted in compliance with the United States Food and Drug Administration’s Good Laboratory Practice (GLP) for Nonclinical Laboratory Studies regulations as set forth in the Code of Federal Regulations (21 CFR Part 58) with the exception of an experimental software program that was used for the surgical planning of the catheter implantation. The treatment of animals was in accordance with the regulations of the United States Animal Welfare Act (9 CFR Parts 1, 2 and 3) and the conditions specified in the Guide for the Care and Use of Laboratory Animals (8th edition, National Academies Press, Washington, DC, USA, 2011). Moreover, all procedures were carried out under an Institutional Animal Care and Use Committee (IACUC) protocol of the testing facility (Valley Biosystems, West Sacramento, CA, USA), which in turn is accredited by the Association for the Assessment and Accreditation of Laboratory Animal Care International (AAALACi).

### Animals

Twenty-one purpose-bred, naïve Chinese rhesus monkeys (*Macaca mulatta*) were acquired from the testing facility’s animal colony. Animals were quarantined for at least 30 days and tested for tuberculosis, herpes B virus infection and simian retrovirus infection before study initiation. Only male animals were used in order to ensure suitability in size for the requisite surgical procedures; they were approximately 5–10 years old and weighed 7.16–12.85 kg at study entry. Animals were housed individually in stainless-steel cages. During the study, room temperature was maintained at 18–29 °C and relative humidity at 30–70%. Ventilation provided more than 10 air changes per hour, with 100% fresh air (no air recirculation). Lighting was automatically controlled to provide 12 h of light and 12 h of darkness. Standard environmental enrichment was provided to each animal. Due to the surgical procedures involved in this study and the age of the animals, socialization/comingling was not permitted. Teklad Certified Global 20% Protein Primate Diet #2050C (Harlan Laboratories, Indianapolis, IN, USA) was provided daily, supplemented with fruit or vegetables and Teklad Irradiated Enrich Mix (Harlan Laboratories). Animals were fasted overnight before body weight measurements, surgeries, dosing procedures, and blood sampling for clinical chemistry. Municipality tap water (Sacramento, CA, USA) was provided ad libitum and not withheld at any time during the study.

### Overall study design

**Fig. 1:**
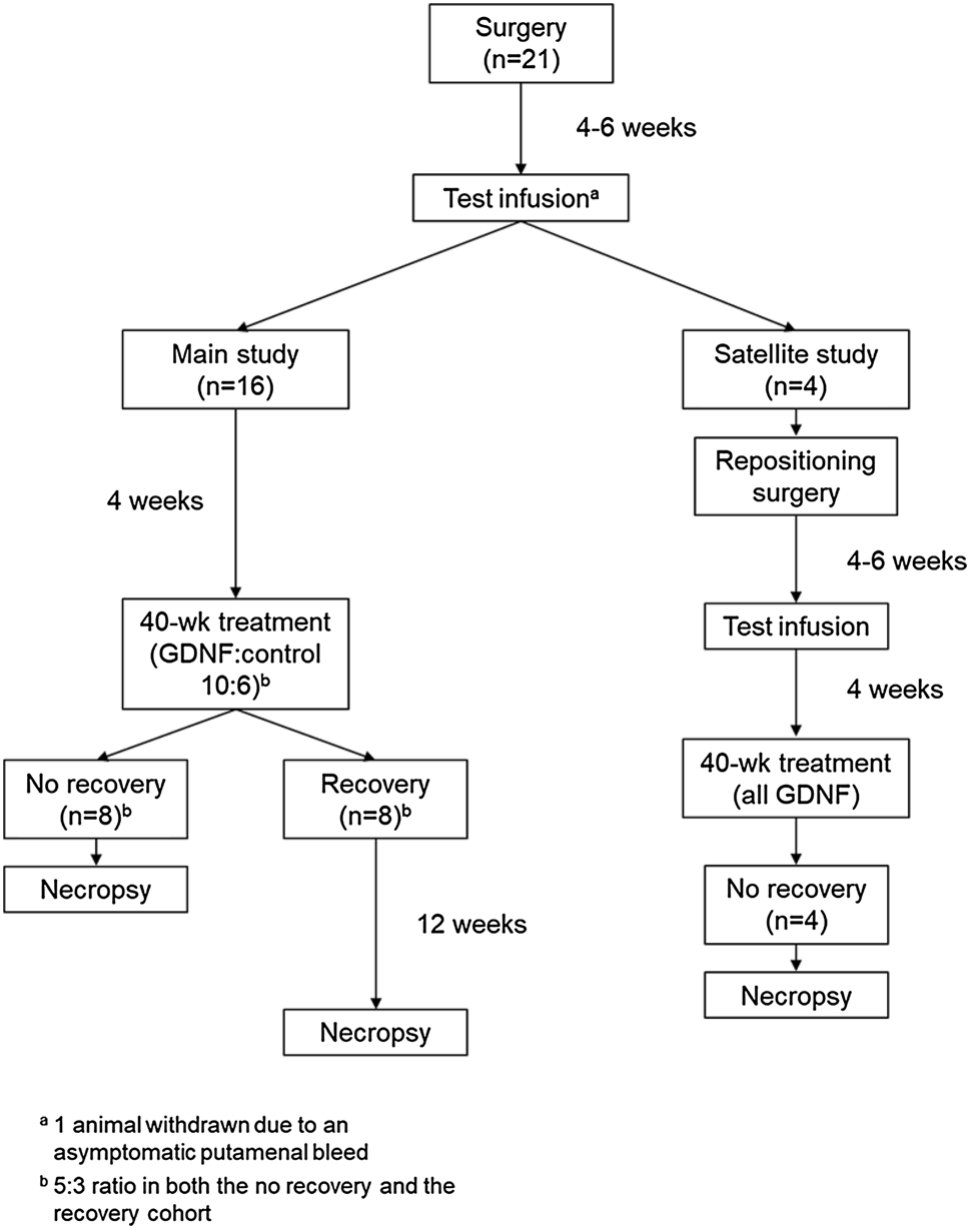
Overall study design

At study start, all 21 animals were surgically implanted within 1 week with a customized drug-delivery system (DDS; Renishaw, Wotton-under-Edge, UK). Pre-operative determination of stereotactic coordinates and trajectories for the positioning of the CED catheters, and post-operative confirmation of proper catheter placement was performed via magnetic resonance imaging (MRI). Following a 4- to 6-week healing period, animals were MRI-scanned again and then received a test infusion of contrast-enhanced control article. Post-test infusion, an additional MRI scan was performed to verify catheter performance. One animal was withdrawn at this point because of an asymptomatic putamenal bleed, leaving 20 animals on study. The 16 animals with the best performing catheters were allocated to the main study; the remaining 4 animals were allocated to the satellite study. Main study animals were randomized 5:3 to GDNF or control by a stratified scheme designed to achieve similar mean body weights in both groups. All satellite study animals were assigned to GDNF; they underwent surgical CED catheter repositioning on both sides (with pre- and post-operative MRI), followed by another 4- to 6-week healing period and a subsequent repeat test infusion (with pre- and post-infusion MRI) before the start of treatment. Approximately 4 weeks after the definitive test infusion, dosing via IPu CED was initiated and continued for 40 weeks (11 doses). A final set of MRI scans were performed before and after the final dose. At the end of the treatment period, main study animals were randomly assigned to no-recovery or recovery (8 animals each, 5:3 ratio). All satellite study animals were assigned to no-recovery. No-recovery animals were necropsied within 1–4 days of final dose, recovery animals at the end of the 12-week recovery period. A schematic of the overall study design is provided in Fig. 1. Detailed methods for select procedures are provided in the following sections.

### Drug-delivery system implantation and catheter repositioning

#### Drug-delivery system

**Fig. 2:**
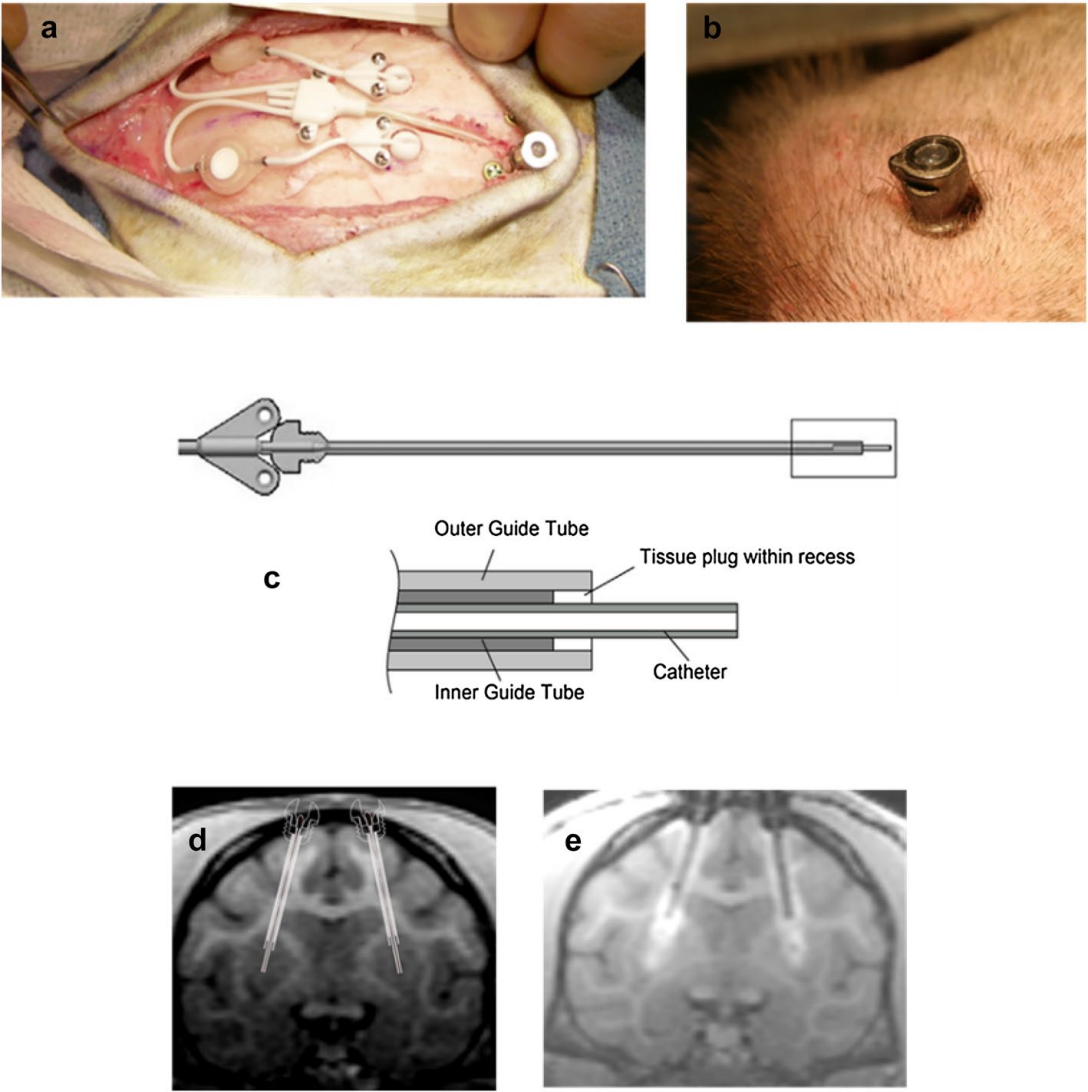
Drug-delivery system (DDS). **a** Surgical site with implanted DDS including transcutaneous, bone-anchored port, in-line particle filters, and winged microcatheters attached to the domed guide tube hubs. **b** Transcutaneous port at study end (11 months post-implantation). **c** Recessed step catheter. **d** Surgical plan showing targeted recessed step catheter positions. **e** T1-weighted coronal MRI scan after test infusion of 2 mM gadopentetate dimeglumine

The implantable DDS included three major elements—a single transcutaneous, skull-anchored, multichannel port; two low-volume, in-line particle filters; and two recessed step catheters (Fig. 2a). The port was essentially designed as previously published (Barua et al. 2013), with minor adjustments for optimal fit in the rhesus monkey model (Fig. 2b). The recessed step catheters were designed to minimize reflux and comprised an inner and an outer guide tube and an indwelling catheter with an adjustable winged stop (Fig. 2 c) (Gill et al. 2013). For each infusion, an external two-line administration set with in-line air and particle filters was attached to the port via a kinematically mounted needle connector.

#### Anesthesia and post-operative management

Prior to the surgical procedures, the animals were sedated with an intramuscular (im) injection of ketamine (10 mg/kg; Vedco, Saint Joseph, MO, USA) and medetomidine (0.015 mg/kg; Zoetis, Kalamazoo, MI, USA). Tracheal intubation was performed and the animals were placed on inhaled isoflurane (1–4.5%; Piramal Healthcare, Nashville, TN, USA) in oxygen (delivered at 1.0 L/min). Homeostatic monitoring (respirations, vital signs) was performed according to locally established procedures. After completion of the surgical intervention, atipamezole hydrochloride (im, 0.15 mg/kg; Zoetis) was administered and the animals were extubated and returned to their home cages. The animals were visually monitored cage side at 15-min intervals until full recovery from anesthesia. For analgesia and post-operative care, animals received, at minimum, buprenorphine (im, 0.03 mg/kg twice daily for 1.5 days; Patterson Veterinary, Mendota Heights, MN, USA), carprofen (subcutaneously or orally, 2.2 mg/kg twice daily for 2 days; Zoetis), and ceftriaxone (im, 50 mg/kg once daily for 5 days; Patterson Veterinary). Following completion of the carprofen, all animals were given ketofen (im, 2 mg/kg once daily for 3 days; Zoetis).

#### DDS implantation

The surgical procedure was essentially based on a method previously developed for implantation of a similar device in Large White/Landrace pigs (Barua et al. 2013; Gill et al. 2013). The animals were placed in a customized MRI-compatible head frame and fiducial system for nonhuman primates (Renishaw). To maximize the accuracy of catheter placement, the animals remained in the frame during transportation between the surgical room and the MRI unit, where a high-resolution planning scan was acquired. MRI DICOM series were imported into an investigational preclinical drug-delivery module of the neuro|inspire™ software (Renishaw) which was used for surgical planning and to identify the targeted regions of the putamen (anterior putamen in 11 animals, posterior putamen in 9 animals, with the catheter tips being placed in the ventral putamen, and the outer and inner guide tube junctions in the dorsal putamen). Two-dimensional representations of the catheters were placed over the desired implantation trajectories with the guide tube tips positioned approximately 3 mm into the dorsal aspect of the putamen (Fig. 2d). The implantation site of the port was selected based on areas of large bone thickness lateral to the sagittal suture over the frontal brow, avoiding the orbital cavity. A human stereotactic frame (Radionics^®^ CRW™, Integra LifeSciences, Plainsboro, NJ, USA) was mounted to the fixation frame prior to surgery. Two small holes (one per hemisphere) were stereotactically drilled over transfrontal regions of the skull using aseptic techniques. The outer guide tube was stereotactically aligned to the desired trajectory and introduced through the burr hole into the dorsal aspect of the putamen. The threaded hub of the tube was press-fitted into the skull prior to introducing the inner guide tube. Two microcatheters were aseptically connected to separate in-line particle filters and flexible tubing that terminated at the port. An external administration set was attached and the entire system was primed with artificial cerebrospinal fluid (aCSF; MedGenesis Therapeutix, Victoria, BC, Canada). The microcatheters were cut to the required length and then manually introduced through the inner guide tube into the ventral putamen while aCSF was infused at 0.5 µL/min. The implanted system was secured to the skull using light-cured acrylic (Triad Gel, Dentsply Inc., York, PA, USA) and 1.6-mm diameter titanium bone screws. The stereotactic frame was then re-adjusted to create a cavity for the port. A manual burr and template were used to create a recess for the tubing and the cross form of the port base. The port was tapped into place and secured with bone plates (Fig. 2a). Collected bone swarf was packed back into the tubing trench. The surgical site was closed in anatomical layers and a punch biopsy incision was made over the bone-anchored port to create a tight-fitting hole in the skin for the transcutaneous port (Fig. 2b). A post-surgery MRI was acquired for verification of catheter placement. The animals were allowed to recover for 4–6 weeks before performing the test infusions.

#### Catheter repositioning

Following the test infusions, the four animals assigned to the satellite group underwent a second implantation procedure to position two additional catheters within the putamen and an additional access port on the skull. Several components of the initial implant were removed (the microcatheters, in-line filters, and subcutaneous tubing), whereas the guide tubes and transcutaneous access port were left in situ. A small stylet was placed in each of the unused guide tubes to seal the system. The second DDS was implanted following the procedures described above. Anterior and posterior targets were reversed from the initial implantation. The implanted hardware (2 catheters per hemisphere and 2 ports) remained in situ until the end of the study. The animals were allowed to recover for 4–6 weeks before performing the retest infusions.

### MRI scans

MRI scans were acquired in all animals pre- and post-implantation surgery, before and within 1 h after the test infusion, and before and within 1 h after the final dose. Additional scans were acquired in satellite study animals pre- and post-catheter repositioning surgery, and before and within 1 h after the retest infusion. Anesthesia procedures were the same as described under DDS implantation and infusion procedures, respectively. Scanning procedures were performed on a 1.5 T mobile scanning unit (Signa HDxt, GE Healthcare, provided by DMS Imaging/DMS Health Technologies, Fargo, ND, USA) using two 3-in. dual channel temporomandibular joint (TMJ) surface coils. Three imaging sequences [three-dimensional T1 fast spoiled gradient-echo (FSPGR), coronal T2 fast spin echo (FSE), and coronal T2 fluid attenuation inversion recovery (FLAIR)] were acquired at each imaging event. The scanning parameters were as follows: T1 FSPGR—*T*_R_ = 8.5 ms, *T*_E_ = 3.35 ms, FA = 15°, slice thickness = 0.8 mm, slice spacing = 0.4 mm; T2 FSE—*T*_R_ = > 4500, *T*_E_ = > 92, FA = 90, slice thickness = 1 mm, slice spacing = 1 mm; and T2 FLAIR—*T*_R_ = 9500, *T*_E_ = 125–150, FA = 90, TI = 2250–2375, slice thickness = 1 mm, slice spacing = 1 mm. The total scan time was approximately 10–15 min. T1-weighted post-(re)test infusion scans were used to determine the parenchymal contrast distribution (Fig. 2e). On all scans, neuroanatomical structures in geographic proximity to the catheter track and infusion site were assessed for signs of hemorrhage, edema, cysts or other findings related to the infusion system. Particular emphasis was placed on the evaluation of the striatum, globus pallidus, substantia nigra, subthalamic nucleus, thalamus, cortex and cerebellum.

### Infusion procedures and dosing scheme

#### Anesthesia and post-infusion management

On infusion days, animals were sedated with an im injection of ketamine (10 mg/kg; Vedco) and medetomidine (0.015 mg/kg; Zoetis) before the start of the procedures. For (re)test and final study infusions (where animals underwent pre- and post-infusion MRI scans), subsequent anesthesia procedures were the same as those performed on surgery days. On all other infusion days, animals only received atipamezole hydrochloride (im, 0.15 mg/kg; Zoetis) after completion of the infusion and were then returned to their home cages, where they were visually monitored cage side at 15-min intervals until full recovery from anesthesia.

#### Infusions

The infusion procedures were essentially the same for test and study infusions. Prior to the infusions, two 65-µL sets of fixed-volume extension tubing were filled with 2 mM gadopentetate dimeglumine (Bayer Healthcare Pharmaceuticals Inc., Wayne, NJ, USA) in aCSF for test infusions, and with GDNF (*C*_i_: 0.67 µg/µL in aCSF, total brain dose: 87.1 µg; MedGenesis) or aCSF for therapeutic infusions in accordance with the treatment allocations. The animal was placed in the prone position, the head was shaved, and the port was prepared for aseptic connection to an administration set. Two 5-mL syringes were filled with aCSF, connected with a single administration set via additional extension tubing, and mounted onto infusion pumps (Perfusor^®^ Space, B Braun Medical, Sheffield, UK). Upon priming of the entire system with aCSF, the infusion lines were disconnected again, and the prefilled fixed-volume extension sets were placed between the additional extension tubing and the administration set and connected at both ends. The pumps were set to deliver a total of 37.5 µL per line, which ensured that 5 µL of aCSF were left in each line before the treatment fraction reached the tip of the corresponding needle. Within approximately 10 s of completing the priming process, the administration set was attached to the port, and the study infusion protocol was started. Infusions were ramped up over 40 min from 0 to 3 µL/min, followed by a 22-min maintenance phase at a constant rate of 3 µL/min, and a final ramp-down over 2 min from 3 to 0 µL/min. The total volume delivered per catheter (*V*_i_) was approximately 130 µL, including a 60-µL fraction of aCSF at the end to clear the catheter dead space at the end of the procedure. Therefore, the target tissue was unavoidably perfused with 65 µL of aCSF before arrival of the treatment fraction. Moreover, since the maximum IPu infusion volume in rhesus monkey without inducing reflux is in the range of 50–70 µL (Varenika et al. 2008), the chosen volume of 130 µL per catheter was likely to induce reflux into the CSF compartment. It was contemplated that reflux would be mostly limited to the first 65 µL of infusate (i.e., aCSF), so that most of the treatment fraction would remain within the putamen. Ten minutes after the end of the infusion, the administration set was removed. Following post-(re)test infusion MRI scans, the distribution volume (*V*_d_) was determined by means of OsiriX (Pixmeo SARL, Geneva, Switzerland), an open source DICOM reader and imaging workstation. The *V*_d_/*V*_i_ ratio and putamenal volume coverage were then computed for each hemisphere.

### In-life observations

Animals underwent general health and moribundity/mortality observation (twice daily), cage side observation (daily) and evaluation of food consumption (observed daily, summarized weekly), body weight (weekly) and neurological changes (behavior, menace, pupil size, strabismus, facial symmetry, vestibular head tilt, nystagmus [resting, positional, vestibular], gait and posture; every 3 months). Clinical pathology (hematology, serum chemistry, coagulation, urinalysis) was evaluated pre-study, prior to final dose, and pre-necropsy (recovery animals). GDNF *C*_CSF_ was determined pre-study, before and within 1 h after both 2nd (day 29) and final (day 281) dose, and pre-necropsy (recovery animals). Binding and neutralizing antibodies against GDNF were determined pre-study, before 2nd, 4th and final dose, and pre-necropsy (recovery animals) in serum, and pre-study, before and within 1 h after both 2nd and final dose, and pre-necropsy (recovery animals) in CSF.

GDNF *C*_CSF_ was determined using a validated enzyme-linked immunosorbent assay (ELISA). Microwell plates were coated with a monoclonal mouse anti-r-metHuGDNF antibody (MAB212; R&D Systems, Abingdon, UK) as the capture antibody. After three washing steps, diluted samples were transferred to the assay plate. Unbound GDNF was removed by washing the wells and a biotinylated polyclonal goat anti-human GDNF antibody (BAF212; R&D Systems) was added for detection of the captured GDNF. After a washing step, streptavidin HRP conjugate (DY998; R&D Systems) was added, followed by another washing step and the addition of TMB substrate (T0440; Sigma-Aldrich, Gillingham, UK), before 1 M H_2_SO_4_ was added to stop the reaction. The plate was then read at 450 nm, with background correction of 620 nm. The lower limit of quantification was 39.06 pg/mL.

Anti-GDNF binding antibodies were determined using a validated surface plasmon resonance (SPR) assay on a Biacore 3000 (GE Healthcare Life Sciences, Little Chalfont, UK), a biosensor-based instrument that monitors biomolecular-binding events. GDNF-coupled Biacore CM5 sensor chips were prepared by diluting GDNF in sodium acetate pH 4.5 and immobilizing to the chip using standard amine chemistry, aiming for a response target of 2000 resonance units. Samples were diluted 1 in 10 in HBS-EP running buffer (BR-1001-88; GE Healthcare) plus 0.5% carboxymethyl dextran and 150 mM NaCl (serum assay only), and filtered using a 0.22 µm pore size hydrophilic PVDF membrane. The samples were injected into the Biacore and detection was based on measured changes in the refractive index due to accumulation of mass (GDNF antibodies) on the coupled chip surface vs. an uncoupled chip surface. The chip surface was regenerated between cycles with two injections of 50 mM HCl 5% P20.

Anti-GDNF neutralizing antibodies were determined using a validated cell-based bioassay. The bioassay used 32D REG cl11 cells, a custom engineered cell line that is a clone of murine 32Dcl3 cells transfected with the GDNF receptor alpha unit, as well as a chimeric co-receptor. These cells proliferate in the presence of GDNF or murine interleukin-3 (mIL-3). A sample that inhibited GDNF-induced proliferation but not mIL-3-induced proliferation was considered positive for neutralizing antibodies. Detection was via a luminescence light measurement of the cultured cells’ ATP levels using the ViaLight™ plus kit (LT07-121; Lonza, Walkersville, MD, USA).

### Post-mortem procedures

All animals were subjected to a full necropsy under the supervision of a veterinary pathologist. Necropsies occurred at the end of the treatment period and at the end of the recovery period. Where possible, the animals were euthanized rotating across dose groups. Animals were euthanized with 100 mg/kg sodium pentobarbital (Virbac, Fort Worth, TX, USA), and terminal body weight was recorded. Whole-body perfusion was performed through the left cardiac ventricle or the aorta with cold sterile physiologic saline containing 2 IU/mL of heparin (Patterson Veterinary) at 150 mL/min for 5–10 min, followed by 4% paraformaldehyde (American MasterTech Scientific, Lodi, CA, USA) at 150 mL/min for 15 min.

All major organs were weighed, and samples of relevant tissues were collected from each animal. Samples from outside the CNS were preserved in 10% neutral buffered formalin (Medical Chemical, Torrance, CA, USA) or 4% paraformaldehyde (American MasterTech Scientific). In view of the large database available from previous toxicology studies (Luz et al. 2016), these samples were archived but not analyzed. CNS samples (22 coronal brain slices including 3 cerebellar slices; cervical, thoracic and lumbar spinal cord; cervical, thoracic and lumbar dorsal root ganglia; and trigeminal ganglia) were initially preserved in 4% paraformaldehyde and then transferred to 10% neutral buffered formalin within 4 days. These samples were trimmed and embedded in paraffin (brain and spinal cord) or glycol methacrylate (ganglia). At least 1 section from each block was mounted on slides and stained with hematoxylin and eosin (H&E). Another four slides including putamen, substantia nigra, ventral tegmental area and subthalamic nuclei, and two slides of cerebellum per animal were prepared for staining with Luxol fast blue-periodic acid Schiff (LFB-PAS), Fluoro-Jade C (FJC), anti-glial fibrillary acidic protein (GFAP), and Bielschowsky’s silver stain (one set of slides per stain). In addition, 19 slides of brain, 2 slides of cerebellum, and 3 slides of spinal cord (cervical, thoracic, lumbar) from each animal were stained immunohistochemically with anti-human GDNF antibody (BAF212; R&D Systems) to detect GDNF protein. Selected pathology data including microscopic data and the draft and final pathology report for the study were peer-reviewed.

### Statistical methods

Continuous parameters such as body weight or clinical pathology measurements were analyzed using a mixed model analysis of variance. If the residual errors from the analysis were markedly non-normal (Wilk Shapiro test statistic < 0.95), the data were log-transformed and the analysis repeated. If the residual errors were still markedly non-normal, then a non-parametric analysis (Kruskal–Wallis test) was used. For the analysis of body weight data, an autoregressive [AR(1)] error structure was used to model dependence over time. For all other continuous responses, a variance component structure was assumed. For parameters expressed as counts of behaviors, the initial analysis approach was to run a mixed Poisson model. Dichotomous parameters such as low food intake on a given day were initially analyzed using a binomial generalized linear model; if this was computationally infeasible because of low numbers, the data were summarized over weeks and then analyzed non-parametrically. All of these analyses used factorial models to divide the systematic differences in the data into a main effect of the treatment, which averaged the treatment effect across sampling events, and a treatment × week interaction, which tested whether the form of the temporal changes was similar between GDNF-treated animals and control animals. In addition, analyses were conducted that focused on differences between the treated groups in the main and satellite study. The significance of the main effect of satellite (i.e., the difference between the satellite study and the GDNF group of the main study, averaged across sampling events) and the satellite × week interaction were analyzed.

## Results

### In-life results

All animals were surgically implanted with the DDS including 2 CED catheters (1 per putamen) and a transcutaneous port (Fig. 2a). The four satellite animals underwent repeat surgery for catheter repositioning 4–6 weeks after the initial surgery. The procedures were generally well-tolerated and surgical recovery was mostly uneventful. One animal developed a unilateral post-surgical cerebral edema within the dorsal putamen and around the catheter track that resolved within a few days of treatment with dexamethasone. Quantitative MRI evaluation was performed on all animals to measure the targeting accuracy (post-surgery) and performance characteristics (post-test infusion 4–6 weeks after surgery) of the CED catheters (Fig. 2e). Consistent with the size differences between animals, depth to target varied from 25.4 to 34.2 mm (mean 28.5 ± 1.8 mm). Targeting accuracy was high in all animals, with target errors < 1.5 mm in 47 of 48 cases (including repeat implantations in the satellite study) and 2.4 mm on one side after the initial surgery in a satellite animal (mean: 0.9 ± 0.4 mm). Post-test infusion infusate distribution (mean *V*_d_/*V*_i_: 2.4 ± 0.3) and putamenal coverage (mean: 25.7 ± 6.6%) in the main study were consistent with expectations considering the anticipated reflux. Values after the repeat test infusion in the satellite study (mean *V*_d_/*V*_i_: 2.3 ± 0.3, mean putamenal coverage: 24.0 ± 4.7%) were similar, confirming the feasibility and usefulness of repeat implantation surgery in case of unsatisfactory distribution results.

All animals received all study infusions and completed the study as planned, with the exception of 1 control animal that removed the CED catheter on one side after the 7th infusion and received 4 unilateral infusions thereafter. Hence, 216 (98.2%) of 220 infusions were delivered bilaterally as planned, the remaining 4 (1.8%) unilaterally. There were only minimal cage side findings, mostly related to surgery. Food consumption was generally good, with no relevant or statistically significant differences between treatment groups and satellite study animals. Mean body weight increased similarly in both treatment groups of the main study (GDNF: 8.93 ± 0.87 to 10.17 ± 1.05 kg; control: 9.28 ± 1.38 to 10.96 ± 1.80 kg) and in satellite study animals (10.87 ± 2.54 to 11.40 ± 2.41 kg) during the treatment period. During the recovery period, there were only small changes in body weight. No abnormal findings were made at any of the neurological examinations in the study, and there were no noticeable GDNF-related effects on any clinical pathology parameters.

Sampling for the determination of GDNF *C*_CSF_ and antibodies against GDNF in CSF and serum was performed according to schedule, and all samples (CSF: 108, serum: 88) were analyzed as planned. Quantifiable GDNF *C*_CSF_ values ranging from 98.32 to 73,264.78 pg/mL were found in post-dose samples of all 14 GDNF-treated animals after both 2nd (day 29) and final (day 281) dose (Table 1). A total of 9 (64.3%) animals had values > 1700 pg/mL at least once in the study, and 5 (35.7%) animals at both time points; two of the latter animals were in the recovery cohort. The values in all other samples from both treatment groups were below the lower limit of quantification (Table 1).

**Table 1:** GDNF concentrations in cerebrospinal fluid

Group	Animal	GDNF concentration in CSF (pg/mL)				
			2nd dose (day 29)		Final dose (day 281)	
		Pre-study	Pre-dose	Post-dose	Pre-dose	Post-dose
No-recovery	V001946	< LLoQ	< LLoQ	4669.81	< LLoQ	< LLoQ
	V001954	< LLoQ	< LLoQ	1199.11	< LLoQ	468.98
	V002593	< LLoQ	< LLoQ	3392.48	< LLoQ	7639.83
	V002611	< LLoQ	< LLoQ	< LLoQ	< LLoQ	929.98
	V002615	< LLoQ	< LLoQ	149.65	< LLoQ	797.17
Recovery	V001935	< LLoQ	< LLoQ	262.82	< LLoQ	157.78
	V002047	< LLoQ	< LLoQ	4422.77	< LLoQ	38,466.04
	V002608	< LLoQ	< LLoQ	8873.61	< LLoQ	305.64
	V002610	< LLoQ	< LLoQ	2608.37	< LLoQ	2149.44
	V002614	< LLoQ	< LLoQ	2421.03	< LLoQ	895.04
Satellite	V001633	< LLoQ	< LLoQ	2385.66	< LLoQ	73,264.78
	V001963	< LLoQ	< LLoQ	98.32	< LLoQ	554.80
	V002043	< LLoQ	< LLoQ	2489.32	< LLoQ	2948.85
	V002603	< LLoQ	< LLoQ	549.03	< LLoQ	2310.39
All	Median	0	0	2421.03	0	929.98
	Mean	0	0	2578.61	0	10,068.36
	SD	–	–	2442.18	–	21,620.78

*LLoQ* lower limit of quantification

Anti-GDNF binding antibodies in serum were detected in 8 (57.1%) of the 14 GDNF-treated animals (including 2 satellite study animals) and 1 (16.7%) of the 6 control animals (one sample only; isolated positive samples in control animals were also found by Hovland et al. 2007). Five (35.7%) of the GDNF-treated animals were also found to have anti-GDNF neutralizing antibodies in serum, and one (7.1%) in addition had positive findings for anti-GDNF binding and neutralizing antibodies in CSF.

MRI evaluations of the globus pallidus, substantia nigra, subthalamic nucleus, thalamus, and cerebellum appeared normal in all animals and did not generate any significant findings at any time. Most of the MRI findings were made in the striatum and cortex and were most likely related to the implanted infusion system. There was no indication of any GDNF-related effects in the MRI findings. As expected with intracerebral device implantation, MRI scans frequently showed minor asymptomatic hemorrhage in the tissues around the catheter track following surgery [9 (45%) of 20 animals]. Infusate reflux along the catheter track was also seen frequently [12 (60%) of 20 animals]. This was predicted due to the large infusion volumes delivered. There was no indication that the presence of a second catheter track in the same hemisphere increased reflux in the satellite study animals.

### Post-mortem results

There were no GDNF-related effects on gross necropsy findings or organ weights. The mean ratio of brain weight to terminal body weight was similar in the animals of both treatment groups in the main study (GDNF: 1.03 ± 0.13%; control: 0.95 ± 0.14%) and in the satellite study animals (1.03 ± 0.26%).

As expected, immunohistochemical staining to detect GDNF showed GDNF immunostaining across multiple sections in both hemispheres of all GDNF-treated animals, including recovery animals where the staining intensity was reduced from moderate to mild relative to the animals necropsied at the end of the treatment period (Table 2). The staining was mostly seen in the catheter tracks and/or the neuropil adjacent to the catheter tracks. No GDNF immunostaining was observed in any of the control animals. These results confirm that GDNF was properly delivered in both hemispheres of all GDNF-treated animals.

**Table 2:** Incidence and severity of main microscopic findings in the catheter tracks and/or the adjacent neuropil

	GDNF			Control	
	Main study		Satellite	Main study	
	No-recovery	Recovery		No-recovery	Recovery
	*N* = 5	*N* = 5	*N* = 4	*N* = 3	*N* = 3
GDNF immunostaining	5	5	4	0	0
Minimal	–	–	–	–	–
Mild	–	5	–	–	–
Moderate	4	–	3	–	–
Marked	1	–	1	–	–
Fibrosis	5	5	4	3	3
Minimal	–	–	2	–	1
Mild	3	3	2	2	1
Moderate	2	2	–	1	1
Mineralized material	5	4	4	2	3
Minimal	2	4	4	1	3
Mild	3	–	–	1	–
Foreign body reaction	5	5	4	3	3
Minimal	4	4	3	2	3
Mild	1	1	1	1	–
Hemorrhage	1	0	1	1	0
Minimal	1	–	1	1	–
Pigmented macrophages	5	5	4	3	3
Minimal	1	3	3	3	3
Mild	3	2	1	–	–
Moderate	1	–	–	–	–
Vacuolated macrophages	2	1	1	1	1
Minimal	1	–	–	–	1
Mild	1	1	1	1	–
Infiltration, mononuclear cells	5	4	3	3	3
Minimal	4	3	3	2	2
Mild	1	1	–	1	1
Infiltration, neutrophil	0	1	0	1	0
Minimal	–	–	–	–	–
Mild	–	–	–	–	–
Moderate	–	1	–	1	–
Infiltration, eosinophil	5	3	1	2	2
Minimal	4	2	1	2	2
Mild	1	1	–	–	–
Perivascular cuffs, mononuclear cell	0	2	0	0	1
Minimal	–	1	–	–	1
Perivascular cuffs, mixed-cell	3	1	1	2	0
Minimal	2	1	1	1	–
Mild	1	–	–	1	–
Gliosis/astrocytosis	4	5	3	3	3
Minimal	3	2	2	2	3
Mild	–	3	1	1	–
Moderate	1	–	–	–	–
Vacuoloation, white matter	3	3	3	3	2
Minimal	3	1	2	2	2
Mild	–	1	1	1	–
Moderate	–	1	–	–	–
Vacuolation, gray matter	0	2	1	1	0
Minimal	–	2	1	–	–
Mild	–	–	–	1	–
Axon spheroids	0	1	0	1	0
Minimal	–	1	–	1	–

**Fig. 3:**
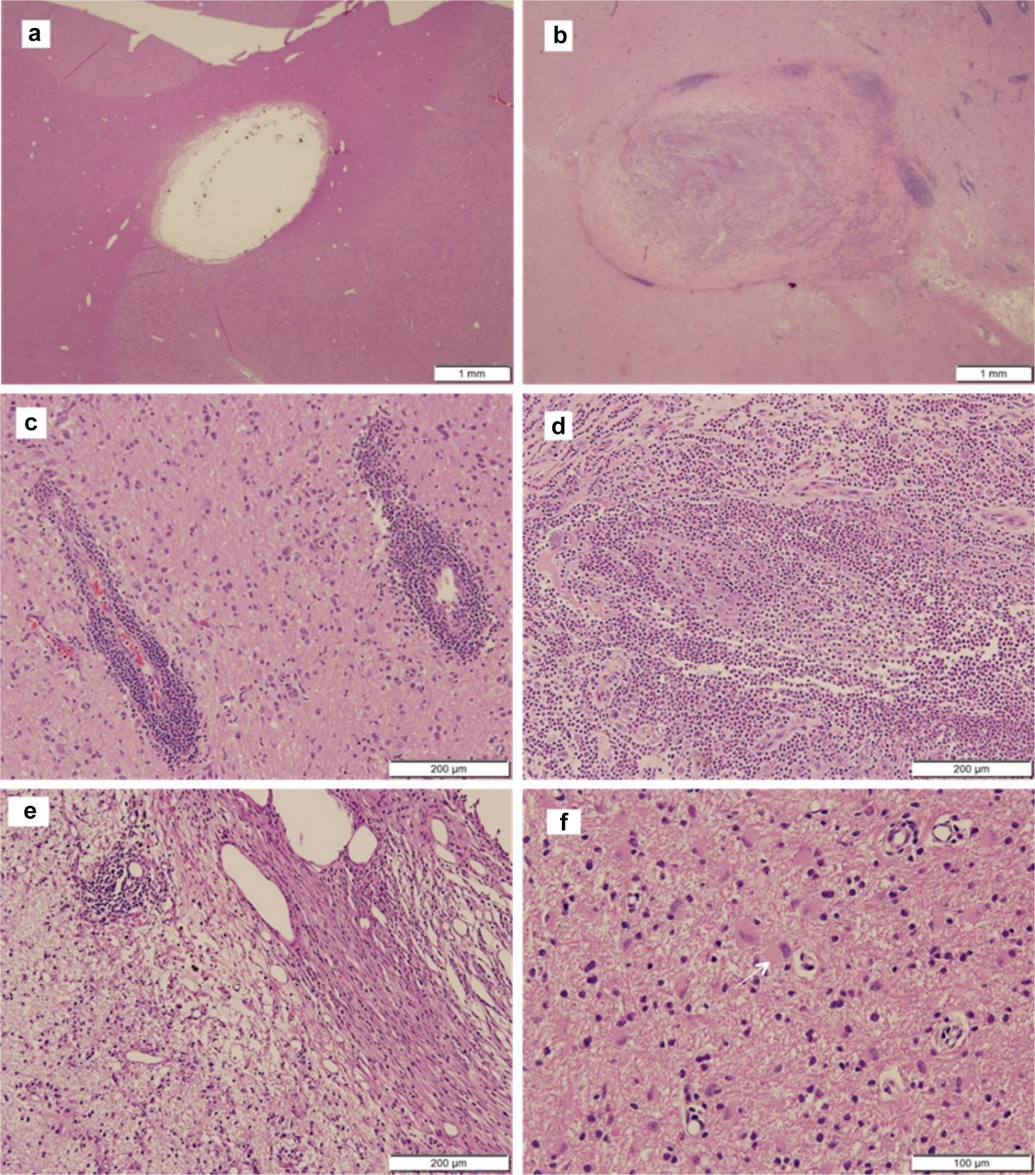
Microscopic findings in the catheter tracks and/or the adjacent neuropil. **a** Minimal to mild fibrosis, inflammation and tissue reaction was observed in most animals of both treatment groups (the picture shows a control animal in the no-recovery cohort). **b** Non-GDNF-related adverse catheter track reactions were only seen in two animals, a control animal in the no-recovery cohort (shown) and a GDNF-treated animal in the recovery cohort. Findings in these animals included perivascular mononuclear cell infiltrates (**c**; control), moderate neutrophilic inflammation (**d**; control), edema formation (**e**; GDNF) and reactive gliosis/astrocytosis (arrow in **f**; GDNF). Staining: H&E

Table 2 shows the incidence and severity of the main microscopic findings in the study. They were confined to the catheter tracks and adjacent neuropil, and were generally consistent with expected tissue reactions in response to an implanted device of this type (Fig. 3a) (Butt 2011; Polikov et al. 2005). The findings exhibited the same characteristics and similar incidence and severity across the treatment groups. Based on their limited distribution and severity, microscopic findings were generally not considered adverse. There were two exceptions, one in a control animal in the no-recovery cohort and one in a GDNF-treated animal in the recovery cohort (Fig. 3b–f). Both animals showed moderate neutrophilic inflammation (infiltration, neutrophil) and an associated slightly increased tissue reaction around one catheter track (control animal: combination of mixed-cell perivascular cuffs, gliosis/astrocytosis, and gray matter vacuolation; GDNF-treated animal: white matter vacuolation and gliosis/astrogliosis). In both cases, the findings were considered adverse due to their severity and character, which was highly suggestive of a response to a local bacterial infection. The animals had a common clinical history of protracted local port site infection that was presumably tracking along the subcutaneous tubing and intracerebral catheters on one side.

One GDNF-treated animal in the no-recovery cohort with a history of post-surgical swelling and intermittent fluid accumulation caudal to the incision site, exhibited a moderate accumulation of pigmented macrophages and a moderate tissue reaction (gliosis/astrogliosis) around one catheter track (Table 2). The pigment was consistent with hemosiderin as an indicator of prior hemorrhage. The findings were not of high enough severity to be considered adverse, particularly given the limited distribution and absence of any associated degenerative findings. GDNF was not thought to play a role in the development of the findings in this animal given that their characteristics were consistent with anticipated catheter-related effects as demonstrated by comparable findings noted in many other animals, including controls.

**Fig. 4:**
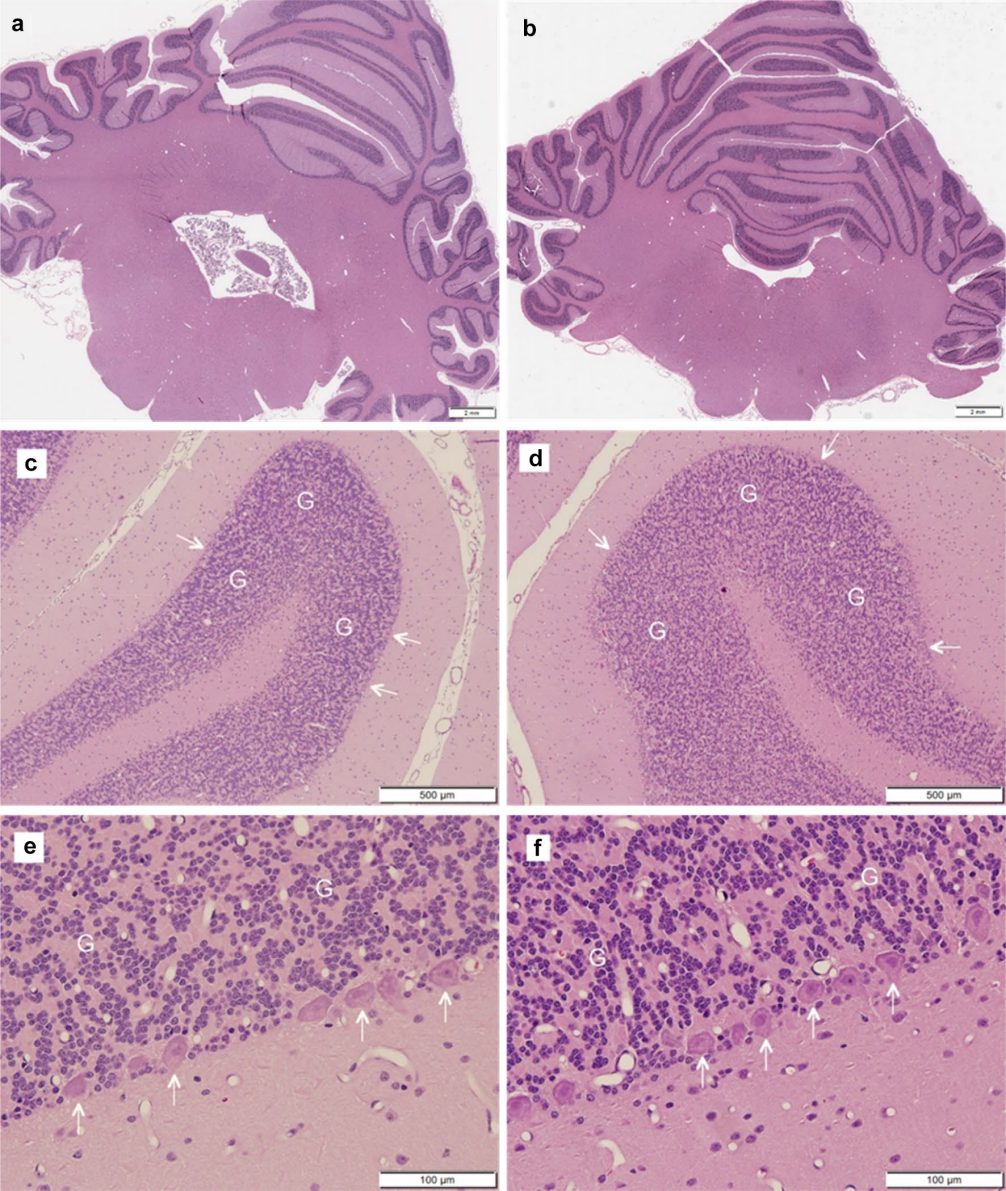
Microscopic findings in cerebellum were not different between control animals (**a, c, e**) and GDNF-treated animals (**b, d, f**). In particular the Purkinje cell layers (arrows) and granule cell layers (G) appeared completely normal in both treatment groups. Staining: H&E

Other than GDNF immunostaining, there were no GDNF-related microscopic findings in cerebellum (Fig. 4) or other parts of the brain, spinal cord, dorsal root ganglia or trigeminal ganglia in any of the GDNF-treated animals.

The original pathology report is provided as Online Resource 1.

### No observed adverse effect level

Based on the results of the study, the GDNF dose tested, 87.1 µg at a *C*_i_ of 0.67 µg/µL administered every 4 weeks for 40 weeks, is considered the no observed adverse effect level (NOAEL) for the intermittent IPu administration of GDNF in rhesus monkeys.

## Discussion

This is the first study to evaluate chronic intermittent bilateral IPu drug administration via CED in nonhuman primates. Intermittent dosing has recently been proposed as the most appropriate delivery paradigm to enable effective use of CED without the risk of significant overflow (“flooding”) (Gimenez et al. 2011; Luz et al. 2016). As assessed by the high rates of infusions that were completed bilaterally (98.2%) or unilaterally (1.8%) over a 9-month period, this study demonstrates the feasibility and tolerability of a novel treatment paradigm in a chronic large animal setting. This potentially opens the door for long-term toxicity studies of other drugs requiring intermittent intraparenchymal CED to directly target neurodegenerative and other serious neurological conditions.

The study also demonstrates the absence of GDNF-related adverse effects and of cerebral or cerebellar toxicity in rhesus monkeys receiving a brain dose of 87.1 µg GDNF (*C*_i_: 0.67 µg/µL) every 4 weeks for a total of 9 months. Cerebellar lesions, in particular Purkinje cell atrophy, were previously observed in rhesus monkeys treated with a 4-week brain dose of 2800 µg GDNF using continuous unilateral IPu infusion for 6 months (*C*_i_: 0.67 µg/µL) (Hovland et al. 2007). In addition, in that study, an increased inflammatory reaction to the foreign protein was seen at the infusion site and along the catheter track with the same dose. Although the lower doses tested in the continuous dosing study were not associated with toxicologically relevant findings, and a 4-week brain dose of 840 µg GDNF (*C*_i_: 0.2 µg/µL) was defined as the NOAEL, the cerebellar toxicity with the high dose was of significant concern (in particular because it was not clinically monitorable and did not produce MRI signals) and contributed to a temporary halt of the GDNF clinical development program in PD in 2004 (Luz et al. 2016).

It has been hypothesized that the cerebellar lesions were caused by acute drug withdrawal after extended leakage-mediated exposure to GDNF *C*_CSF_ > 1700 pg/mL which in turn down-regulated GDNF receptors on Purkinje cells (Luz et al. 2016). This hypothesis is consistent with the observed absence of cerebellar toxicity with IT (monthly) and ICV (biweekly and monthly) bolus administration of GDNF at doses of 2800–9333 µg/4 weeks given for 3–6 months (Hovland et al. 2007; Luz et al. 2016). The hypothesis was considered sufficiently robust to allow the selection of a single dose level for the present study and avoid an unnecessary use of animals.

The dosing scheme was chosen to maximize the probability of producing cerebellar findings without unduly raising the risk of adverse findings at the site of delivery. In the absence of relevant experience with intermittent dosing, the data from continuous IPu dosing studies were applied to define the maximum GDNF *C*_i_ that was considered likely to remain free of local toxicity. In the 6-month continuous IPu dosing toxicity study, the majority of the animals in the high-dose group (*C*_i_ of 0.67 µg/µL) showed MRI findings and histological signs of local edema formation and disruption of the blood brain barrier, presumably due to inflammation following protein accumulation around the catheter tips (Hovland et al. 2007). In another continuous IPu dosing toxicity study in rhesus monkeys, local edema together with focal necrosis and degenerated neurons was found in 2 of 5 animals after 5 weeks of treatment with GDNF at a *C*_i_ of 1.67 µg/µL (Luz et al. 2016). Moreover, higher protein concentrations were shown to cause local toxicity even after single dose administration in rats and rhesus monkeys (Bowenkamp et al. 1995; Gash et al. 1995). Therefore, it was concluded that the GDNF *C*_i_ should not be increased beyond the level of 0.67 µg/µL in the present study. This also took into account that the treatment duration was longer than in any previous toxicity study of GDNF.

As another mechanism to increase dose, *V*_i_ was maximized recognizing that reflux-free IPu infusions in rhesus monkeys can only be achieved with volumes up to 50–70 µL (Varenika et al. 2008). Therefore, the chosen *V*_i_ of 130 µL was anticipated to induce reflux into the CSF compartment. However, since the leading fraction of the infusate was aCSF in all animals, it was expected that most of the drug-containing fraction would remain within the putamen. Using more than one catheter per hemisphere was not feasible for two reasons. First, while a second catheter could have been positioned within the putamen (as evidenced by the successful repeat implantations in the satellite animals), placing the extra tubing and filters on the skull would have unduly increased the risk of kinking, skin erosion or infection, hence posing an unrelated feasibility risk to the study (which was the reason for removing these components in the satellite animals before implanting the second DDS). Second, with two catheters per putamen, further putamenal volume overload could have been avoided only by reducing the *V*_i_ per catheter which, due to the constant dead space per catheter, would necessarily have been done at the cost of the drug-containing fraction of the infusate, thus leading to a paradoxical reduction in the total dose of GDNF.

Although the total 4-week brain dose was more than an order of magnitude smaller than the toxic dose in the 6-month continuous IPu dosing study (87.1 vs. 2800 µg), both mean GDNF *C*_CSF_ and the range of individual GDNF *C*_CSF_ values after the 2nd and final doses were similar to the respective values in the previous study (Hovland et al. 2007). This suggests that there was significant (and, probably due to rapid mixing of the aCSF and GDNF fractions of the infusate in the interstitial space, greater than anticipated) drug leakage into the CSF compartment. As a result, five animals had GDNF *C*_CSF_ values > 1700 pg/mL at both time points and thus, were as close as possible with intermittent delivery to the hypothesized GDNF *C*_CSF_ pattern underlying the cerebellar lesions associated with continuous IPu delivery (Luz et al. 2016). The absence of cerebellar toxicity under these conditions is presumably due to the short (34 h) half-life of GDNF in CSF (Luz et al. 2016), which leads to rapid elimination of the drug from CSF as evidenced by the fact that all pre-dose CSF samples were clear of GDNF. Therefore, cerebellar exposure to GDNF *C*_CSF_ was fluctuating and exposure to peak levels was temporally limited to the first few days following each dosing.

While the difference in the total 4-week brain dose between this and the Hovland et al. (2007) protocol is a potential limitation, it should be noted that the present dose is clinically relevant, while the previous dose was excessive in the context of intermittent dosing. When scaled by a factor 15 to adjust for volume differences between rhesus monkey brain and Parkinsonian human brain (Yin et al. 2009), the human equivalent dose (HED) of the present dose is 1307 µg/4 weeks. This is 5.4 times the dose used in a recently completed Phase 2 study investigating intermittent IPu dosing in PD patients (240 µg/4 weeks; Whone et al., submitted for publication). In contrast, the HED of the dose that resulted in cerebellar toxicity with the continuous dosing paradigm was 42,000 µg/4 weeks or 175 times the clinical dose, and the NOAEL that was defined in the same study (840 µg) translated to 12,600 µg/4 weeks or 52.5 times the clinical dose.

Continuous non-CED infusion is known to induce localized protein build-up at the site of delivery with very high GDNF tissue concentrations even at low GDNF *C*_i_ levels (Salvatore et al. 2006). This, as described above, can lead to local lesions, if the tissue exposure (as defined by the area under the tissue concentration–time curve) is large enough over time. In this context, it is important that the physical and biological half-lives of GDNF in striatum are measured in weeks (Hadaczek et al. 2010; Taylor et al. 2013), which necessarily leads to protein accumulation if large doses are administered continuously. Intermittent CED is associated with a rapid increase of GDNF tissue concentrations in a substantial part of the putamen, leading to a short-term peak exposure of the entire *V*_d_ as a desired effect. However, due the homogeneous convective drug distribution and the long treatment intervals, localized protein build-up is effectively prevented. Therefore, the intermittent dosing paradigm is associated with a lower risk of local tissue reactions than the continuous dosing paradigm, as evidenced by the absence of local toxicity after 9 months in the present study using the same *C*_i_ that previously caused a local tissue reaction with continuous dosing after 6 months.

These findings, together with the confirmation of the hypothesis underlying the previously observed cerebellar lesions, suggest that it is appropriate to use the (higher) NOAEL defined in the 6-month continuous IPu dosing toxicity study rather than the present (lower) NOAEL for the determination of safety margins for clinical use of GDNF in patients with PD.

It is also worth noting that when scaled by factor 15, the *V*_i_ per putamen in the study was approximately 2.4 times the *V*_i_ in the recent Phase 2 study (1950 vs. 800 µL) (Whone et al., submitted for publication). Therefore, the present treatment protocol represented a worst-case scenario in terms of over-infusion, thus testing a clinical contingency. Similarly, the satellite study was performed to assess the potential toxicological implications of catheter repositioning as another clinical contingency that may be associated with increased drug leakage due to added mechanical tissue damage. In view of these purposely designed challenges, the absence of any toxicity in the study is considered clinically robust. Furthermore, it is noteworthy that although the study design facilitated drug leakage into the CSF, the frequencies of antibody formation in serum and CSF against GDNF were lower than previously reported with continuous IPu dosing (Hovland et al. 2007). The immunogenicity of GDNF observed in different study settings and species will be discussed in a separate publication.

In conclusion, intermittent IPu CED for the long-term administration of GDNF at a *C*_i_ of 0.67 µg/µL and a total brain dose of 87.1 µg/4 weeks was not associated with GDNF-related adverse effects, with the exception of anti-GDNF antibody formation and GDNF immunostaining in the brain of GDNF-treated animals, which were both expected. In particular, GDNF did not cause any histopathological findings in the brain, spinal cord, dorsal root ganglia, or trigeminal ganglia. Safety margins for clinical use of GDNF in patients with PD should be determined on the basis of the NOAEL defined in the 6-month continuous IPu dosing toxicity study. While most relevant to the GDNF development program itself, the study may be of significant translational value to other programs that utilize intermittent intraparenchymal CED.

## Electronic supplementary material

Below is the link to the electronic supplementary material.

Supplementary material 1 (PDF 2702 KB)
