# Supplementary material for: Intermittent convection-enhanced delivery of GDNF into rhesus monkey putamen: absence of local or cerebellar toxicity
Source: Arch Toxicol. 2018 May 22;92(7):2353–67. doi: 10.1007/s00204-018-2222-z (PMC6015623; doi:10.1007/s00204-018-2222-z)
Supplement: Supplementary file 1 — Supplementary material 1 (PDF 2702 KB) [file 204_2018_2222_MOESM1_ESM.pdf]

Pathology Report: 15-RS-288
40-Week Toxicity Study of Recombinant-Methionyl Human Glial Cell Line-Derived Neurotrophic Factor (r-metHuGDNF) via Intermittent Bilateral Intraputamenal Convection-Enhanced Delivery in Rhesus Monkeys with a 12-Week Recovery Period

Author

Kristen J. Nikula, DVM, PhD, DACVP, FIATP

Seventh Wave Completion Date

November 24, 2015

Performing Laboratory

Seventh Wave Laboratories LLC
19 Worthington Access Dr.
Maryland Heights, MO 63043, USA

Testing Facility and Study Number

Valley Biosystems, Inc.
West Sacramento, CA 95605, USA
Study Number: S14-10463

Sponsor and Reference Number

MedGenesis Therapeutix, Inc.
Victoria, BC, V8W 3Y7, Canada
Reference Number: MGT03-PRE003

REGULATORY COMPLIANCE STATEMENT

Seventh Wave Number: 15-RS-288
Valley Biosystems Study Number: S14-10463
MedGenesis Therapeutix Reference Number: MGT03-PRE003

Study Title: Pathology Report
40-Week Toxicity Study of Recombinant-Methionyl Human Glial Cell Line-Derived Neurotrophic Factor (r-metHuGDNF) via Intermittent Bilateral Intraputamenal Convection-Enhanced Delivery in Rhesus Monkeys with a 12-Week Recovery Period

Seventh Wave Statement of Regulatory Compliance for Pathology

All portions of this study performed by Seventh Wave Laboratories LLC were in compliance with the FDA Good Laboratory Practice (GLP) Regulations.

The histology and pathology support provided by Seventh Wave were conducted according to the Protocol, Protocol Amendments 1 to 7, and Seventh Wave's Standard Operating Procedures (SOPs).

Approved by:

Kristen J. Nikula (signature) 24 November 2015

Kristen J. Nikula, DVM, PhD, DACVP, FIATP — Date
Study Pathologist
Seventh Wave, Maryland Heights, MO, USA

SIGNATURE OF APPROVAL

Seventh Wave Number: 15-RS-288
Valley Biosystems Study Number: S14-10463
MedGenesis Therapeutix Reference Number: MGT03-PRE003

Study Title: Pathology Report
40-Week Toxicity Study of Recombinant-Methionyl Human Glial Cell Line-Derived Neurotrophic Factor (r-metHuGDNF) via Intermittent Bilateral Intraputamenal Convection-Enhanced Delivery in Rhesus Monkeys with a 12-Week Recovery Period

The results presented in this report accurately reflect the data generated by Seventh Wave in support of this study.

Kristen J Nikula 24 November 2015

Kristen J. Nikula, DVM, PhD, DACVP, FIATP
Study Pathologist
Seventh Wave, Maryland Heights, MO, USA

Date

SEVENTH WAVE QUALITY ASSURANCE STATEMENT

Seventh Wave
Quality Assurance Statement for Pathology Report

Seventh Wave Number: 15-RS-288
Test Facility Study Number: S14-10463
Sponsor Number: MGT03-PRE003

Study Title: 40-Week Toxicity Study of Recombinant-Methionyl Human Glial Cell Line-Derived Neurotrophic Factor (r-metHuGDNF) Via Intermittent Bilateral Intraputamenal Convection-Enhanced Delivery in Rhesus Monkeys with a 12-Week Recovery Period

Reviews conducted by Seventh Wave Quality Assurance confirm that the pathology report reflects the raw data for the portion of the study conducted by Seventh Wave Laboratories LLC. The following reviews were conducted:

Phase Inspected	Audit Date and Auditor	Date Reported to Principal Investigator and Facility Management	Date Reported to Study Director, Study Director's Management and Lead QA
Tissue Embedding and Special Staining	July 21, 2015<br>Patricia A. Hodge	July 21, 2015	July 21, 2015

Materials Audited	Audit Date and Auditor	Date Reported to Principal Investigator and Facility Management	Date Reported to Study Director, Study Director's Management and Lead QA
Histology Study Data	October 27, 2015<br>Jason B. Werner	October 28, 2015	November 9, 2015
Draft Pathology Report	Oct. 30 and Nov. 9, 2015<br>Jason B. Werner	November 9, 2015	November 9, 2015
Final Pathology Report	November 24, 2015<br>Jason B. Werner	November 24, 2015	November 24, 2015

[signature] 24 Nov 2015

Jason B. Werner – Quality Assurance Manager *Date*
Seventh Wave, Maryland Heights, MO, USA

TABLE OF CONTENTS

Section **Page**

TITLE PAGE....1

REGULATORY COMPLIANCE STATEMENT....2

SIGNATURE OF APPROVAL....3

SEVENTH WAVE QUALITY ASSURANCE STATEMENT....4

TABLE OF CONTENTS....5

1.0 GENERAL INFORMATION....6

2.0 METHODS....6

3.0 RESULTS AND DISCUSSION....8

3.1 Macroscopic Observations at Necropsy....8

3.2 Histopathology....8

3.2.1 Terminal and Satellite Sacrifices....8

3.2.2 Recovery Sacrifice....12

3.2.3 Non-r-metHuGDNF-Related, Non-Catheter Track-Related Findings .14

3.3 GDNF Immunohistochemistry....15

4.0 CONCLUSION....15

5.0 REFERENCES....16

6.0 ARCHIVES....17

APPENDIX 1: HISTOPATHOLOGY DATA....18

Table 1-1A. Intergroup Comparison of Histopathology Observations—Terminal....19

Table 1-1B. Intergroup Comparison of Histopathology Observations—Recovery....32

Table 1-1C. Intergroup Comparison of Histopathology Observations—Satellite....49

Table 1-2. Individual Animal Data (Concise Edition)....61

Table 1-3A. Histopathology Cross Reference Table—Terminal....126

Table 1-3B. Histopathology Cross Reference Table —Recovery....149

Table 1-3C. Histopathology Cross Reference Table —Satellite....176

APPENDIX 2: GLOSSARY OF MORPHOLOGIC TERMS AND EXPLANATION OF SEVERITY GRADES....194

APPENDIX 3: DEVIATION REPORT FOR MISSING TISSUES....197

1.0 GENERAL INFORMATION

Histologic tissue processing was conducted by Seventh Wave Laboratories LLC, Chesterfield, MO, USA under the supervision of Kim Shevlin. The pathology evaluation was conducted by Dr. Kristen J. Nikula of Seventh Wave. Results of microscopic evaluations were recorded electronically in Provantis (version 9.1).

The death details (i.e., Day of Death, Day of Necropsy, and Mode of Death), necropsy information, and day on study (i.e., Study Day No.) for each animal as reflected in the appendices were transcribed into the Provantis pathology system by Seventh Wave to facilitate reporting.

Study type as listed in table headers of the Individual Animal Data refers only to Seventh Wave's portion of the study.

Tissues from 20 male Rhesus monkeys were received at Seventh Wave Laboratories for sampling, processing, and slide production. The monkeys were treated via intermittent bilateral intraputamenal convection-enhanced delivery catheters (two per animal, one in each putamen) at a volume of approximately 130 µL/catheter, including 65 µL test or control article and 65 µL diluent (artificial cerebrospinal fluid; aCSF) to clear the dead space of the system.

Study design information received from the Sponsor was as follows:

Group No.	Test Material	Planned Number of Animals			Dosage of Study Treatments	
		Main Study/No Recovery (Necropsy Day 281±10 Days)	Main Study/ Recovery (Necropsy Day 365±10 Days)	Satellite Study/No Recovery (Necropsy Day 281 ±10 Days)	Concentration in Infusate (C_i) (µg/µL)	Total Dose per Treatment Every 4 Weeks (µg)
1	Control	3	3	0	0	0
2	Test Article	5	5	4	0.67[a]	87.1[b]

[a] 0.67 µg/µL dose rationale provided in Protocol Section 12.5.
[b] Value derived from 0.67 µg/µL x 65 µL/catheter x 2 catheters.

2.0 METHODS

Macroscopic observations at necropsy were provided as gross necropsy forms for reference in this pathology report. As these data will be presented elsewhere in the study report, they are not re-presented here.

The following tissues were received after each necropsy in 10% neutral buffered formalin or 4% paraformaldehyde from the testing facility as collected during necropsy: brain including cerebellum (21 cassettes), dorsal root ganglia (cervical, lumbar, thoracic), spinal cord

(cervical, lumbar, thoracic), and trigeminal ganglia. Brain cassettes were labelled by the testing facility to indicate levels of the brain from rostral to caudal. The labelling information included an alphabetic letter and, when applicable, left (lt) or right (rt). Histopathology findings were entered into Provantis by these levels and using these alphabetic and left or right designations. Twenty-two brain (including cerebellum) cassettes were received for animal V001963 and twenty brain cassettes (including cerebellum) were received for animal V002603, along with supporting documentation from the testing facility for these numbers of cassettes.

The spinal cord was trimmed by Seventh Wave to produce three cassettes (cervical, lumbar, thoracic), each with an oblique and transverse section of the respective spinal cord segment. The brain and spinal cord cassettes from all 20 monkeys were embedded in paraffin, sectioned, and at least one slide from each was stained with hematoxylin and eosin (H&E). The dorsal root ganglia and trigeminal ganglia were embedded in glycol methacrylate (GMA) blocks, sectioned, and stained with H&E. In addition, four slides including putamen, substantia nigra, ventral tegmental area, and subthalamic nuclei and two slides of cerebellum per animal, were prepared for staining with Luxol fast blue-periodic acid Schiff (LFB-PAS), Fluoro-Jade C (FJC), anti-glial fibrillary acidic protein (GFAP), and Bielschowsky's silver stain (one set of slides per stain). Positive and negative control tissue structures or sections for these supplemental stains, as applicable, stained appropriately. There were no findings that were unique to these supplemental stains. Microscopic findings entered for each tissue were based on the pathologist's examination and interpretation of all slides present for that tissue. In cases where corroboration by the supplemental stain of a finding observed in the H&E-stained slide aided observation or interpretation, a note regarding this corroboration was added in Provantis.

An additional 19 slides of brain, two slides of cerebellum, and three slides of spinal cord (cervical, thoracic, lumbar), from each animal, were immunohistochemically stained with anti-human glial derived neurotrophic factor (GDNF) to detect GDNF protein. For animals V001963 and V002603, these numbers were adjusted to 20 and 18 sections of brain, respectively, two slides of cerebellum, and three slides of spinal cord.

Subsequent to receipt of the initial tissues, and by Protocol Amendment 5, an additional cassette of cerebellum from each animal was received, routinely processed, and stained with H&E. All H&E-stained slides and slides with supplemental stains (LFB-PAS, FJC, GFAP, Bielschowsky's, and GDNF) for this study were examined by the Study Pathologist and findings were entered in Provantis. A peer-review was conducted by Dr. Christopher Houle, DVM, PhD, DACVP of Pfizer Drug Safety Research & Development.

3.0 RESULTS AND DISCUSSION

3.1 Macroscopic Observations at Necropsy

There were no macroscopic observations recorded on the gross necropsy forms that related to the brain, cerebellum, ganglia, or spinal cord or the catheters other than for V000909 (vehicle control animal; terminal sacrifice) and V002611 (animal given r-metHuGDNF; terminal sacrifice). For animal V000909, the necropsy record documented erosions in the dorsal skull, a small cavitary space with red to dark gray discoloration of the adjacent tissue in block F-lt, a well-demarcated lesion in block G that extended into block H, and a note that the catheter track could be visualized in blocks E – H. These observations correlated with clinical observations in this animal as well as microscopic findings of moderate neutrophilic inflammation, moderate catheter track fibrosis, and tissue reactions around the catheter track (see below). For animal V002611, the necropsy record documented bilateral discoloration proximal to the entry site and extending into the striatum with notes that the lesions were well demarcated, dark red, and that tissue disruption may be attributed to the surgical implantation of the outer guide tube. Remnants of the central catheter were visualized in tissue blocks H and I. These observations correlated with tissue reactions around the catheter site including moderate pigmented macrophages (a contributor to discoloration) and moderate catheter track fibrosis (correlate to demarcation).

3.2 Histopathology

Summarized histopathology data for Terminal, Recovery, and Satellite animals are shown in Appendix 1, Table 1-1A, 1-1B, and 1-1C, respectively. Individual animal data are shown in Appendix 1, Table 1-2. Cross Reference data for Terminal, Recovery, and Satellite animals are shown in Appendix 1, Table 1-3A, 1-3B, and 1-3C, respectively. Appendix 2 is a glossary for the main microscopic findings and an explanation of the grading scale.

3.2.1 Terminal and Satellite Sacrifices

Other than GDNF immunostaining (Section 3.3), there were no microscopic findings that were considered r-metHuGDNF-related in the terminal or satellite sacrifice groups. Table 1 shows the incidence and severity of the main microscopic findings in this study. The severity listed corresponds to the highest severity observed for the finding in any of the multiple sections examined per animal.

Table 1: Incidence and Severity of Main Microscopic Findings – Terminal and Satellite Sacrifices*

	Control - Terminal	GDNF - Terminal		GDNF-Satellite
(incidence / animals per group)				
**CT Fibrosis**	**3 / 3**	**5 / 5**		**4 / 4**
Minimal	-	-		2
Mild	2	3		2
Moderate	1	2		-
**CT Mineralized Material**	**2 / 3**	**5 / 5**		**4 / 4**
Minimal	1	2		4
Mild	1	3		-
**CT Foreign Body Reaction**	**3 / 3**	**5 / 5**		**4 / 4**
Minimal	2	4		3
Mild	1	1		1
**CT Hemorrhage**	**1 / 3**	**1 / 5**		**1 / 4**
Minimal	1	1		1
**CT Pigmented Macrophages**	**3 / 3**	**5 / 5**		**4 / 4**
Minimal	3	1		3
Mild	-	3		1
Moderate	-	1 (V002611)		-
**CT Vacuolated Macrophages**	**1 / 3**	**2 / 5**		**1 / 4**
Minimal	-	1		-
Mild	1	1		1
**CT Infiltration, mononuclear cell**	**3 / 3**	**5 / 5**		**3 / 4**
Minimal	2	4		3
Mild	1	1		-
**CT Infiltration, neutrophil**	**1 / 3**	**0 / 5**		**0 / 4**
Minimal	-	-		-
Mild	-	-		-
Moderate	1 (V000909)	-		-
**CT Infiltration, eosinophil**	**2 / 3**	**5 / 5**		**1 / 4**
Minimal	2	4		1
Mild	-	1		-

*To determine the incidence, main findings were considered present in an animal if they occurred in one or more of the sections examined from that animal. If a finding was present, the severity shown is the highest severity for the finding in the animal. CT = catheter track. Sporadic, incidental, “spontaneous” findings are not listed in this table.

Table 1: Incidence and Severity of Main Microscopic Findings – Terminal and Satellite Sacrifices* (continued)

	Control - Terminal	GDNF - Terminal		GDNF-Satellite
(incidence / animals per group)				
**CT Perivascular cuffs, mononuclear**	**0 / 3**	**0 / 5**		**0 / 4**
Minimal	-	-		-
**CT Perivascular cuffs, mixed cell**	**2 / 3**	**3 / 5**		**1 / 4**
Minimal	1	2		1
Mild	1	1		-
**CT Gliosis/Astrocytosis**	**3 / 3**	**4 / 5**		**3 / 4**
Minimal	2	3		2
Mild	1	-		1
Moderate	-	1 (V002611)		-
**CT Vacuolation, white matter**	**3 / 3**	**3 / 5**		**3 / 4**
Minimal	2	3		2
Mild	1	-		1
**CT Vacuolation, gray matter**	**1 / 3**	**0 / 5**		**1 / 4**
Minimal	-	-		1
Mild	1	-		-
**CT Axon Spheroids**	**1 / 3**	**0 / 5**		**0 / 4**
Minimal	1 (V000909)	-		-
**GDNF Immunostaining**	**0 / 3**	**5 / 5**		**4 / 4**
Minimal	-	-		-
Mild	-	-		-
Moderate	-	4		3
Marked	-	1		1

*To determine the incidence, main findings were considered present in an animal if they occurred in one or more of the sections examined from that animal. If a finding was present, the severity shown is the highest severity for the finding in the animal. CT = catheter track. Sporadic, incidental, "spontaneous" findings are not listed in this table.

Other than GDNF immunostaining, which was observed only in animals administered r-metHuGDNF, microscopic findings were generally consistent with expected responses to an implanted device of this type (Butt, 2011; Polikov, et al., 2005). These findings were confined to the catheter tracks and adjacent neuropil and exhibited the same characteristics and similar incidence and severity across the control group and those administered test article. The only exception to this was moderate neutrophilic inflammation (infiltration, neutrophil) and an associated slightly increased tissue reaction (considered as the combination of mixed cell perivascular cuffs, gliosis/astrocytosis, and gray matter vacuolation) around the right catheter track of one control animal (V000909).

One animal administered r-metHuGDNF (V002611) exhibited an accumulation of pigmented macrophages (correlate to the necropsy observation of discoloration) and a tissue reaction (gliosis/astrogliosis) around the left catheter track that was slightly higher in severity but similar in character to that noted in controls. These findings in animal V002611 may have been related to the clinical observations (Veterinary Care Summary signed by Dr. Diane Stockinger, 10/23/15) that included post-surgical issues associated with the initial implantation of the catheters such as swelling and intermittent fluid accumulation caudal to the incision site. The histopathological findings noted around the left catheter track in this animal were considered consistent with these clinical events. The pigmented macrophages contained pigment consistent with hemosiderin, which is an indicator of previous local hemorrhage around the catheter. Some degree of local hemorrhage is an anticipated event with implantation of the catheter. Accordingly, minimal to mild numbers of pigmented macrophages were noted around the catheter track in all other animals in the study and although they occurred with slightly higher severity (moderate) in V002611 the severity of prior hemorrhage suggested by these pigmented macrophages was still within the range of what might be expected with implantation of the catheter. Despite the slight increase in severity of the pigmented macrophages and surrounding glial reaction around the catheter in this animal, these changes were not of high enough severity to be considered adverse findings particularly given the limited distribution and absence of any associated degenerative findings. r-metHuGDNF was not thought to play a role in the development of these findings given the comparable characteristics of these findings to those noted in many other animals including controls.

None of the findings in the terminal and satellite sacrifice groups were considered adverse based on the limited distribution and severity with the one exception being the moderate neutrophilic inflammation in control animal V000909. The inflammatory process in this animal was considered adverse given its severity level as well as its character, which was highly suggestive of a response to local bacterial infection. This inflammatory process was not considered directly related to the catheter itself but instead specific to this particular animal's clinical history of picking at the sutures and wound associated with the incision site overlying the implanted system. These factors, along with repeated manual manipulation of the external port and surrounding tissues by the animal, likely predisposed the local soft tissue environment around the port to bacterial growth as suggested by the clinical history of a purulent discharge from this area. Details of the clinical observations in this animal are documented in the clinical history (Veterinary Care Summary signed by Dr. Diane Stockinger, 10/23/15). Bacterial growth in this area could serve as a nidus of infection that subsequently tracked along the catheter and into the brain as suggested by the acute inflammatory process observed in this animal.

Overall, the findings observed in the terminal and satellite sacrifice groups were comparable in incidence and severity suggesting that the repositioning of the catheters in the satellite group animals did not exacerbate the anticipated catheter-related findings.

There were no r-metHuGDNF-related or catheter-related microscopic findings in the cerebellum, dorsal root ganglia, trigeminal ganglia, or spinal cord in the terminal or satellite sacrifice groups.

3.2.2 Recovery Sacrifice

Other than GDNF immunostaining (Section 3.3), there were no microscopic findings that were considered r-metHuGDNF-related. Table 2 shows the incidence and severity of the main microscopic findings in this study. The severity listed corresponds to the highest severity observed for the finding in any of the multiple sections examined per animal.

Table 2: Incidence and Severity of Main Microscopic Findings – Recovery Sacrifice*

	**Control - Recovery**	**GDNF - Recovery**
(incidence / animals per group)		
**CT Fibrosis**	**3 / 3**	**5 / 5**
Minimal	1	-
Mild	1	3
Moderate	1	2
**CT Mineralized Material**	**3 / 3**	**4 / 5**
Minimal	3	4
**CT Foreign Body Reaction**	**3 / 3**	**5 / 5**
Minimal	3	4
Mild	-	1
**CT Pigmented Macrophages**	**3 / 3**	**5 / 5**
Minimal	3	3
Mild	-	2
**CT Vacuolated Macrophages**	**1 / 3**	**1 / 5**
Minimal	1	-
Mild	-	1

*To determine the incidence, main findings were considered present in an animal if they occurred in one or more of the sections examined from that animal. If a finding was present, the severity shown is the highest severity for the finding in the animal. CT = catheter track. Sporadic, incidental, "spontaneous" findings are not listed in this table.

Table 2: Incidence and Severity of Main Microscopic Findings – Recovery Sacrifice* (continued)

	Control - Recovery	GDNF - Recovery
(incidence / animals per group)		
**CT Infiltration, mononuclear cell**	**3 / 3**	**4 / 5**
Minimal	2	3
Mild	1	1
**CT Infiltration, neutrophil**	**0 / 3**	**1 / 5**
Minimal	-	-
Mild	-	-
Moderate	-	1 (V002608)
**CT Infiltration, eosinophil**	**2 / 3**	**3 / 5**
Minimal	2	2
Mild	-	1
**CT Perivascular cuffs, mononuclear**	**1 / 3**	**2 / 5**
Minimal	1	2
**CT Perivascular cuffs, mixed cell**	**0 / 3**	**1 / 5**
Minimal	-	1
**CT Gliosis/Astrocytosis**	**3 / 3**	**5 / 5**
Minimal	3	2
Mild	-	3
**CT Vacuolation, white matter**	**2 / 3**	**3 / 5**
Minimal	2	1
Mild	-	1
Moderate	-	1 (V002608)
**CT Vacuolation, gray matter**	**0 / 3**	**2 / 5**
Minimal	-	2
**CT Axon Spheroids**	**0 / 3**	**1 / 5**
Minimal	-	1
**GDNF Immunostaining**	**0 / 3**	**5 / 5**
Minimal	-	-
Mild	-	5

*To determine the incidence, main findings were considered present in an animal if they occurred in one or more of the sections examined from that animal. If a finding was present, the severity shown is the highest severity for the finding in the animal. CT = catheter track. Sporadic, incidental, “spontaneous” findings are not listed in this table.

Microscopic findings were generally consistent with expected responses to an implanted device of this type (Butt, 2011; Polikov, et al, 2005) with the same characteristics and similar incidence and severity in control animals and those given r-metHuGDNF. All findings were confined to the catheter tracks and adjacent neuropil and were not considered adverse based on the limited distribution and severity and/or the lack of associated degenerative changes with the one exception being the moderate neutrophilic inflammation (infiltration, neutrophil) and associated slightly increased tissue reaction (white matter vacuolation and gliosis/astrogliosis) around the catheter track of a single monkey administered r-metHuGDNF (V002608). The findings in this animal were similar to that described with the vehicle control animal (V000909) with an inflammatory process consistent with a response to local bacterial infection. V002608 had a history of issues with the external port requiring repair during the study. V002608 also had a clinical observation of purulent discharge around the port which is likely an indication of a local bacterial infection in adjacent subcutaneous tissues providing a likely source for subsequent tracking of bacteria along the exterior of the catheter into the brain as suggested by the acute inflammatory process observed microscopically in this animal. The findings in V002608 were considered adverse given their severity as well as their character that was suggestive of local bacterial involvement. The findings in V002608 were not considered r-metHuGDNF-related because of the similarities to findings noted in a control animal (V00909). These findings were also not considered a direct result of the catheter system itself but instead a secondary event related to issues with the port which required re-stabilisation surgery. Details of the clinical observations are documented in the clinical history (Veterinary Care Summary signed by Dr. Diane Stockinger, 10/23/15).

Other than the acute inflammatory process described above for a single r-metHuGDNF recovery animal (V002608), findings from the recovery groups had the same characteristics and a similar incidence and severity to those noted in the terminal sacrifice and satellite sacrifice animals. This is consistent with the conclusion that these effects are related to the implantation of, and tissue reaction to, the catheter only and not influenced by r-metHuGDNF administration.

There were no r-metHuGDNF-related or catheter-related microscopic findings in the cerebellum, dorsal root ganglia, trigeminal ganglia, or spinal cord in the recovery sacrifice group.

3.2.3 Non-r-metHuGDNF-Related, Non-Catheter Track-Related Findings

All other microscopic findings in monkeys in the terminal, satellite, or recovery groups were considered spontaneous and/or incidental because they occurred at a sporadic, low incidence and/or occurred at a similar incidence and severity in the control group and those administered r-metHuGDNF, and/or their incidence and severity were as expected for rhesus monkeys. This included a few animals (V001954, V001935, and V001633) with a small number of axon spheroids noted in brain sections away from the catheter track. These axon spheroids were considered incidental, age-related findings as has been previously

reported in nonhuman primates, including rhesus macaques (Fahey and Westmoreland, 2012). They occur most commonly in the globus pallidus, substantia nigra, nucleus gracilis, medial vestibular nucleus, and ventral nucleus of the thalamus (Fahey and Westmoreland, 2012), which corresponds to where they were observed in the study animals.

3.3 GDNF Immunohistochemistry

Positive and negative control sections for GDNF immunohistochemistry stained appropriately. GDNF immunostaining was not observed in the vehicle control animals from the terminal or recovery sacrifice groups. All nine monkeys administered r-metHuGDNF from the terminal and satellite sacrifice groups had multiple sections where GDNF immunostaining was graded as moderate with the immunostaining reaching the marked level in one animal each in the terminal and satellite groups. At the recovery sacrifice, all five monkeys administered r-metHuGDNF had multiple sections where the immunostaining was graded as mild. Therefore, although there was a decrease in GDNF immunostaining during the recovery period, mild GDNF immunostaining persisted over the recovery period. Most often, GDNF immunostaining was in the catheter track and/or in the neuropil adjacent to the catheter track. In four animals from the terminal sacrifice group and one animal in the satellite sacrifice group, minimal to moderate GDNF immunostaining was observed in sections where a catheter track was not identified.

4.0 CONCLUSION

Other than GDNF immunostaining, intermittent administration of r-metHuGDNF to Rhesus monkeys via bilateral intraputamenal convection-enhanced delivery catheters at a volume of 65 µL/catheter (0.67 µg/µL), followed by 65 µL aCSF, every four weeks for a total of 11 doses did not result in any r-metHuGDNF related microscopic findings in the brain (including cerebellum), spinal cord, dorsal root ganglia or trigeminal ganglia. GDNF immunostaining demonstrated immunohistochemically was noted in the brain of all r-metHuGDNF-treated animals including recovery animals where the staining intensity was mildly reduced relative to terminal sacrifice animals.

Microscopic findings related to the implantation of, and tissue reaction to, the intraputamenal catheters were noted in all animals but were generally limited in distribution to the catheter tract and immediately surrounding tissues and were largely consistent with the anticipated response to the implantation of this type of device. These findings were not considered adverse due to their limited distribution and severity and there were no discernible differences in character, incidence and severity across the vehicle control and r-metHuGDNF treatment groups with only a few notable exceptions, each of which was associated with the clinical history in these particular animals.

Two of these animals, including a control (V000909) and an animal administered r-metHuGDNF (V002608), exhibited moderate neutrophilic inflammation in and around the catheter track in the brain with a mild to moderate tissue reaction in the surrounding neuropil (such as white matter vacuolation and/or gliosis/astrogliosis). Given the severity,

and that the character of this inflammation was suggestive of local bacterial infection, these findings were considered adverse but they were not considered r-metHuGDNF-related or directly associated with the catheter itself. These findings were unique to these two animals and they were associated with a common clinical history involving chronic issues with the external port and surrounding soft tissues including purulent discharge. One of these animals (V000909) demonstrated repeated manual manipulation of the tissue overlying the implanted system which perpetuated and exacerbated the issue. As such, these findings were not considered directly the result of the catheter itself but instead related to the chronic clinical issues associated with the tissue overlying the extracranial parts of the device.

Another exception was an r-metHuGDNF administered terminal sacrifice animal (V002611) that had findings consistent in character with all the other animals in the study, but with a slightly higher severity of gliosis/astrocytosis and pigmented macrophages around the catheter track. The pigment was consistent with hemosiderin, which is an indicator of previous hemorrhage, and some degree of local hemorrhage is an anticipated event with catheter implantation. These findings were associated with clinical observations including post surgical swelling and intermittent fluid accumulation caudal to the incision site. These clinical observations may have been indicative of similar underlying issues along the catheter track as was suggested by the slightly enhanced microscopic findings noted in this animal. As such, the slight enhancement of these findings was not considered r-metHuGDNF related, particularly considering the occurrence in just a single animal and the overall consistency in character with findings noted in vehicle control and other r-metHuGDNF treated animals. The findings in this animal were also not considered adverse given the limited distribution, the lack of associated degenerative findings, and the limited severity, even though it was slightly increased over other animals

In conclusion, there were no r-metHuGDNF-related histopathology findings noted in this study, with the exception of GDNF immunostaining present in the brain of r-metHuGDNF-treated animals as anticipated. All other notable findings were localized to the catheter track and generally of the type, incidence and severity anticipated with the implantation of this type of device. The only adverse findings noted in the study were not r-metHuGDNF-related as they occurred in a vehicle control terminal sacrifice animal and in an r-metHuGDNF-treated recovery sacrifice animal and they were also not considered directly related to the catheter itself but instead secondary to chronic clinical issues associated with the external port or soft tissues in these two particular animals.

5.0 REFERENCES

Butt, M.T. Morphologic changes associated with intrathecal catheters for direct delivery to the central nervous system in preclinical studies. *Toxicologic Pathology,* 39: 213-219, 2011.

Fahey, M.A. and Westmoreland, S.V. Nervous System Disorders of Nonhuman Primates and Research Models, Chapter 15. In: Nonhuman Primates in Biomedical Research, 2nd

edition, Volume 2, eds. C.R. Abee, K. Mansfield, S. Tardif, and T. Morris. Elsevier, Inc., 2012.

Polikov, V.S., Tresco, P.A., Reichert, W.M. Response of brain tissue to chronically implanted neural electrodes. *Journal of Neuroscience Methods,* 148: 1-18, 2005.

6.0 ARCHIVES

The final pathology report will be shipped to the Study Director. Associated raw data, slides, blocks, and remaining wet tissue will be temporarily retained at Seventh Wave until transferred to the Testing Facility.

APPENDIX 1: HISTOPATHOLOGY DATA

The death details (i.e., Day of Death, Day of Necropsy, and Mode of Death), necropsy information, and day on study (i.e., Study Day No.) for each animal as reflected in the appendices were transcribed into the Provantis pathology system by Seventh Wave to facilitate reporting.

Table 1-1A. Intergroup Comparison of Histopathology Observations—Terminal

PTA005-05/00 Provantis Version 9.1 Date: 11/24/2015 9:44 Page: 1

Pathology - Intergroup Comparison of Histopathology Observations; Terminal
15-RS-288 - 40-Week Toxicity Stud of Recombinant-Methionyl Human Glial Cell Line-Derived Neurotrophic Factor (r-metHuGDNF) via Intermittent Bilateral Intraputamenal Convection-Enhanced Delivery in Rhesus Monkeys with a 12-Week Recovery Period

Observations: Neo-Plastic and Non Neo-Plastic

Removal Reason: Terminal Sacrifice

	----- MALES -----	
	MC	MG
	0 µg	87.1 µg
Number of Animals on Study :	3	5
Number of Animals Completed:	(3)	(5)
BRAIN D;		
Examined	(3)	(5)
Within Normal Limits	3	4
CT MINERALIZED MATERIAL	(0)	(1)
Minimal	0	1
BS/PONS O-LT;		
Examined	(0)	(3)
Within Normal Limits	0	3
BS/PONS O-RT;		
Examined	(0)	(3)
Within Normal Limits	0	3
BS/PONS P-LT;		
Examined	(3)	(2)
Within Normal Limits	1	1
FIBROSIS; Meninges	(2)	(1)
Minimal	2	1
BS/PONS P-RT;		
Examined	(3)	(2)
Within Normal Limits	2	1
FIBROSIS; Meninges	(1)	(1)
Minimal	0	1
Mild	1	0
CB/MO T-CB;		
Examined	(3)	(5)
Within Normal Limits	3	5
CB/MO T-LT;		
Examined	(3)	(5)
Within Normal Limits	2	5
FIBROSIS; Meninges	(1)	(0)
Mild	1	0

Pathology - Intergroup Comparison of Histopathology Observations; Terminal
15-RS-288 - 40-Week Toxicity Stud of Recombinant-Methionyl Human Glial Cell Line-Derived Neurotrophic Factor (r-metHuGDNF) via Intermittent Bilateral Intraputamenal Convection-Enhanced Delivery in Rhesus Monkeys with a 12-Week Recovery Period

Observations: Neo-Plastic and Non Neo-Plastic

Removal Reason: Terminal Sacrifice

	MALES	
	MC 0 µg	MG 87.1 µg
Number of Animals on Study :	3	5
Number of Animals Completed:	(3)	(5)
**CB/MO T-RT;**		
Examined	(3)	(5)
Within Normal Limits	3	5
**CB R-CB;**		
Examined	(3)	(5)
Within Normal Limits	3	5
**CB U-CB;**		
Examined	(0)	(1)
Within Normal Limits	0	1
**CB V-CB;**		
Examined	(3)	(4)
Within Normal Limits	3	4
**CD F-LT;**		
Examined	(3)	(5)
Within Normal Limits	2	2
CATHETER TRACK (CT)	1	2
CT VACUOLATED MACROPHAGES	(1)	(0)
Mild	1	0
CT PIGMENTED MACROPHAGES	(1)	(2)
Minimal	1	1
Moderate	0	1
CT INFILTRATION, MONONUCLEAR CELL	(1)	(0)
Minimal	1	0
CT GLIOSIS/ASTROCYTOSIS	(1)	(2)
Minimal	0	1
Mild	1	1
FIBROSIS; Meninges	(1)	(0)
Minimal	1	0
CT HEMORRHAGE	(1)	(0)
Minimal	1	0
CT FIBROSIS	(0)	(1)
Minimal	0	1
CT INFILTRATION, EOSINOPHIL	(0)	(1)

Pathology - Intergroup Comparison of Histopathology Observations; Terminal
15-RS-288 - 40-Week Toxicity Stud of Recombinant-Methionyl Human Glial Cell Line-Derived Neurotrophic Factor (r-metHuGDNF) via Intermittent Bilateral Intraputamenal Convection-Enhanced Delivery in Rhesus Monkeys with a 12-Week Recovery Period

Observations: Neo-Plastic and Non Neo-Plastic

Removal Reason: Terminal Sacrifice

	----- MALES -----	
	MC 0 µg	MG 87.1 µg
Number of Animals on Study :	3	5
Number of Animals Completed:	(3)	(5)
CD F-LT; (continued)		
Minimal	0	1
CT AXON SPHEROIDS	(1)	(0)
Minimal	1	0
CT MINERALIZED MATERIAL	(0)	(1)
Minimal	0	1
GDNF IMMUNOSTAINING	(0)	(3)
Minimal	0	1
Mild	0	1
Moderate	0	1
CD F-RT;		
Examined	(3)	(5)
Within Normal Limits	2	0
FIBROSIS; Meninges	(0)	(1)
Minimal	0	1
CATHETER TRACK (CT)	1	3
CT INFILTRATION, NEUTROPHIL	(1)	(0)
Mild	1	0
CT FOREIGN BODY REACTION	(1)	(1)
Minimal	0	1
Mild	1	0
CT FIBROSIS	(1)	(2)
Minimal	1	2
CT HEMORRHAGE	(1)	(0)
Minimal	1	0
CT GLIOSIS/ASTROCYTOSIS	(1)	(2)
Minimal	0	2
Mild	1	0
CT INFILTRATION, MONONUCLEAR CELL	(1)	(1)
Minimal	1	1
CT VACUOLATED MACROPHAGES	(1)	(1)
Minimal	0	1
Mild	1	0
CT PIGMENTED MACROPHAGES	(0)	(2)
Minimal	0	1
Mild	0	1

Pathology - Intergroup Comparison of Histopathology Observations; Terminal
15-RS-288 - 40-Week Toxicity Stud of Recombinant-Methionyl Human Glial Cell Line-Derived Neurotrophic Factor (r-metHuGDNF) via Intermittent Bilateral Intraputamenal Convection-Enhanced Delivery in Rhesus Monkeys with a 12-Week Recovery Period

Observations: Neo-Plastic and Non Neo-Plastic

Removal Reason: Terminal Sacrifice

	----- MALES -----	
	MC 0 µg	MG 87.1 µg
Number of Animals on Study :	3	5
Number of Animals Completed:	(3)	(5)
CD F-RT; (continued)		
CT INFILTRATION, EOSINOPHIL	(0)	(1)
Minimal	0	1
CT VACUOLATION, WHITE MATTER	(1)	(0)
Minimal	1	0
CT MINERALIZED MATERIAL	(0)	(3)
Minimal	0	2
Mild	0	1
GDNF IMMUNOSTAINING	(0)	(4)
Mild	0	1
Moderate	0	3
CD/PUT G-LT;		
Examined	(3)	(5)
Within Normal Limits	1	0
FIBROSIS; Meninges	(1)	(0)
Minimal	1	0
CATHETER TRACK (CT)	2	5
CT VACUOLATED MACROPHAGES	(1)	(0)
Mild	1	0
CT PIGMENTED MACROPHAGES	(1)	(2)
Minimal	1	1
Moderate	0	1
CT INFILTRATION, MONONUCLEAR CELL	(1)	(0)
Minimal	1	0
CT GLIOSIS/ASTROCYTOSIS	(1)	(2)
Minimal	0	1
Mild	1	1
CT FIBROSIS	(1)	(1)
Minimal	1	0
Moderate	0	1
CT INFILTRATION, EOSINOPHIL	(0)	(1)
Minimal	0	1
CT FOREIGN BODY REACTION	(0)	(3)
Minimal	0	3
CT VACUOLATION, WHITE MATTER	(0)	(1)
Minimal	0	1

Pathology - Intergroup Comparison of Histopathology Observations; Terminal
15-RS-288 - 40-Week Toxicity Stud of Recombinant-Methionyl Human Glial Cell Line-Derived Neurotrophic Factor (r-metHuGDNF) via Intermittent Bilateral Intraputamenal Convection-Enhanced Delivery in Rhesus Monkeys with a 12-Week Recovery Period

Observations: Neo-Plastic and Non Neo-Plastic

Removal Reason: Terminal Sacrifice

	MALES	
	MC 0 µg	MG 87.1 µg
Number of Animals on Study :	3	5
Number of Animals Completed:	(3)	(5)
**CD/PUT G-LT; (continued)**		
CT MINERALIZED MATERIAL	(1)	(4)
Minimal	0	3
Mild	1	1
GDNF IMMUNOSTAINING	(0)	(4)
Minimal	0	1
Mild	0	1
Moderate	0	2
**CD/PUT G-RT;**		
Examined	(3)	(5)
Within Normal Limits	1	0
FIBROSIS; Meninges	(1)	(0)
Minimal	1	0
CATHETER TRACK (CT)	2	5
CT FIBROSIS	(1)	(4)
Minimal	0	3
Moderate	1	1
CT FOREIGN BODY REACTION	(2)	(5)
Minimal	2	4
Mild	0	1
CT GLIOSIS/ASTROCYTOSIS	(1)	(3)
Minimal	0	2
Mild	1	1
CT INFILTRATION, MONONUCLEAR CELL	(1)	(3)
Minimal	0	3
Mild	1	0
CT INFILTRATION, NEUTROPHIL	(1)	(0)
Mild	1	0
CT VACUOLATED MACROPHAGES	(1)	(0)
Mild	1	0
CT PIGMENTED MACROPHAGES	(1)	(5)
Minimal	1	4
Mild	0	1
CT VACUOLATION, WHITE MATTER	(1)	(0)
Minimal	1	0
CT INFILTRATION, EOSINOPHIL	(0)	(2)

Pathology - Intergroup Comparison of Histopathology Observations; Terminal
15-RS-288 - 40-Week Toxicity Stud of Recombinant-Methionyl Human Glial Cell Line-Derived Neurotrophic Factor (r-metHuGDNF) via Intermittent Bilateral Intraputamenal Convection-Enhanced Delivery in Rhesus Monkeys with a 12-Week Recovery Period

Observations: Neo-Plastic and Non Neo-Plastic

Removal Reason: Terminal Sacrifice

	----- MALES -----	
	MC<br>0 µg	MG<br>87.1 µg
Number of Animals on Study :	3	5
Number of Animals Completed:	(3)	(5)
CD/PUT G-RT; (continued)		
Minimal	0	2
CT MINERALIZED MATERIAL	(0)	(3)
Minimal	0	2
Mild	0	1
GDNF IMMUNOSTAINING	(0)	(5)
Mild	0	2
Moderate	0	2
Marked	0	1
CD/PUT H-LT;		
Examined	(3)	(5)
Within Normal Limits	1	0
CATHETER TRACK (CT)	2	5
CT FIBROSIS	(2)	(3)
Minimal	2	2
Moderate	0	1
CT GLIOSIS/ASTROCYTOSIS	(2)	(3)
Minimal	2	2
Moderate	0	1
CT INFILTRATION, MONONUCLEAR CELL	(1)	(3)
Minimal	1	3
CT PIGMENTED MACROPHAGES	(2)	(4)
Minimal	2	1
Mild	0	2
Moderate	0	1
CT VACUOLATED MACROPHAGES	(1)	(1)
Mild	1	1
CT INFILTRATION, EOSINOPHIL	(0)	(2)
Minimal	0	1
Mild	0	1
CT FOREIGN BODY REACTION	(0)	(4)
Minimal	0	4
INFILTRATION; Mononuclear cell; Meninges	(0)	(2)
Minimal	0	2
CT VACUOLATION, WHITE MATTER	(0)	(1)
Minimal	0	1

Pathology - Intergroup Comparison of Histopathology Observations; Terminal
15-RS-288 - 40-Week Toxicity Stud of Recombinant-Methionyl Human Glial Cell Line-Derived Neurotrophic Factor (r-metHuGDNF) via Intermittent Bilateral Intraputamenal Convection-Enhanced Delivery in Rhesus Monkeys with a 12-Week Recovery Period

Observations: Neo-Plastic and Non Neo-Plastic

Removal Reason: Terminal Sacrifice

	MALES MC 0 µg	MALES MG 87.1 µg
Number of Animals on Study :	3	5
Number of Animals Completed:	(3)	(5)
CD/PUT H-LT; (continued)		
CT PERIVASCULAR CUFFS; Mixed	(0)	(1)
Minimal	0	1
CT MINERALIZED MATERIAL	(0)	(3)
Minimal	0	3
GDNF IMMUNOSTAINING	(0)	(4)
Moderate	0	3
Marked	0	1
CD/PUT H-RT;		
Examined	(3)	(5)
Within Normal Limits	1	0
CATHETER TRACK (CT)	2	5
CT FIBROSIS	(2)	(3)
Minimal	1	0
Mild	0	2
Moderate	1	1
CT GLIOSIS/ASTROCYTOSIS	(1)	(3)
Minimal	0	2
Mild	1	1
CT INFILTRATION, MONONUCLEAR CELL	(1)	(3)
Minimal	0	3
Mild	1	0
CT INFILTRATION, NEUTROPHIL	(1)	(0)
Moderate	1	0
CT PIGMENTED MACROPHAGES	(1)	(5)
Minimal	1	3
Mild	0	2
CT VACUOLATED MACROPHAGES	(1)	(1)
Minimal	0	1
Mild	1	0
CT PERIVASCULAR CUFFS; Mixed	(1)	(1)
Minimal	0	1
Mild	1	0
CT VACUOLATION, WHITE MATTER	(1)	(1)
Minimal	0	1
Mild	1	0

Pathology - Intergroup Comparison of Histopathology Observations; Terminal
15-RS-288 - 40-Week Toxicity Stud of Recombinant-Methionyl Human Glial Cell Line-Derived Neurotrophic Factor (r-metHuGDNF) via Intermittent Bilateral Intraputamenal Convection-Enhanced Delivery in Rhesus Monkeys with a 12-Week Recovery Period

Observations: Neo-Plastic and Non Neo-Plastic

Removal Reason: Terminal Sacrifice

	MALES MC 0 µg	MALES MG 87.1 µg
Number of Animals on Study :	3	5
Number of Animals Completed:	(3)	(5)
**CD/PUT H-RT; (continued)**		
CT FOREIGN BODY REACTION	(0)	(3)
Minimal	0	3
CT INFILTRATION, EOSINOPHIL	(0)	(4)
Minimal	0	4
CT MINERALIZED MATERIAL	(1)	(2)
Minimal	1	2
GDNF IMMUNOSTAINING	(0)	(5)
Moderate	0	5
**CD/PUT/AC I-LT;**		
Examined	(3)	(5)
Within Normal Limits	0	0
INFILTRATION; Mixed; Meninges	(0)	(1)
Minimal	0	1
CATHETER TRACK (CT)	3	4
CT FIBROSIS	(2)	(2)
Minimal	1	0
Mild	1	2
CT PERIVASCULAR CUFFS; Mixed	(1)	(2)
Minimal	1	1
Mild	0	1
CT PIGMENTED MACROPHAGES	(2)	(3)
Minimal	2	2
Mild	0	1
CT FOREIGN BODY REACTION	(1)	(3)
Minimal	1	3
CT VACUOLATION, WHITE MATTER	(2)	(2)
Minimal	2	2
CT INFILTRATION, MONONUCLEAR CELL	(2)	(3)
Minimal	2	3
CT INFILTRATION, EOSINOPHIL	(2)	(4)
Minimal	2	4
CT GLIOSIS/ASTROCYTOSIS	(1)	(3)
Minimal	1	2
Mild	0	1
CT VACUOLATED MACROPHAGES	(0)	(1)

Pathology - Intergroup Comparison of Histopathology Observations; Terminal
15-RS-288 - 40-Week Toxicity Stud of Recombinant-Methionyl Human Glial Cell Line-Derived Neurotrophic Factor (r-metHuGDNF) via Intermittent Bilateral Intraputamenal Convection-Enhanced Delivery in Rhesus Monkeys with a 12-Week Recovery Period

Observations: Neo-Plastic and Non Neo-Plastic

Removal Reason: Terminal Sacrifice

	MALES MC 0 µg	MALES MG 87.1 µg
Number of Animals on Study :	3	5
Number of Animals Completed:	(3)	(5)
**CD/PUT/AC I-LT; (continued)**		
Minimal	0	1
CT MINERALIZED MATERIAL	(1)	(1)
Minimal	1	1
GDNF IMMUNOSTAINING	(0)	(5)
Mild	0	1
Moderate	0	4
**CD/PUT/GP J-LT;**		
Examined	(3)	(5)
Within Normal Limits	3	1
CATHETER TRACK (CT)	0	1
CT FOREIGN BODY REACTION	(0)	(1)
Minimal	0	1
CT PIGMENTED MACROPHAGES	(0)	(1)
Minimal	0	1
CT FIBROSIS	(0)	(1)
Mild	0	1
CT INFILTRATION, MONONUCLEAR CELL	(0)	(1)
Mild	0	1
CT HEMORRHAGE	(0)	(1)
Minimal	0	1
CT MINERALIZED MATERIAL	(0)	(1)
Minimal	0	1
GDNF IMMUNOSTAINING	(0)	(4)
Minimal	0	1
Mild	0	2
Moderate	0	1
**CD/PUT/GP J-RT;**		
Examined	(3)	(5)
Within Normal Limits	1	2
CATHETER TRACK (CT)	2	1
CT GLIOSIS/ASTROCYTOSIS	(1)	(0)
Minimal	1	0
CT INFILTRATION, MONONUCLEAR CELL	(1)	(1)
Minimal	1	1

Pathology - Intergroup Comparison of Histopathology Observations; Terminal
15-RS-288 - 40-Week Toxicity Stud of Recombinant-Methionyl Human Glial Cell Line-Derived Neurotrophic Factor (r-metHuGDNF) via Intermittent Bilateral Intraputamenal Convection-Enhanced Delivery in Rhesus Monkeys with a 12-Week Recovery Period

Observations: Neo-Plastic and Non Neo-Plastic

Removal Reason: Terminal Sacrifice

	MALES	
	MC 0 µg	MG 87.1 µg
Number of Animals on Study :	3	5
Number of Animals Completed:	(3)	(5)
**CD/PUT/GP J-RT; (continued)**		
CT PERIVASCULAR CUFFS; Mixed	(1)	(0)
Mild	1	0
CT VACUOLATION, WHITE MATTER	(1)	(0)
Mild	1	0
CT FIBROSIS	(1)	(0)
Mild	1	0
CT FOREIGN BODY REACTION	(1)	(1)
Minimal	1	1
CT PIGMENTED MACROPHAGES	(0)	(1)
Minimal	0	1
CT VACUOLATION, GRAY MATTER	(1)	(0)
Mild	1	0
GDNF IMMUNOSTAINING	(0)	(3)
Mild	0	2
Moderate	0	1
**PUT/GP K-LT;**		
Examined	(3)	(5)
Within Normal Limits	3	3
INFILTRATION; Mononuclear cell; Meninges	(0)	(1)
Minimal	0	1
FIBROSIS; Meninges	(0)	(1)
Mild	0	1
GDNF IMMUNOSTAINING	(0)	(1)
Mild	0	1
**PUT/GP K-RT;**		
Examined	(3)	(5)
Within Normal Limits	2	1
VACUOLATION; White matter	(1)	(0)
Minimal	1	0
INFILTRATION; Mononuclear cell; Choroid plexus	(0)	(2)
Minimal	0	2
AXON SPHEROIDS	(0)	(1)
Minimal	0	1
GDNF IMMUNOSTAINING	(0)	(1)

Pathology - Intergroup Comparison of Histopathology Observations; Terminal
15-RS-288 - 40-Week Toxicity Stud of Recombinant-Methionyl Human Glial Cell Line-Derived Neurotrophic Factor (r-metHuGDNF) via Intermittent Bilateral Intraputamenal Convection-Enhanced Delivery in Rhesus Monkeys with a 12-Week Recovery Period

Observations: Neo-Plastic and Non Neo-Plastic

Removal Reason: Terminal Sacrifice

	MALES MC 0 µg	MALES MG 87.1 µg
Number of Animals on Study :	3	5
Number of Animals Completed:	(3)	(5)
PUT/GP K-RT; (continued)		
Mild	0	1
SN N;		
Examined	(3)	(5)
Within Normal Limits	3	5
THAL/SN M;		
Examined	(3)	(5)
Within Normal Limits	3	3
AXON SPHEROIDS	(0)	(1)
Minimal	0	1
GDNF IMMUNOSTAINING	(0)	(2)
Minimal	0	1
Mild	0	1
THAL/STN L;		
Examined	(3)	(5)
Within Normal Limits	1	4
VACUOLATION; White matter	(1)	(0)
Minimal	1	0
VACUOLATION; White matter; Bilateral	(1)	(0)
Minimal	1	0
INFILTRATION; Mononuclear cell; Meninges	(0)	(1)
Minimal	0	1
THAL/STN L1-LT;		
Examined	(1)	(0)
Within Normal Limits	0	0
INFILTRATION; Mononuclear cell; Meninges	(1)	(0)
Minimal	1	0
SPINAL CORD, CERVICAL;		
Examined	(3)	(5)
Within Normal Limits	3	5

Pathology - Intergroup Comparison of Histopathology Observations; Terminal
15-RS-288 - 40-Week Toxicity Stud of Recombinant-Methionyl Human Glial Cell Line-Derived Neurotrophic Factor (r-metHuGDNF) via Intermittent Bilateral Intraputamenal Convection-Enhanced Delivery in Rhesus Monkeys with a 12-Week Recovery Period

Observations: Neo-Plastic and Non Neo-Plastic

Removal Reason: Terminal Sacrifice

	MALES	
	MC<br>0 µg	MG<br>87.1 µg
Number of Animals on Study :	3	5
Number of Animals Completed:	(3)	(5)
**SPINAL CORD, THORACIC;**		
Examined	(3)	(5)
Within Normal Limits	3	5
**SPINAL CORD, LUMBAR;**		
Examined	(3)	(5)
Within Normal Limits	3	5
**DRG, CERVICAL;**		
Examined	(3)	(5)
Within Normal Limits	3	4
VACUOLATION; Ganglion cell	(0)	(1)
Minimal	0	1
**DRG, THORACIC;**		
Examined	(3)	(5)
Within Normal Limits	2	3
VACUOLATION; Ganglion cell	(1)	(1)
Minimal	1	0
Mild	0	1
MINERALIZATION	(0)	(1)
Minimal	0	1
**DRG, LUMBAR;**		
Examined	(3)	(5)
Within Normal Limits	3	4
VACUOLATION; Ganglion cell	(0)	(1)
Minimal	0	1
**TRIGEMINAL GANGLIA;**		
Examined	(3)	(5)
Within Normal Limits	2	5
MINERALIZATION	(1)	(0)
Minimal	1	0

Pathology - Intergroup Comparison of Histopathology Observations; Terminal
15-RS-288 - 40-Week Toxicity Stud of Recombinant-Methionyl Human Glial Cell
Line-Derived Neurotrophic Factor (r-metHuGDNF) via Intermittent Bilateral
Intraputamenal Convection-Enhanced Delivery in Rhesus Monkeys with a 12-Week
Recovery Period

End Of Print

Table 1-1B. Intergroup Comparison of Histopathology Observations—Recovery

PTA005-05/00 Provantis Version 9.1 Date: 11/24/2015 9:44 Page: 1

Pathology - Intergroup Comparison of Histopathology Observations; Recovery
15-RS-288 - 40-Week Toxicity Stud of Recombinant-Methionyl Human Glial Cell Line-Derived Neurotrophic Factor (r-metHuGDNF) via Intermittent Bilateral Intraputamenal Convection-Enhanced Delivery in Rhesus Monkeys with a 12-Week Recovery Period

Observations: Neo-Plastic and Non Neo-Plastic

Removal Reason: Recovery Sacrifice

	MALES MC 0 µg	MALES MG 87.1 µg
Number of Animals on Study :	3	5
Number of Animals Completed:	(3)	(5)
**BRAIN D;**		
Examined	(3)	(5)
Within Normal Limits	2	4
FIBROSIS; Meninges	(1)	(0)
Minimal	1	0
INFILTRATION; Mononuclear cell; Meninges	(1)	(1)
Minimal	1	1
**BS/PONS N-LT;**		
Examined	(0)	(1)
Within Normal Limits	0	1
**BS/PONS N-RT;**		
Examined	(0)	(1)
Within Normal Limits	0	1
**BS/PONS O-LT;**		
Examined	(3)	(5)
Within Normal Limits	3	4
**BS/PONS O-RT;**		
Examined	(3)	(5)
Within Normal Limits	3	5
**CB/MO T-CB;**		
Examined	(0)	(1)
Within Normal Limits	0	0
INFILTRATION; Mononuclear cell; Meninges	(0)	(1)
Minimal	0	1
INFILTRATION; Mononuclear cell; Perivascular	(0)	(1)
Minimal	0	1
**CB/MO T-LT;**		
Examined	(0)	(1)
Within Normal Limits	0	1

Pathology - Intergroup Comparison of Histopathology Observations; Recovery
15-RS-288 - 40-Week Toxicity Stud of Recombinant-Methionyl Human Glial Cell Line-Derived Neurotrophic Factor (r-metHuGDNF) via Intermittent Bilateral Intraputamenal Convection-Enhanced Delivery in Rhesus Monkeys with a 12-Week Recovery Period

Observations: Neo-Plastic and Non Neo-Plastic

Removal Reason: Recovery Sacrifice

	MALES	
	MC 0 µg	MG 87.1 µg
Number of Animals on Study :	3	5
Number of Animals Completed:	(3)	(5)
**CB/MO T-RT;**		
Examined	(0)	(1)
Within Normal Limits	0	1
**CB/MO U-CB;**		
Examined	(3)	(4)
Within Normal Limits	3	4
**CB/MO U-LT;**		
Examined	(3)	(4)
Within Normal Limits	3	4
**CB/MO U-RT;**		
Examined	(3)	(4)
Within Normal Limits	3	3
FIBROSIS; Meninges	(0)	(1)
Mild	0	1
**CB R-CB;**		
Examined	(3)	(5)
Within Normal Limits	3	4
DEGENERATION; Axon	(0)	(1)
Minimal	0	1
**CB V-CB;**		
Examined	(3)	(5)
Within Normal Limits	3	5
**CD F-LT;**		
Examined	(1)	(1)
Within Normal Limits	0	0
CATHETER TRACK (CT)	1	1
CT PIGMENTED MACROPHAGES	(0)	(1)
Minimal	0	1
CT INFILTRATION, MONONUCLEAR CELL	(1)	(1)
Minimal	1	1
FIBROSIS; Meninges	(0)	(1)

Pathology - Intergroup Comparison of Histopathology Observations; Recovery
15-RS-288 - 40-Week Toxicity Stud of Recombinant-Methionyl Human Glial Cell Line-Derived Neurotrophic Factor (r-metHuGDNF) via Intermittent Bilateral Intraputamenal Convection-Enhanced Delivery in Rhesus Monkeys with a 12-Week Recovery Period

Observations: Neo-Plastic and Non Neo-Plastic

Removal Reason: Recovery Sacrifice

	----- MALES -----	
	MC<br>0 µg	MG<br>87.1 µg
Number of Animals on Study :	3	5
Number of Animals Completed:	(3)	(5)
**CD F-LT; (continued)**		
Minimal	0	1
CT FIBROSIS	(0)	(1)
Moderate	0	1
CT INFILTRATION, EOSINOPHIL	(1)	(1)
Minimal	1	1
CT FOREIGN BODY REACTION	(1)	(1)
Minimal	1	1
CT PERIVASCULAR CUFFS; Mononuclear cell	(1)	(0)
Minimal	1	0
CT VACUOLATION, GRAY MATTER	(0)	(1)
Minimal	0	1
CT MINERALIZED MATERIAL	(0)	(1)
Minimal	0	1
GDNF IMMUNOSTAINING	(0)	(1)
Mild	0	1
**CD F-RT;**		
Examined	(1)	(1)
Within Normal Limits	0	0
FIBROSIS; Meninges	(0)	(1)
Minimal	0	1
CATHETER TRACK (CT)	1	0
CT FOREIGN BODY REACTION	(1)	(0)
Minimal	1	0
CT INFILTRATION, MONONUCLEAR CELL	(1)	(0)
Minimal	1	0
CT INFILTRATION, EOSINOPHIL	(1)	(0)
Minimal	1	0
CT MINERALIZED MATERIAL	(1)	(0)
Minimal	1	0
**CD G-LT;**		
Examined	(0)	(3)
Within Normal Limits	0	1
CATHETER TRACK (CT)	0	2
CT PIGMENTED MACROPHAGES	(0)	(1)

Pathology - Intergroup Comparison of Histopathology Observations; Recovery
15-RS-288 - 40-Week Toxicity Stud of Recombinant-Methionyl Human Glial Cell Line-Derived Neurotrophic Factor (r-metHuGDNF) via Intermittent Bilateral Intraputamenal Convection-Enhanced Delivery in Rhesus Monkeys with a 12-Week Recovery Period

Observations: Neo-Plastic and Non Neo-Plastic

Removal Reason: Recovery Sacrifice

	----- MALES -----	
	MC 0 µg	MG 87.1 µg
Number of Animals on Study :	3	5
Number of Animals Completed:	(3)	(5)
CD G-LT; (continued)		
Minimal	0	1
CT FOREIGN BODY REACTION	(0)	(2)
Minimal	0	2
CT GLIOSIS/ASTROCYTOSIS	(0)	(1)
Mild	0	1
CT INFILTRATION, NEUTROPHIL	(0)	(1)
Mild	0	1
CT INFILTRATION, EOSINOPHIL	(0)	(1)
Minimal	0	1
CT INFILTRATION, MONONUCLEAR CELL	(0)	(1)
Minimal	0	1
CT PERIVASCULAR CUFFS; Mononuclear cell	(0)	(1)
Minimal	0	1
CT VACUOLATION, WHITE MATTER	(0)	(1)
Minimal	0	1
CT FIBROSIS	(0)	(1)
Minimal	0	1
CT VACUOLATED MACROPHAGES	(0)	(1)
Minimal	0	1
CT MINERALIZED MATERIAL	(0)	(1)
Minimal	0	1
GDNF IMMUNOSTAINING	(0)	(2)
Minimal	0	1
Mild	0	1
CD G-RT;		
Examined	(0)	(3)
Within Normal Limits	0	1
CATHETER TRACK (CT)	0	2
CT FOREIGN BODY REACTION	(0)	(2)
Minimal	0	1
Mild	0	1
CT PIGMENTED MACROPHAGES	(0)	(2)
Minimal	0	2
CT GLIOSIS/ASTROCYTOSIS	(0)	(2)
Minimal	0	2

Pathology - Intergroup Comparison of Histopathology Observations; Recovery
15-RS-288 - 40-Week Toxicity Stud of Recombinant-Methionyl Human Glial Cell Line-Derived Neurotrophic Factor (r-metHuGDNF) via Intermittent Bilateral Intraputamenal Convection-Enhanced Delivery in Rhesus Monkeys with a 12-Week Recovery Period

Observations: Neo-Plastic and Non Neo-Plastic

Removal Reason: Recovery Sacrifice

	MALES	
	MC 0 µg	MG 87.1 µg
Number of Animals on Study :	3	5
Number of Animals Completed:	(3)	(5)
CD G-RT; (continued)		
CT FIBROSIS	(0)	(1)
Moderate	0	1
CT INFILTRATION, NEUTROPHIL	(0)	(1)
Moderate	0	1
CT INFILTRATION, MONONUCLEAR CELL	(0)	(1)
Mild	0	1
CT VACUOLATED MACROPHAGES	(0)	(1)
Mild	0	1
CT VACUOLATION, WHITE MATTER	(0)	(1)
Mild	0	1
GDNF IMMUNOSTAINING	(0)	(2)
Minimal	0	1
Mild	0	1
CD/PUT G-LT;		
Examined	(3)	(2)
Within Normal Limits	1	1
CATHETER TRACK (CT)	2	1
CT PIGMENTED MACROPHAGES	(2)	(0)
Minimal	2	0
CT INFILTRATION, MONONUCLEAR CELL	(1)	(0)
Minimal	1	0
CT GLIOSIS/ASTROCYTOSIS	(1)	(0)
Minimal	1	0
CT FIBROSIS	(1)	(1)
Minimal	1	0
Mild	0	1
CT INFILTRATION, EOSINOPHIL	(1)	(0)
Minimal	1	0
CT FOREIGN BODY REACTION	(1)	(1)
Minimal	1	1
CT VACUOLATION, WHITE MATTER	(1)	(0)
Minimal	1	0
CT PERIVASCULAR CUFFS; Mononuclear cell	(1)	(0)
Minimal	1	0
CT MINERALIZED MATERIAL	(0)	(1)

Pathology - Intergroup Comparison of Histopathology Observations; Recovery
15-RS-288 - 40-Week Toxicity Stud of Recombinant-Methionyl Human Glial Cell Line-Derived Neurotrophic Factor (r-metHuGDNF) via Intermittent Bilateral Intraputamenal Convection-Enhanced Delivery in Rhesus Monkeys with a 12-Week Recovery Period

Observations: Neo-Plastic and Non Neo-Plastic

Removal Reason: Recovery Sacrifice

	MALES MC 0 µg	MALES MG 87.1 µg
Number of Animals on Study :	3	5
Number of Animals Completed:	(3)	(5)
**CD/PUT G-LT; (continued)**		
Minimal	0	1
GDNF IMMUNOSTAINING	(0)	(1)
Minimal	0	1
**CD/PUT G-RT;**		
Examined	(3)	(2)
Within Normal Limits	1	2
FIBROSIS; Meninges	(1)	(0)
Minimal	1	0
CATHETER TRACK (CT)	2	0
CT FOREIGN BODY REACTION	(1)	(0)
Minimal	1	0
CT INFILTRATION, MONONUCLEAR CELL	(1)	(0)
Minimal	1	0
CT PIGMENTED MACROPHAGES	(1)	(0)
Minimal	1	0
CT INFILTRATION, EOSINOPHIL	(1)	(0)
Minimal	1	0
**CD/PUT H-LT;**		
Examined	(3)	(5)
Within Normal Limits	1	1
CATHETER TRACK (CT)	2	4
CT FIBROSIS	(2)	(4)
Minimal	1	2
Mild	0	2
Moderate	1	0
CT GLIOSIS/ASTROCYTOSIS	(1)	(2)
Minimal	1	1
Mild	0	1
CT INFILTRATION, MONONUCLEAR CELL	(1)	(2)
Minimal	0	1
Mild	1	1
CT PIGMENTED MACROPHAGES	(1)	(4)
Minimal	1	4
CT INFILTRATION, EOSINOPHIL	(0)	(1)

Pathology - Intergroup Comparison of Histopathology Observations; Recovery
15-RS-288 - 40-Week Toxicity Stud of Recombinant-Methionyl Human Glial Cell Line-Derived Neurotrophic Factor (r-metHuGDNF) via Intermittent Bilateral Intraputamenal Convection-Enhanced Delivery in Rhesus Monkeys with a 12-Week Recovery Period

Observations: Neo-Plastic and Non Neo-Plastic

Removal Reason: Recovery Sacrifice

	MALES MC 0 µg	MALES MG 87.1 µg
Number of Animals on Study :	3	5
Number of Animals Completed:	(3)	(5)
**CD/PUT H-LT; (continued)**		
Mild	0	1
CT FOREIGN BODY REACTION	(2)	(4)
Minimal	2	3
Mild	0	1
INFILTRATION; Mononuclear cell; Meninges	(0)	(1)
Minimal	0	1
CT VACUOLATION, WHITE MATTER	(1)	(2)
Minimal	1	1
Moderate	0	1
FIBROSIS; Meninges	(0)	(1)
Minimal	0	1
CT VACUOLATION, GRAY MATTER	(0)	(1)
Minimal	0	1
CT INFILTRATION, NEUTROPHIL	(0)	(1)
Moderate	0	1
CT PERIVASCULAR CUFFS; Mixed	(0)	(1)
Minimal	0	1
CT MINERALIZED MATERIAL	(1)	(1)
Minimal	1	1
GDNF IMMUNOSTAINING	(0)	(4)
Minimal	0	2
Mild	0	2
**CD/PUT H-RT;**		
Examined	(3)	(5)
Within Normal Limits	0	0
CATHETER TRACK (CT)	3	5
CT FIBROSIS	(2)	(4)
Minimal	2	1
Mild	0	2
Moderate	0	1
CT GLIOSIS/ASTROCYTOSIS	(1)	(3)
Minimal	1	1
Mild	0	2
CT INFILTRATION, MONONUCLEAR CELL	(2)	(2)
Minimal	2	1

Pathology - Intergroup Comparison of Histopathology Observations; Recovery
15-RS-288 - 40-Week Toxicity Stud of Recombinant-Methionyl Human Glial Cell Line-Derived Neurotrophic Factor (r-metHuGDNF) via Intermittent Bilateral Intraputamenal Convection-Enhanced Delivery in Rhesus Monkeys with a 12-Week Recovery Period

Observations: Neo-Plastic and Non Neo-Plastic

Removal Reason: Recovery Sacrifice

	MALES MC 0 µg	MALES MG 87.1 µg
Number of Animals on Study :	3	5
Number of Animals Completed:	(3)	(5)
CD/PUT H-RT; (continued)		
Mild	0	1
CT INFILTRATION, NEUTROPHIL	(0)	(1)
Moderate	0	1
CT PIGMENTED MACROPHAGES	(0)	(5)
Minimal	0	4
Mild	0	1
CT VACUOLATED MACROPHAGES	(0)	(1)
Mild	0	1
CT PERIVASCULAR CUFFS; Mononuclear cell	(1)	(1)
Minimal	1	1
CT VACUOLATION, WHITE MATTER	(0)	(3)
Minimal	0	1
Mild	0	2
CT FOREIGN BODY REACTION	(1)	(4)
Minimal	1	4
CT INFILTRATION, EOSINOPHIL	(2)	(1)
Minimal	2	1
FIBROSIS; Meninges	(1)	(0)
Minimal	1	0
CT AXON SPHEROIDS	(0)	(1)
Minimal	0	1
DEGENERATION; Axon	(0)	(1)
Minimal	0	1
CT MINERALIZED MATERIAL	(2)	(3)
Minimal	2	3
GDNF IMMUNOSTAINING	(0)	(4)
Minimal	0	1
Mild	0	3
CD/PUT I-RT;		
Examined	(3)	(5)
Within Normal Limits	1	1
CATHETER TRACK (CT)	2	4
CT FOREIGN BODY REACTION	(2)	(2)
Minimal	2	2
CT PIGMENTED MACROPHAGES	(2)	(4)

Pathology - Intergroup Comparison of Histopathology Observations; Recovery
15-RS-288 - 40-Week Toxicity Stud of Recombinant-Methionyl Human Glial Cell Line-Derived Neurotrophic Factor (r-metHuGDNF) via Intermittent Bilateral Intraputamenal Convection-Enhanced Delivery in Rhesus Monkeys with a 12-Week Recovery Period

Observations: Neo-Plastic and Non Neo-Plastic

Removal Reason: Recovery Sacrifice

	MALES MC 0 µg	MALES MG 87.1 µg
Number of Animals on Study :	3	5
Number of Animals Completed:	(3)	(5)
CD/PUT I-RT; (continued)		
Minimal	2	3
Mild	0	1
CT INFILTRATION, MONONUCLEAR CELL	(1)	(1)
Minimal	1	0
Mild	0	1
CT FIBROSIS	(1)	(2)
Minimal	1	1
Moderate	0	1
CT VACUOLATION, WHITE MATTER	(0)	(2)
Minimal	0	1
Mild	0	1
CT GLIOSIS/ASTROCYTOSIS	(0)	(4)
Minimal	0	3
Mild	0	1
INFILTRATION; Mononuclear cell; Perivascular	(0)	(1)
Minimal	0	1
CT INFILTRATION, NEUTROPHIL	(0)	(1)
Moderate	0	1
CT VACUOLATED MACROPHAGES	(0)	(1)
Minimal	0	1
CT PERIVASCULAR CUFFS; Mononuclear cell	(0)	(1)
Minimal	0	1
CT INFILTRATION, EOSINOPHIL	(1)	(1)
Minimal	1	1
CT MINERALIZED MATERIAL	(2)	(2)
Minimal	2	2
GDNF IMMUNOSTAINING	(0)	(2)
Minimal	0	1
Mild	0	1
CD/PUT/AC I-LT;		
Examined	(3)	(5)
Within Normal Limits	0	1
CATHETER TRACK (CT)	3	3
CT FIBROSIS	(2)	(2)
Minimal	2	0

Pathology - Intergroup Comparison of Histopathology Observations; Recovery
15-RS-288 - 40-Week Toxicity Stud of Recombinant-Methionyl Human Glial Cell Line-Derived Neurotrophic Factor (r-metHuGDNF) via Intermittent Bilateral Intraputamenal Convection-Enhanced Delivery in Rhesus Monkeys with a 12-Week Recovery Period

Observations: Neo-Plastic and Non Neo-Plastic

Removal Reason: Recovery Sacrifice

	----- MALES -----	
	MC 0 µg	MG 87.1 µg
Number of Animals on Study :	3	5
Number of Animals Completed:	(3)	(5)
**CD/PUT/AC I-LT; (continued)**		
Mild	0	2
CT PERIVASCULAR CUFFS; Mixed	(0)	(1)
Minimal	0	1
CT PIGMENTED MACROPHAGES	(3)	(3)
Minimal	3	3
CT FOREIGN BODY REACTION	(3)	(3)
Minimal	3	3
CT VACUOLATION, WHITE MATTER	(1)	(2)
Minimal	1	1
Mild	0	1
CT INFILTRATION, MONONUCLEAR CELL	(1)	(2)
Minimal	1	1
Mild	0	1
CT INFILTRATION, EOSINOPHIL	(0)	(1)
Minimal	0	1
CT GLIOSIS/ASTROCYTOSIS	(2)	(2)
Minimal	2	1
Mild	0	1
CT INFILTRATION, NEUTROPHIL	(0)	(1)
Moderate	0	1
CT VACUOLATED MACROPHAGES	(0)	(1)
Mild	0	1
FIBROSIS; Meninges	(0)	(1)
Minimal	0	1
CT MINERALIZED MATERIAL	(2)	(1)
Minimal	2	1
GDNF IMMUNOSTAINING	(0)	(3)
Minimal	0	2
Mild	0	1
**CD/PUT/GP J-LT;**		
Examined	(3)	(5)
Within Normal Limits	1	3
CATHETER TRACK (CT)	1	2
CT FOREIGN BODY REACTION	(0)	(1)
Minimal	0	1

Pathology - Intergroup Comparison of Histopathology Observations; Recovery
15-RS-288 - 40-Week Toxicity Stud of Recombinant-Methionyl Human Glial Cell Line-Derived Neurotrophic Factor (r-metHuGDNF) via Intermittent Bilateral Intraputamenal Convection-Enhanced Delivery in Rhesus Monkeys with a 12-Week Recovery Period

Observations: Neo-Plastic and Non Neo-Plastic

Removal Reason: Recovery Sacrifice

	----- MALES -----	
	MC<br>0 µg	MG<br>87.1 µg
Number of Animals on Study :	3	5
Number of Animals Completed:	(3)	(5)
CD/PUT/GP J-LT; (continued)		
CT PIGMENTED MACROPHAGES	(0)	(2)
Minimal	0	2
CT FIBROSIS	(1)	(2)
Minimal	1	1
Mild	0	1
CT INFILTRATION, MONONUCLEAR CELL	(0)	(2)
Minimal	0	1
Mild	0	1
INFILTRATION; Mononuclear cell; Perivascular	(1)	(0)
Minimal	1	0
CT VACUOLATION, WHITE MATTER	(1)	(1)
Minimal	1	0
Mild	0	1
CT GLIOSIS/ASTROCYTOSIS	(1)	(1)
Minimal	1	1
CT INFILTRATION, NEUTROPHIL	(0)	(1)
Moderate	0	1
CT VACUOLATED MACROPHAGES	(1)	(1)
Minimal	1	1
CT PERIVASCULAR CUFFS; Mixed	(0)	(1)
Minimal	0	1
GDNF IMMUNOSTAINING	(0)	(2)
Mild	0	2
CD/PUT/GP J-RT;		
Examined	(3)	(5)
Within Normal Limits	2	2
CATHETER TRACK (CT)	1	3
CT GLIOSIS/ASTROCYTOSIS	(1)	(1)
Minimal	1	1
CT INFILTRATION, MONONUCLEAR CELL	(1)	(1)
Minimal	1	0
Mild	0	1
CT PERIVASCULAR CUFFS; Mononuclear cell	(0)	(1)
Minimal	0	1
CT VACUOLATION, WHITE MATTER	(0)	(1)

Pathology - Intergroup Comparison of Histopathology Observations; Recovery
15-RS-288 - 40-Week Toxicity Stud of Recombinant-Methionyl Human Glial Cell Line-Derived Neurotrophic Factor (r-metHuGDNF) via Intermittent Bilateral Intraputamenal Convection-Enhanced Delivery in Rhesus Monkeys with a 12-Week Recovery Period

Observations: Neo-Plastic and Non Neo-Plastic

Removal Reason: Recovery Sacrifice

	MALES	
	MC<br>0 µg	MG<br>87.1 µg
Number of Animals on Study :	3	5
Number of Animals Completed:	(3)	(5)
**CD/PUT/GP J-RT; (continued)**		
Mild	0	1
CT FIBROSIS	(1)	(3)
Mild	1	2
Moderate	0	1
CT PIGMENTED MACROPHAGES	(1)	(3)
Minimal	1	3
CT INFILTRATION, EOSINOPHIL	(1)	(0)
Minimal	1	0
CT INFILTRATION, NEUTROPHIL	(0)	(1)
Moderate	0	1
CT VACUOLATED MACROPHAGES	(0)	(1)
Mild	0	1
CT MINERALIZED MATERIAL	(0)	(1)
Minimal	0	1
GDNF IMMUNOSTAINING	(0)	(2)
Mild	0	2
**PUT/GP K-LT;**		
Examined	(2)	(4)
Within Normal Limits	2	2
INFILTRATION; Mononuclear cell; Perivascular	(0)	(1)
Minimal	0	1
AXON SPHEROIDS	(0)	(1)
Minimal	0	1
**PUT/GP K-RT;**		
Examined	(2)	(4)
Within Normal Limits	1	3
FIBROSIS; Meninges	(1)	(0)
Minimal	1	0
CATHETER TRACK (CT)	0	1
CT FIBROSIS	(0)	(1)
Mild	0	1
CT PERIVASCULAR CUFFS; Mononuclear cell	(0)	(1)
Minimal	0	1
CT INFILTRATION, MONONUCLEAR CELL	(0)	(1)

Pathology - Intergroup Comparison of Histopathology Observations; Recovery
15-RS-288 - 40-Week Toxicity Stud of Recombinant-Methionyl Human Glial Cell Line-Derived Neurotrophic Factor (r-metHuGDNF) via Intermittent Bilateral Intraputamenal Convection-Enhanced Delivery in Rhesus Monkeys with a 12-Week Recovery Period

Observations: Neo-Plastic and Non Neo-Plastic

Removal Reason: Recovery Sacrifice

	MALES	
	MC<br>0 µg	MG<br>87.1 µg
Number of Animals on Study :	3	5
Number of Animals Completed:	(3)	(5)
**PUT/GP K-RT; (continued)**		
Mild	0	1
CT PIGMENTED MACROPHAGES	(0)	(1)
Minimal	0	1
CT GLIOSIS/ASTROCYTOSIS	(0)	(1)
Mild	0	1
CT VACUOLATION, GRAY MATTER	(0)	(1)
Minimal	0	1
**SN M;**		
Examined	(0)	(2)
Within Normal Limits	0	1
**SN N;**		
Examined	(3)	(5)
Within Normal Limits	1	4
INFILTRATION; Mononuclear cell; Meninges	(1)	(0)
Minimal	1	0
FIBROSIS; Meninges	(1)	(0)
Minimal	1	0
**SN N-RT;**		
Examined	(1)	(0)
Within Normal Limits	1	0
**THAL/SN L;**		
Examined	(0)	(1)
Within Normal Limits	0	1
**THAL/SN L1-LT;**		
Examined	(0)	(1)
Within Normal Limits	0	1
**THAL/SN M;**		
Examined	(3)	(5)
Within Normal Limits	3	3
INFILTRATION; Mononuclear cell; Perivascular	(0)	(1)

Pathology - Intergroup Comparison of Histopathology Observations; Recovery
15-RS-288 - 40-Week Toxicity Stud of Recombinant-Methionyl Human Glial Cell Line-Derived Neurotrophic Factor (r-metHuGDNF) via Intermittent Bilateral Intraputamenal Convection-Enhanced Delivery in Rhesus Monkeys with a 12-Week Recovery Period

Observations: Neo-Plastic and Non Neo-Plastic

Removal Reason: Recovery Sacrifice

	MALES	
	MC 0 µg	MG 87.1 µg
Number of Animals on Study :	3	5
Number of Animals Completed:	(3)	(5)
**THAL/SN M; (continued)**		
Minimal	0	1
**THAL/STN K;**		
Examined	(0)	(1)
Within Normal Limits	0	1
**THAL/STN L;**		
Examined	(3)	(5)
Within Normal Limits	2	3
INFILTRATION; Mononuclear cell; Meninges	(1)	(0)
Minimal	1	0
AXON SPHEROIDS	(0)	(1)
Minimal	0	1
**THAL/STN L1-LT;**		
Examined	(2)	(3)
Within Normal Limits	1	2
INFILTRATION; Mononuclear cell	(1)	(0)
Minimal	1	0
INFILTRATION; Mononuclear cell; Meninges	(0)	(1)
Minimal	0	1
INFILTRATION; Neutrophilic	(1)	(0)
Minimal	1	0
PERIVASCULAR CUFFS, MIXED CELL	(1)	(0)
Minimal	1	0
**SPINAL CORD, CERVICAL;**		
Examined	(3)	(5)
Within Normal Limits	3	4
DEGENERATION; Axon	(0)	(1)
Minimal	0	1
**SPINAL CORD, THORACIC;**		
Examined	(3)	(5)
Within Normal Limits	3	4
DEGENERATION; Axon	(0)	(1)

Pathology - Intergroup Comparison of Histopathology Observations; Recovery
15-RS-288 - 40-Week Toxicity Stud of Recombinant-Methionyl Human Glial Cell Line-Derived Neurotrophic Factor (r-metHuGDNF) via Intermittent Bilateral Intraputamenal Convection-Enhanced Delivery in Rhesus Monkeys with a 12-Week Recovery Period

Observations: Neo-Plastic and Non Neo-Plastic

Removal Reason: Recovery Sacrifice

	MALES	
	MC<br>0 µg	MG<br>87.1 µg
Number of Animals on Study :	3	5
Number of Animals Completed:	(3)	(5)
**SPINAL CORD, THORACIC; (continued)**		
Minimal	0	1
**SPINAL CORD, LUMBAR;**		
Examined	(3)	(5)
Within Normal Limits	2	4
INFILTRATION; Mononuclear cell; Meninges	(1)	(0)
Minimal	1	0
DEGENERATION; Axon	(0)	(1)
Minimal	0	1
**DRG, CERVICAL;**		
Examined	(3)	(5)
Within Normal Limits	1	3
VACUOLATION; Ganglion cell	(2)	(2)
Minimal	2	2
MINERALIZATION	(1)	(0)
Minimal	1	0
**DRG, THORACIC;**		
Examined	(3)	(5)
Within Normal Limits	0	3
VACUOLATION; Ganglion cell	(3)	(1)
Minimal	3	1
MINERALIZATION	(1)	(0)
Minimal	1	0
DEGENERATION; Axon	(0)	(1)
Minimal	0	1
**DRG, LUMBAR;**		
Examined	(3)	(5)
Within Normal Limits	2	1
VACUOLATION; Ganglion cell	(1)	(4)
Minimal	1	4
**TRIGEMINAL GANGLIA;**		
Examined	(3)	(4)

Pathology - Intergroup Comparison of Histopathology Observations; Recovery
15-RS-288 - 40-Week Toxicity Stud of Recombinant-Methionyl Human Glial Cell Line-Derived Neurotrophic Factor (r-metHuGDNF) via Intermittent Bilateral Intraputamenal Convection-Enhanced Delivery in Rhesus Monkeys with a 12-Week Recovery Period

Observations: Neo-Plastic and Non Neo-Plastic

Removal Reason: Recovery Sacrifice

	----- MALES -----	
	MC	MG
	0 µg	87.1 µg
Number of Animals on Study :	3	5
Number of Animals Completed:	(3)	(5)
TRIGEMINAL GANGLIA; (continued)		
Within Normal Limits	3	4
Not Examined: INSUFFICIENT TISSUE TO EVALUATE FOLLOWING RECUT OR REHARVEST	0	1

Pathology - Intergroup Comparison of Histopathology Observations; Recovery
15-RS-288 - 40-Week Toxicity Stud of Recombinant-Methionyl Human Glial Cell Line-Derived Neurotrophic Factor (r-metHuGDNF) via Intermittent Bilateral Intraputamenal Convection-Enhanced Delivery in Rhesus Monkeys with a 12-Week Recovery Period

End Of Print

Table 1-1C. Intergroup Comparison of Histopathology Observations—Satellite

PTA005-05/00 Provantis Version 9.1 Date: 11/24/2015 9:44 Page: 1

Pathology - Intergroup Comparison of Histopathology Observations; Satellite
15-RS-288 - 40-Week Toxicity Stud of Recombinant-Methionyl Human Glial Cell Line-Derived Neurotrophic Factor (r-metHuGDNF) via Intermittent Bilateral Intraputamenal Convection-Enhanced Delivery in Rhesus Monkeys with a 12-Week Recovery Period

Observations: Neo-Plastic and Non Neo-Plastic

Removal Reason: Terminal Satellite Sacrifice

	- MALES SAT 87.1 µg
Number of Animals on Study :	4
Number of Animals Completed:	(4)
**BRAIN D;**	
Examined	(4)
Within Normal Limits	1
FIBROSIS; Meninges	(2)
Minimal	2
CATHETER TRACK (CT)	1
CT MINERALIZED MATERIAL	(1)
Minimal	1
**BS/PONS O-LT;**	
Examined	(4)
Within Normal Limits	4
**BS/PONS O-RT;**	
Examined	(4)
Within Normal Limits	4
**CB/MO T-CB;**	
Examined	(3)
Within Normal Limits	3
**CB/MO T-LT;**	
Examined	(3)
Within Normal Limits	3
**CB/MO T-RT;**	
Examined	(3)
Within Normal Limits	3
**CB/MO U-CB;**	
Examined	(1)
Within Normal Limits	1
**CB/MO U-LT;**	
Examined	(1)
Within Normal Limits	1

Pathology - Intergroup Comparison of Histopathology Observations; Satellite
15-RS-288 - 40-Week Toxicity Stud of Recombinant-Methionyl Human Glial Cell Line-Derived Neurotrophic Factor (r-metHuGDNF) via Intermittent Bilateral Intraputamenal Convection-Enhanced Delivery in Rhesus Monkeys with a 12-Week Recovery Period

Observations: Neo-Plastic and Non Neo-Plastic

Removal Reason: Terminal Satellite Sacrifice

	- MALES
	SAT 87.1 µg
Number of Animals on Study :	4
Number of Animals Completed:	(4)
**CB/MO U-RT;**	
Examined	(1)
Within Normal Limits	1
**CB R-CB;**	
Examined	(3)
Within Normal Limits	3
**CB S-CB;**	
Examined	(1)
Within Normal Limits	1
**CB V-CB;**	
Examined	(4)
Within Normal Limits	4
**CD F-LT;**	
Examined	(1)
Within Normal Limits	0
CATHETER TRACK (CT)	1
CT PIGMENTED MACROPHAGES	(1)
Minimal	1
CT FIBROSIS	(1)
Minimal	1
CT FOREIGN BODY REACTION	(1)
Minimal	1
GDNF IMMUNOSTAINING	(1)
Moderate	1
**CD F-RT;**	
Examined	(1)
Within Normal Limits	0
CATHETER TRACK (CT)	1
CT FOREIGN BODY REACTION	(1)
Minimal	1
CT FIBROSIS	(1)
Minimal	1

Pathology - Intergroup Comparison of Histopathology Observations; Satellite
15-RS-288 - 40-Week Toxicity Stud of Recombinant-Methionyl Human Glial Cell Line-Derived Neurotrophic Factor (r-metHuGDNF) via Intermittent Bilateral Intraputamenal Convection-Enhanced Delivery in Rhesus Monkeys with a 12-Week Recovery Period

Observations: Neo-Plastic and Non Neo-Plastic

Removal Reason: Terminal Satellite Sacrifice

	- MALES
	SAT
	87.1 µg
Number of Animals on Study :	4
Number of Animals Completed:	(4)
**CD F-RT; (continued)**	
GDNF IMMUNOSTAINING	(1)
Moderate	1
**CD G-LT;**	
Examined	(2)
Within Normal Limits	0
CATHETER TRACK (CT)	2
CT FOREIGN BODY REACTION	(2)
Minimal	2
CT INFILTRATION, MONONUCLEAR CELL	(2)
Minimal	2
CT VACUOLATION, WHITE MATTER	(1)
Minimal	1
GDNF IMMUNOSTAINING	(1)
Moderate	1
**CD G-RT;**	
Examined	(2)
Within Normal Limits	0
CATHETER TRACK (CT)	2
CT FOREIGN BODY REACTION	(1)
Minimal	1
CT PIGMENTED MACROPHAGES	(1)
Minimal	1
CT GLIOSIS/ASTROCYTOSIS	(2)
Minimal	2
CT VACUOLATED MACROPHAGES	(1)
Minimal	1
CT VACUOLATION, WHITE MATTER	(2)
Minimal	1
Mild	1
GDNF IMMUNOSTAINING	(2)
Moderate	2
**CD/PUT G-LT;**	
Examined	(2)

Pathology - Intergroup Comparison of Histopathology Observations; Satellite
15-RS-288 - 40-Week Toxicity Stud of Recombinant-Methionyl Human Glial Cell Line-Derived Neurotrophic Factor (r-metHuGDNF) via Intermittent Bilateral Intraputamenal Convection-Enhanced Delivery in Rhesus Monkeys with a 12-Week Recovery Period

Observations: Neo-Plastic and Non Neo-Plastic

Removal Reason: Terminal Satellite Sacrifice

	- MALES
	SAT<br>87.1 µg
Number of Animals on Study :	4
Number of Animals Completed:	(4)
**CD/PUT G-LT; (continued)**	
Within Normal Limits	0
CATHETER TRACK (CT)	2
CT PIGMENTED MACROPHAGES	(2)
Minimal	2
CT INFILTRATION, MONONUCLEAR CELL	(1)
Minimal	1
CT FIBROSIS	(1)
Minimal	1
CT FOREIGN BODY REACTION	(2)
Minimal	2
CT VACUOLATION, WHITE MATTER	(1)
Minimal	1
CT HEMORRHAGE	(1)
Minimal	1
CT MINERALIZED MATERIAL	(1)
Minimal	1
GDNF IMMUNOSTAINING	(2)
Moderate	2
**CD/PUT G-RT;**	
Examined	(2)
Within Normal Limits	0
CATHETER TRACK (CT)	2
CT FOREIGN BODY REACTION	(2)
Minimal	2
CT INFILTRATION, MONONUCLEAR CELL	(1)
Minimal	1
CT PIGMENTED MACROPHAGES	(1)
Minimal	1
CT VACUOLATION, WHITE MATTER	(1)
Minimal	1
CT INFILTRATION, EOSINOPHIL	(1)
Minimal	1
CT PERIVASCULAR CUFFS; Mixed	(1)
Minimal	1
CT MINERALIZED MATERIAL	(2)

Pathology - Intergroup Comparison of Histopathology Observations; Satellite
15-RS-288 - 40-Week Toxicity Stud of Recombinant-Methionyl Human Glial Cell Line-Derived Neurotrophic Factor (r-metHuGDNF) via Intermittent Bilateral Intraputamenal Convection-Enhanced Delivery in Rhesus Monkeys with a 12-Week Recovery Period

Observations: Neo-Plastic and Non Neo-Plastic

Removal Reason: Terminal Satellite Sacrifice

	- MALES
	SAT 87.1 µg
Number of Animals on Study :	4
Number of Animals Completed:	(4)
**CD/PUT G-RT; (continued)**	
Minimal	2
GDNF IMMUNOSTAINING	(1)
Moderate	1
**CD/PUT H-LT;**	
Examined	(4)
Within Normal Limits	0
CATHETER TRACK (CT)	4
CT FIBROSIS	(1)
Mild	1
CT GLIOSIS/ASTROCYTOSIS	(2)
Minimal	1
Mild	1
CT INFILTRATION, MONONUCLEAR CELL	(2)
Minimal	2
CT PIGMENTED MACROPHAGES	(2)
Minimal	1
Mild	1
CT INFILTRATION, EOSINOPHIL	(1)
Minimal	1
CT FOREIGN BODY REACTION	(3)
Minimal	2
Mild	1
FIBROSIS; Meninges	(1)
Minimal	1
CT VACUOLATION, GRAY MATTER	(1)
Minimal	1
CT MINERALIZED MATERIAL	(3)
Minimal	3
GDNF IMMUNOSTAINING	(4)
Moderate	4
**CD/PUT H-RT;**	
Examined	(4)
Within Normal Limits	0
CATHETER TRACK (CT)	4

Pathology - Intergroup Comparison of Histopathology Observations; Satellite
15-RS-288 - 40-Week Toxicity Stud of Recombinant-Methionyl Human Glial Cell Line-Derived Neurotrophic Factor (r-metHuGDNF) via Intermittent Bilateral Intraputamenal Convection-Enhanced Delivery in Rhesus Monkeys with a 12-Week Recovery Period

Observations: Neo-Plastic and Non Neo-Plastic

Removal Reason: Terminal Satellite Sacrifice

	- MALES
	SAT
	87.1 µg
Number of Animals on Study :	4
Number of Animals Completed:	(4)
CD/PUT H-RT; (continued)	
CT FIBROSIS	(3)
Minimal	2
Mild	1
CT GLIOSIS/ASTROCYTOSIS	(2)
Minimal	2
CT INFILTRATION, MONONUCLEAR CELL	(3)
Minimal	3
CT PIGMENTED MACROPHAGES	(3)
Minimal	3
CT VACUOLATED MACROPHAGES	(1)
Mild	1
CT VACUOLATION, WHITE MATTER	(3)
Minimal	3
CT FOREIGN BODY REACTION	(4)
Minimal	4
CT VACUOLATION, GRAY MATTER	(1)
Minimal	1
CT MINERALIZED MATERIAL	(3)
Minimal	3
GDNF IMMUNOSTAINING	(3)
Moderate	3
CD/PUT I-RT;	
Examined	(4)
Within Normal Limits	0
CATHETER TRACK (CT)	3
CT FOREIGN BODY REACTION	(3)
Minimal	3
CT PIGMENTED MACROPHAGES	(3)
Minimal	3
CT INFILTRATION, MONONUCLEAR CELL	(1)
Minimal	1
CT FIBROSIS	(3)
Minimal	3
CT GLIOSIS/ASTROCYTOSIS	(1)
Minimal	1

Pathology - Intergroup Comparison of Histopathology Observations; Satellite
15-RS-288 - 40-Week Toxicity Stud of Recombinant-Methionyl Human Glial Cell Line-Derived Neurotrophic Factor (r-metHuGDNF) via Intermittent Bilateral Intraputamenal Convection-Enhanced Delivery in Rhesus Monkeys with a 12-Week Recovery Period

Observations: Neo-Plastic and Non Neo-Plastic

Removal Reason: Terminal Satellite Sacrifice

	- MALES SAT 87.1 µg
Number of Animals on Study :	4
Number of Animals Completed:	(4)
**CD/PUT I-RT; (continued)**	
CT VACUOLATION, GRAY MATTER	(1)
Minimal	1
CT MINERALIZED MATERIAL	(1)
Minimal	1
GDNF IMMUNOSTAINING	(3)
Mild	1
Moderate	1
Marked	1
**CD/PUT/AC I-LT;**	
Examined	(4)
Within Normal Limits	0
CATHETER TRACK (CT)	4
CT FIBROSIS	(2)
Minimal	2
CT PIGMENTED MACROPHAGES	(3)
Minimal	3
CT FOREIGN BODY REACTION	(2)
Minimal	2
CT VACUOLATION, WHITE MATTER	(2)
Minimal	2
CT INFILTRATION, MONONUCLEAR CELL	(3)
Minimal	3
CT GLIOSIS/ASTROCYTOSIS	(1)
Minimal	1
CT MINERALIZED MATERIAL	(1)
Minimal	1
GDNF IMMUNOSTAINING	(4)
Moderate	4
**CD/PUT/GP J-LT;**	
Examined	(4)
Within Normal Limits	1
CATHETER TRACK (CT)	2
CT FOREIGN BODY REACTION	(2)
Minimal	2

Pathology - Intergroup Comparison of Histopathology Observations; Satellite
15-RS-288 - 40-Week Toxicity Stud of Recombinant-Methionyl Human Glial Cell Line-Derived Neurotrophic Factor (r-metHuGDNF) via Intermittent Bilateral Intraputamenal Convection-Enhanced Delivery in Rhesus Monkeys with a 12-Week Recovery Period

Observations: Neo-Plastic and Non Neo-Plastic

Removal Reason: Terminal Satellite Sacrifice

	- MALES SAT 87.1 µg
Number of Animals on Study :	4
Number of Animals Completed:	(4)
**CD/PUT/GP J-LT; (continued)**	
CT PIGMENTED MACROPHAGES	(1)
Minimal	1
CT FIBROSIS	(1)
Minimal	1
CT GLIOSIS/ASTROCYTOSIS	(1)
Minimal	1
CT MINERALIZED MATERIAL	(2)
Minimal	2
AXON SPHEROIDS	(1)
Mild	1
GDNF IMMUNOSTAINING	(2)
Moderate	2
**CD/PUT/GP J-RT;**	
Examined	(4)
Within Normal Limits	2
CATHETER TRACK (CT)	2
CT GLIOSIS/ASTROCYTOSIS	(1)
Minimal	1
CT FIBROSIS	(1)
Minimal	1
CT FOREIGN BODY REACTION	(2)
Minimal	1
Mild	1
CT PIGMENTED MACROPHAGES	(1)
Minimal	1
CT MINERALIZED MATERIAL	(2)
Minimal	2
GDNF IMMUNOSTAINING	(1)
Moderate	1
**PUT/GP K-LT;**	
Examined	(3)
Within Normal Limits	2
GDNF IMMUNOSTAINING	(1)
Mild	1

Pathology - Intergroup Comparison of Histopathology Observations; Satellite
15-RS-288 - 40-Week Toxicity Stud of Recombinant-Methionyl Human Glial Cell Line-Derived Neurotrophic Factor (r-metHuGDNF) via Intermittent Bilateral Intraputamenal Convection-Enhanced Delivery in Rhesus Monkeys with a 12-Week Recovery Period

Observations: Neo-Plastic and Non Neo-Plastic

Removal Reason: Terminal Satellite Sacrifice

	- MALES
	SAT 87.1 µg
Number of Animals on Study :	4
Number of Animals Completed:	(4)
**PUT/GP K-RT;**	
Examined	(3)
Within Normal Limits	2
CATHETER TRACK (CT)	1
CT FIBROSIS	(1)
Minimal	1
CT PIGMENTED MACROPHAGES	(1)
Minimal	1
GDNF IMMUNOSTAINING	(1)
Moderate	1
**SN M;**	
Examined	(1)
Within Normal Limits	1
**SN N;**	
Examined	(3)
Within Normal Limits	2
INFILTRATION; Mononuclear cell; Meninges	(1)
Minimal	1
**THAL/SN L;**	
Examined	(1)
Within Normal Limits	0
INFILTRATION; Mononuclear cell; Perivascular	(1)
Minimal	1
AXON SPHEROIDS	(1)
Minimal	1
**THAL/SN M;**	
Examined	(2)
Within Normal Limits	2
**THAL/SN M-LT;**	
Examined	(1)
Within Normal Limits	1

Pathology - Intergroup Comparison of Histopathology Observations; Satellite
15-RS-288 - 40-Week Toxicity Stud of Recombinant-Methionyl Human Glial Cell Line-Derived Neurotrophic Factor (r-metHuGDNF) via Intermittent Bilateral Intraputamenal Convection-Enhanced Delivery in Rhesus Monkeys with a 12-Week Recovery Period

Observations: Neo-Plastic and Non Neo-Plastic

Removal Reason: Terminal Satellite Sacrifice

	- MALES
	SAT 87.1 µg
Number of Animals on Study :	4
Number of Animals Completed:	(4)
**THAL/SN M-RT;**	
Examined	(1)
Within Normal Limits	1
**THAL/STN K-LT;**	
Examined	(1)
Within Normal Limits	0
AXON SPHEROIDS	(1)
Minimal	1
**THAL/STN K-RT;**	
Examined	(1)
Within Normal Limits	1
**THAL/STN L;**	
Examined	(1)
Within Normal Limits	1
**THAL/STN L-LT;**	
Examined	(2)
Within Normal Limits	2
**THAL/STN L-RT;**	
Examined	(2)
Within Normal Limits	2
**SPINAL CORD, CERVICAL;**	
Examined	(4)
Within Normal Limits	4
**SPINAL CORD, THORACIC;**	
Examined	(4)
Within Normal Limits	4
**SPINAL CORD, LUMBAR;**	
Examined	(4)
Within Normal Limits	4

Pathology - Intergroup Comparison of Histopathology Observations; Satellite
15-RS-288 - 40-Week Toxicity Stud of Recombinant-Methionyl Human Glial Cell Line-Derived Neurotrophic Factor (r-metHuGDNF) via Intermittent Bilateral Intraputamenal Convection-Enhanced Delivery in Rhesus Monkeys with a 12-Week Recovery Period

Observations: Neo-Plastic and Non Neo-Plastic

Removal Reason: Terminal Satellite Sacrifice

	- MALES
	SAT 87.1 µg
Number of Animals on Study :	4
Number of Animals Completed:	(4)
DRG, CERVICAL;	
Examined	(4)
Within Normal Limits	4
DRG, THORACIC;	
Examined	(4)
Within Normal Limits	4
DRG, LUMBAR;	
Examined	(4)
Within Normal Limits	4
TRIGEMINAL GANGLIA;	
Examined	(4)
Within Normal Limits	4

Pathology - Intergroup Comparison of Histopathology Observations; Satellite
15-RS-288 - 40-Week Toxicity Stud of Recombinant-Methionyl Human Glial Cell Line-Derived Neurotrophic Factor (r-metHuGDNF) via Intermittent Bilateral Intraputamenal Convection-Enhanced Delivery in Rhesus Monkeys with a 12-Week Recovery Period

---

End Of Print

Table 1-2. Individual Animal Data (Concise Edition)

Pathology - Individual Animal Data (Concise Edition)
15-RS-288 - 40-Week Toxicity Stud of Recombinant-Methionyl Human Glial Cell Line-Derived Neurotrophic Factor (r-metHuGDNF) via Intermittent Bilateral Intraputamenal Convection-Enhanced Delivery in Rhesus Monkeys with a 12-Week Recovery Period

---

Animal Ref.: V000909 Group: 1 Sex: Male Species: Monkey Strain: Rhesus

Test Material: r-metHuGDNF Dose: MC 0 µg Route: See Protocol Study Type: Regulated Repeat Dose
Date of Death : 04/24/2015 Study Day No. (Week): 284 (41) Mode of Death: Terminal Sacrifice
Date of Necropsy: 04/24/2015 ** NECROPSY COMPLETE **

** EXAMINATION COMPLETE **

Histo Pathology Observations:

BS/PONS P-LT;
FIBROSIS; Meninges; Focal; Minimal

BS/PONS P-RT;
FIBROSIS; Meninges; Multifocal; Mild

CB/MO T-CB;
LFB/PAS slide present.

CB/MO T-LT;
FIBROSIS; Meninges; Focal; Mild

CB R-CB;
LFB/PAS slide present.

CD F-LT;
FIBROSIS; Meninges; Focal; Minimal
CATHETER TRACK (CT)
CT VACUOLATED MACROPHAGES; Multifocal; Mild
CT PIGMENTED MACROPHAGES; Multifocal; Minimal
CT INFILTRATION, MONONUCLEAR CELL; Multifocal; Minimal
CT GLIOSIS/ASTROCYTOSIS; Mild
CT HEMORRHAGE; Focal; Minimal
CT AXON SPHEROIDS; Minimal

CD F-RT;
CATHETER TRACK (CT)
CT INFILTRATION, NEUTROPHIL; Focal; Mild
CT FOREIGN BODY REACTION; Focal; Mild
CT FIBROSIS; Minimal
CT HEMORRHAGE; Focal; Minimal

Animal Ref.: V000909 Group: 1 Sex: Male (continued)

Histo Pathology Observations:

CD F-RT (continued);
CT GLIOSIS/ASTROCYTOSIS; Mild
CT INFILTRATION, MONONUCLEAR CELL; Multifocal; Minimal
CT VACUOLATED MACROPHAGES; Multifocal; Mild
CT VACUOLATION, WHITE MATTER; Focal; Minimal

CD/PUT G-LT;
FIBROSIS; Meninges; Focal; Minimal
CATHETER TRACK (CT)
CT VACUOLATED MACROPHAGES; Multifocal; Mild
CT PIGMENTED MACROPHAGES; Multifocal; Minimal
CT INFILTRATION, MONONUCLEAR CELL; Multifocal; Minimal
CT GLIOSIS/ASTROCYTOSIS; Mild
CT FIBROSIS; Minimal

CD/PUT G-RT;
FIBROSIS; Meninges; Focal; Minimal: There is a foreign material in the focus of fibrosis, which is located in a sulcus.
CATHETER TRACK (CT)
CT FIBROSIS; Moderate
CT FOREIGN BODY REACTION; Focal; Minimal
CT GLIOSIS/ASTROCYTOSIS; Mild
CT INFILTRATION, MONONUCLEAR CELL; Multifocal; Mild
CT INFILTRATION, NEUTROPHIL; Focal; Mild
CT VACUOLATED MACROPHAGES; Multifocal; Mild
CT PIGMENTED MACROPHAGES; Multifocal; Minimal
CT VACUOLATION, WHITE MATTER; Multifocal; Minimal

CD/PUT H-LT;
CATHETER TRACK (CT)
CT FIBROSIS; Minimal
CT GLIOSIS/ASTROCYTOSIS; Minimal
CT INFILTRATION, MONONUCLEAR CELL; Multifocal; Minimal
CT PIGMENTED MACROPHAGES; Multifocal; Minimal
CT VACUOLATED MACROPHAGES; Multifocal; Mild

Pathology - Individual Animal Data (Concise Edition)
15-RS-288 - 40-Week Toxicity Stud of Recombinant-Methionyl Human Glial Cell Line-Derived Neurotrophic Factor (r-metHuGDNF) via Intermittent Bilateral Intraputamenal Convection-Enhanced Delivery in Rhesus Monkeys with a 12-Week Recovery Period

---

Animal Ref.: V000909 Group: 1 Sex: Male (continued)

Histo Pathology Observations:

CD/PUT H-RT;
- CATHETER TRACK (CT)
- CT FIBROSIS; Moderate
- CT GLIOSIS/ASTROCYTOSIS; Mild
- CT INFILTRATION, MONONUCLEAR CELL; Multifocal; Mild
- CT INFILTRATION, NEUTROPHIL; Focal; Moderate
- CT PIGMENTED MACROPHAGES; Multifocal; Minimal
- CT VACUOLATED MACROPHAGES; Multifocal; Mild
- CT PERIVASCULAR CUFFS; Mixed; Multifocal; Mild
- CT VACUOLATION, WHITE MATTER; Multifocal; Mild

CD/PUT/AC I-LT;
- CATHETER TRACK (CT): Recut slide shows a small amount of material resembling the fibrous capsule of a catheter track and there is mineralized material with a few cells along its border resembling the mineralized material observed in other catheter tracks. Therefore, although there is a hole in the tissue, there is evidence of a catheter track along one edge of the hole.
- CT MINERALIZED MATERIAL; Minimal

CD/PUT/GP J-RT;
- CATHETER TRACK (CT)
- CT GLIOSIS/ASTROCYTOSIS; Minimal
- CT INFILTRATION, MONONUCLEAR CELL; Multifocal; Minimal
- CT PERIVASCULAR CUFFS; Mixed; Multifocal; Mild
- CT VACUOLATION, WHITE MATTER; Multifocal; Mild
- CT VACUOLATION, GRAY MATTER; Focal; Mild

PUT/GP K-LT;
- LFB/PAS slide present.

PUT/GP K-RT;
- VACUOLATION; White matter; Multifocal; Minimal

THAL/SN M;
- LFB/PAS slide present.

Pathology - Individual Animal Data (Concise Edition)
15-RS-288 - 40-Week Toxicity Stud of Recombinant-Methionyl Human Glial Cell Line-Derived Neurotrophic Factor (r-metHuGDNF) via Intermittent Bilateral Intraputamenal Convection-Enhanced Delivery in Rhesus Monkeys with a 12-Week Recovery Period

Animal Ref.: V000909 Group: 1 Sex: Male (continued)

Histo Pathology Observations:

THAL/STN L;
LFB/PAS slides (2) present.
VACUOLATION; White matter; Focal; Minimal

THAL/STN L1-LT;
INFILTRATION; Meninges; Mononuclear cell; Focal; Minimal

The following tissues were within normal limits:

BRAIN D	CB/MO T-CB	CB/MO T-RT	CB R-CB	CB V-CB	CD/PUT/GP J-LT	PUT/GP K-LT
SN N	THAL/SN M	SPINAL CORD, CERVICAL		SPINAL CORD, THORACIC		SPINAL CORD, LUMBAR
DRG, CERVICAL	DRG, THORACIC	DRG, LUMBAR	TRIGEMINAL GANGLIA			

Pathology - Individual Animal Data (Concise Edition)
15-RS-288 - 40-Week Toxicity Stud of Recombinant-Methionyl Human Glial Cell Line-Derived Neurotrophic Factor (r-metHuGDNF) via Intermittent Bilateral Intraputamenal Convection-Enhanced Delivery in Rhesus Monkeys with a 12-Week Recovery Period

---

Animal Ref.: V002591 Group: 1 Sex: Male Species: Monkey Strain: Rhesus

Test Material: r-metHuGDNF Dose: MC 0 μg Route: See Protocol Study Type: Regulated Repeat Dose
Date of Death : 07/14/2015 Study Day No. (Week): 365 (53) Mode of Death: Recovery Sacrifice
Date of Necropsy: 07/14/2015 ** NECROPSY COMPLETE **

** EXAMINATION COMPLETE **

Histo Pathology Observations:

CB/MO U-CB;
There is a fixation or histologic artefact at the tip of the folia in one focus.

CB R-CB;
There is a fixation or histologic artefact at the tip of the folia in one focus.

CD/PUT H-RT;
CATHETER TRACK (CT)
CT FIBROSIS; Minimal
CT INFILTRATION, MONONUCLEAR CELL; Focal; Minimal
CT FOREIGN BODY REACTION; Multifocal; Minimal
CT INFILTRATION, EOSINOPHIL; Focal; Minimal
CT MINERALIZED MATERIAL; Minimal

CD/PUT I-RT;
CATHETER TRACK (CT)
CT FOREIGN BODY REACTION; Multifocal; Minimal
CT PIGMENTED MACROPHAGES; Focal; Minimal
CT INFILTRATION, EOSINOPHIL; Focal; Minimal
CT MINERALIZED MATERIAL; Minimal

CD/PUT/AC I-LT;
CATHETER TRACK (CT)
CT FIBROSIS; Minimal
CT PIGMENTED MACROPHAGES; Focal; Minimal
CT FOREIGN BODY REACTION; Multifocal; Minimal
CT GLIOSIS/ASTROCYTOSIS; Minimal
CT MINERALIZED MATERIAL; Minimal

Pathology - Individual Animal Data (Concise Edition)
15-RS-288 - 40-Week Toxicity Stud of Recombinant-Methionyl Human Glial Cell Line-Derived Neurotrophic Factor (r-metHuGDNF) via Intermittent Bilateral Intraputamenal Convection-Enhanced Delivery in Rhesus Monkeys with a 12-Week Recovery Period

---

Animal Ref.: V002591 Group: 1 Sex: Male (continued)

Histo Pathology Observations:

CD/PUT/GP J-LT;
INFILTRATION; Perivascular; Mononuclear cell; Focal; Minimal

CD/PUT/GP J-RT;
CATHETER TRACK (CT)
CT GLIOSIS/ASTROCYTOSIS; Minimal
CT INFILTRATION, MONONUCLEAR CELL; Focal; Minimal
CT FIBROSIS; Mild
CT PIGMENTED MACROPHAGES; Multifocal; Minimal
CT INFILTRATION, EOSINOPHIL; Focal; Minimal

PUT/GP K-RT;
LFB/PAS slide present.

SN N;
LFB/PAS slide present.
FIBROSIS; Meninges; Focal; Minimal

THAL/SN M;
LFB/PAS slide present.

THAL/STN L;
LFB/PAS slide present.
INFILTRATION; Meninges; Mononuclear cell; Focal; Minimal

THAL/STN L1-LT;
INFILTRATION; Mononuclear cell; Focal; Minimal
INFILTRATION; Neutrophilic; Focal; Minimal
PERIVASCULAR CUFFS, MIXED CELL; Multifocal; Minimal

SPINAL CORD, LUMBAR;
INFILTRATION; Meninges; Mononuclear cell; Multifocal; Minimal

DRG, THORACIC;
VACUOLATION; Ganglion cell; Minimal

Pathology - Individual Animal Data (Concise Edition)
15-RS-288 - 40-Week Toxicity Stud of Recombinant-Methionyl Human Glial Cell Line-Derived Neurotrophic Factor (r-metHuGDNF) via Intermittent Bilateral Intraputamenal Convection-Enhanced Delivery in Rhesus Monkeys with a 12-Week Recovery Period

---

Animal Ref.: V002591 Group: 1 Sex: Male (continued)

Histo Pathology Observations:

DRG, LUMBAR;
VACUOLATION; Ganglion cell; Minimal

The following tissues were within normal limits:

BRAIN D	BS/PONS O-LT	BS/PONS O-RT	CB/MO U-CB	CB/MO U-LT	CB/MO U-RT	CB R-CB
CB V-CB	CD/PUT G-LT	CD/PUT G-RT	CD/PUT H-LT	PUT/GP K-LT	PUT/GP K-RT	THAL/SN M
SPINAL CORD, CERVICAL		SPINAL CORD, THORACIC		DRG, CERVICAL	TRIGEMINAL GANGLIA	

Pathology - Individual Animal Data (Concise Edition)
15-RS-288 - 40-Week Toxicity Stud of Recombinant-Methionyl Human Glial Cell
Line-Derived Neurotrophic Factor (r-metHuGDNF) via Intermittent Bilateral
Intraputamenal Convection-Enhanced Delivery in Rhesus Monkeys with a 12-Week
Recovery Period

Animal Ref.: V002597 Group: 1 Sex: Male Species: Monkey Strain: Rhesus

Test Material: r-metHuGDNF Dose: MC 0 µg Route: See Protocol Study Type: Regulated Repeat Dose
Date of Death : 07/14/2015 Study Day No. (Week): 365 (53) Mode of Death: Recovery Sacrifice
Date of Necropsy: 07/14/2015 ** NECROPSY COMPLETE **

** EXAMINATION COMPLETE **

Histo Pathology Observations:

BRAIN D;
FIBROSIS; Meninges; Focal; Minimal
INFILTRATION; Meninges; Mononuclear cell; Multifocal; Minimal

CD F-LT;
CATHETER TRACK (CT)
CT INFILTRATION, MONONUCLEAR CELL; Multifocal; Minimal
CT INFILTRATION, EOSINOPHIL; Multifocal; Minimal
CT FOREIGN BODY REACTION; Multifocal; Minimal
CT PERIVASCULAR CUFFS; Mononuclear cell; Focal; Minimal

CD F-RT;
CATHETER TRACK (CT)
CT FOREIGN BODY REACTION; Multifocal; Minimal
CT INFILTRATION, MONONUCLEAR CELL; Multifocal; Minimal
CT INFILTRATION, EOSINOPHIL; Multifocal; Minimal
CT MINERALIZED MATERIAL; Minimal

CD/PUT G-LT;
CATHETER TRACK (CT)
CT PIGMENTED MACROPHAGES; Multifocal; Minimal
CT INFILTRATION, MONONUCLEAR CELL; Multifocal; Minimal
CT GLIOSIS/ASTROCYTOSIS; Minimal
CT FIBROSIS; Minimal
CT INFILTRATION, EOSINOPHIL; Multifocal; Minimal
CT FOREIGN BODY REACTION; Multifocal; Minimal
CT VACUOLATION, WHITE MATTER; Focal; Minimal
CT PERIVASCULAR CUFFS; Mononuclear cell; Multifocal; Minimal

CD/PUT G-RT;
FIBROSIS; Meninges; Focal; Minimal

Animal Ref.: V002597 Group: 1 Sex: Male (continued)

Histo Pathology Observations:

CD/PUT G-RT (continued);
CATHETER TRACK (CT)
CT FOREIGN BODY REACTION; Multifocal; Minimal
CT INFILTRATION, MONONUCLEAR CELL; Multifocal; Minimal
CT INFILTRATION, EOSINOPHIL; Multifocal; Minimal

CD/PUT H-LT;
CATHETER TRACK (CT)
CT FIBROSIS; Moderate
CT GLIOSIS/ASTROCYTOSIS; Minimal
CT INFILTRATION, MONONUCLEAR CELL; Multifocal; Mild
CT FOREIGN BODY REACTION; Multifocal; Minimal
CT VACUOLATION, WHITE MATTER; Multifocal; Minimal
CT MINERALIZED MATERIAL; Minimal

CD/PUT H-RT;
FIBROSIS; Meninges; Multifocal; Minimal
CATHETER TRACK (CT)
CT FIBROSIS; Minimal
CT GLIOSIS/ASTROCYTOSIS; Minimal
CT INFILTRATION, MONONUCLEAR CELL; Multifocal; Minimal
CT PERIVASCULAR CUFFS; Mononuclear cell; Multifocal; Minimal
CT INFILTRATION, EOSINOPHIL; Multifocal; Minimal

CD/PUT/AC I-LT;
CATHETER TRACK (CT)
CT FIBROSIS; Minimal
CT PIGMENTED MACROPHAGES; Multifocal; Minimal
CT FOREIGN BODY REACTION; Focal; Minimal
CT INFILTRATION, MONONUCLEAR CELL; Focal; Minimal
CT MINERALIZED MATERIAL; Minimal

CD/PUT/GP J-LT;
LFB/PAS slide present.

Pathology - Individual Animal Data (Concise Edition)
15-RS-288 - 40-Week Toxicity Stud of Recombinant-Methionyl Human Glial Cell Line-Derived Neurotrophic Factor (r-metHuGDNF) via Intermittent Bilateral Intraputamenal Convection-Enhanced Delivery in Rhesus Monkeys with a 12-Week Recovery Period

---

Animal Ref.: V002597 Group: 1 Sex: Male (continued)

Histo Pathology Observations:

SN N;
INFILTRATION; Meninges; Mononuclear cell; Focal; Minimal

THAL/SN M;
LFB/PAS slide present.

THAL/STN L;
LFB/PAS slides (2) present.

DRG, CERVICAL;
VACUOLATION; Ganglion cell; Minimal

DRG, THORACIC;
VACUOLATION; Ganglion cell; Minimal

The following tissues were within normal limits:

BS/PONS O-LT	BS/PONS O-RT	CB/MO U-CB	CB/MO U-LT	CB/MO U-RT	CB R-CB	CB V-CB
CD/PUT I-RT	CD/PUT/GP J-LT	CD/PUT/GP J-RT	SN N-RT	THAL/SN M	THAL/STN L	SPINAL CORD, CERVICAL
SPINAL CORD, THORACIC		SPINAL CORD, LUMBAR	DRG, LUMBAR	TRIGEMINAL GANGLIA		

Pathology - Individual Animal Data (Concise Edition)
15-RS-288 - 40-Week Toxicity Stud of Recombinant-Methionyl Human Glial Cell Line-Derived Neurotrophic Factor (r-metHuGDNF) via Intermittent Bilateral Intraputamenal Convection-Enhanced Delivery in Rhesus Monkeys with a 12-Week Recovery Period

---

Animal Ref.: V002604 Group: 1 Sex: Male Species: Monkey Strain: Rhesus

Test Material: r-metHuGDNF Dose: MC 0 µg Route: See Protocol Study Type: Regulated Repeat Dose
Date of Death : 04/24/2015 Study Day No. (Week): 284 (41) Mode of Death: Terminal Sacrifice
Date of Necropsy: 04/24/2015 ** NECROPSY COMPLETE **

** EXAMINATION COMPLETE **

Histo Pathology Observations:

BS/PONS P-LT;
FIBROSIS; Meninges; Focal; Minimal

CB/MO T-CB;
LFB slide present.

CB R-CB;
LFB slide present.

CD/PUT/AC I-LT;
CATHETER TRACK (CT)
CT FIBROSIS; Mild
CT PERIVASCULAR CUFFS; Mixed; Multifocal; Minimal
CT PIGMENTED MACROPHAGES; Multifocal; Minimal
CT FOREIGN BODY REACTION; Focal; Minimal
CT VACUOLATION, WHITE MATTER; Focal; Minimal
CT INFILTRATION, MONONUCLEAR CELL; Multifocal; Minimal
CT INFILTRATION, EOSINOPHIL; Multifocal; Minimal
CT GLIOSIS/ASTROCYTOSIS; Minimal

PUT/GP K-LT;
The area that appears to be either an artefact hole due to a section that is not flat/complete or a potential catheter track does not appear on the recut slilde and therefore is considered an artefact.

PUT/GP K-RT;
LFB/PAS slide present.

THAL/SN M;
LFB/PAS slide present.

Pathology - Individual Animal Data (Concise Edition)
15-RS-288 - 40-Week Toxicity Stud of Recombinant-Methionyl Human Glial Cell
Line-Derived Neurotrophic Factor (r-metHuGDNF) via Intermittent Bilateral
Intraputamenal Convection-Enhanced Delivery in Rhesus Monkeys with a 12-Week
Recovery Period

Animal Ref.: V002604 Group: 1 Sex: Male (continued)

Histo Pathology Observations:

THAL/STN L;
LFB/PAS slides (2) present.
VACUOLATION; White matter; Bilateral; Minimal

TRIGEMINAL GANGLIA;
MINERALIZATION; Minimal

The following tissues were within normal limits:

BRAIN D	BS/PONS P-RT	CB/MO T-CB	CB/MO T-LT	CB/MO T-RT	CB R-CB	CB V-CB
CD F-LT	CD F-RT	CD/PUT G-LT	CD/PUT G-RT	CD/PUT H-LT	CD/PUT H-RT	CD/PUT/GP J-LT
CD/PUT/GP J-RT	PUT/GP K-LT	PUT/GP K-RT	SN N	THAL/SN M	SPINAL CORD, CERVICAL	
SPINAL CORD, THORACIC		SPINAL CORD, LUMBAR	DRG, CERVICAL	DRG, THORACIC	DRG, LUMBAR	

Pathology - Individual Animal Data (Concise Edition)
15-RS-288 - 40-Week Toxicity Stud of Recombinant-Methionyl Human Glial Cell
Line-Derived Neurotrophic Factor (r-metHuGDNF) via Intermittent Bilateral
Intraputamenal Convection-Enhanced Delivery in Rhesus Monkeys with a 12-Week
Recovery Period

Animal Ref.: V002606 Group: 1 Sex: Male Species: Monkey Strain: Rhesus

Test Material: r-metHuGDNF Dose: MC 0 µg Route: See Protocol Study Type: Regulated Repeat Dose
Date of Death : 04/25/2015 Study Day No. (Week): 285 (41) Mode of Death: Terminal Sacrifice
Date of Necropsy: 04/25/2015 ** NECROPSY COMPLETE **

** EXAMINATION COMPLETE **

Histo Pathology Observations:

CB/MO T-CB;
LFB/PAS slide present.

CB R-CB;
LFB/PAS slide present.

CD/PUT G-LT;
CATHETER TRACK (CT)
CT MINERALIZED MATERIAL; Mild

CD/PUT G-RT;
CATHETER TRACK (CT)
CT FOREIGN BODY REACTION; Focal; Minimal

CD/PUT H-LT;
CATHETER TRACK (CT)
CT FIBROSIS; Minimal
CT GLIOSIS/ASTROCYTOSIS; Minimal
CT PIGMENTED MACROPHAGES; Focal; Minimal

CD/PUT H-RT;
CATHETER TRACK (CT)
CT FIBROSIS; Minimal
CT MINERALIZED MATERIAL; Minimal

CD/PUT/AC I-LT;
CATHETER TRACK (CT)
CT FIBROSIS; Minimal
CT PIGMENTED MACROPHAGES; Multifocal; Minimal
CT VACUOLATION, WHITE MATTER; Focal; Minimal
CT INFILTRATION, MONONUCLEAR CELL; Focal; Minimal

Animal Ref.: V002606 Group: 1 Sex: Male (continued)

Histo Pathology Observations:

CD/PUT/AC I-LT (continued);
CT INFILTRATION, EOSINOPHIL; Multifocal; Minimal

CD/PUT/GP J-RT;
CATHETER TRACK (CT)
CT FIBROSIS; Mild
CT FOREIGN BODY REACTION; Focal; Minimal

PUT/GP K-LT;
LFB/PAS slide present.

SN N;
LFB slide present. There is an artefact due to an incomplete/not flat secti-
on that creates an empty space, but there is no evidence on the LFB/PAS
slide or the recut H&E that it is a catheter track. There is also a LFB
recut.

THAL/SN M;
LFB/PAS slide present and recut LFB/PAS present.

THAL/STN L;
LFB/PAS slide present. There is an artefact due to an incomplete/not flat
section that creates an empty space, but there is no evidence on the LFB/PAS
slide or the recut that it is a catheter track.

DRG, THORACIC;
VACUOLATION; Ganglion cell; Minimal

The following tissues were within normal limits:

BRAIN D	BS/PONS P-LT	BS/PONS P-RT	CB/MO T-CB	CB/MO T-LT	CB/MO T-RT	CB R-CB
CB V-CB	CD F-LT	CD F-RT	CD/PUT/GP J-LT	PUT/GP K-LT	PUT/GP K-RT	SN N
THAL/SN M	THAL/STN L	SPINAL CORD, CERVICAL		SPINAL CORD, THORACIC		SPINAL CORD, LUMBAR
DRG, CERVICAL	DRG, LUMBAR	TRIGEMINAL GANGLIA				

Pathology - Individual Animal Data (Concise Edition)
15-RS-288 - 40-Week Toxicity Stud of Recombinant-Methionyl Human Glial Cell Line-Derived Neurotrophic Factor (r-metHuGDNF) via Intermittent Bilateral Intraputamenal Convection-Enhanced Delivery in Rhesus Monkeys with a 12-Week Recovery Period

---

Animal Ref.: V002613 Group: 1 Sex: Male Species: Monkey Strain: Rhesus

Test Material: r-metHuGDNF Dose: MC 0 µg Route: See Protocol Study Type: Regulated Repeat Dose
Date of Death : 07/15/2015 Study Day No. (Week): 366 (53) Mode of Death: Recovery Sacrifice
Date of Necropsy: 07/15/2015 ** NECROPSY COMPLETE **

** EXAMINATION COMPLETE **

Histo Pathology Observations:

CD/PUT G-LT;
CATHETER TRACK (CT)
CT PIGMENTED MACROPHAGES; Focal; Minimal

CD/PUT G-RT;
CATHETER TRACK (CT)
CT PIGMENTED MACROPHAGES; Focal; Minimal

CD/PUT H-LT;
CATHETER TRACK (CT)
CT FIBROSIS; Minimal
CT PIGMENTED MACROPHAGES; Focal; Minimal
CT FOREIGN BODY REACTION; Focal; Minimal

CD/PUT H-RT;
CATHETER TRACK (CT)
CT MINERALIZED MATERIAL; Minimal

CD/PUT I-RT;
CATHETER TRACK (CT)
CT FOREIGN BODY REACTION; Multifocal; Minimal
CT PIGMENTED MACROPHAGES; Multifocal; Minimal
CT INFILTRATION, MONONUCLEAR CELL; Focal; Minimal
CT FIBROSIS; Focal; Minimal
CT MINERALIZED MATERIAL; Minimal

CD/PUT/AC I-LT;
CATHETER TRACK (CT)
CT PIGMENTED MACROPHAGES; Focal; Minimal
CT FOREIGN BODY REACTION; Focal; Minimal
CT VACUOLATION, WHITE MATTER; Focal; Minimal

Animal Ref.: V002613 Group: 1 Sex: Male (continued)

Histo Pathology Observations:

CD/PUT/AC I-LT (continued);
CT GLIOSIS/ASTROCYTOSIS; Minimal

CD/PUT/GP J-LT;
CATHETER TRACK (CT)
CT FIBROSIS; Focal; Minimal
CT VACUOLATION, WHITE MATTER; Multifocal; Minimal
CT GLIOSIS/ASTROCYTOSIS; Multifocal; Minimal
CT VACUOLATED MACROPHAGES; Focal; Minimal

PUT/GP K-LT;
There is GDNF stain artifact near one edge of the section. This is not specific staining and appears to be a wash artifact.

PUT/GP K-RT;
LFB/PAS slide present.
FIBROSIS; Meninges; Multifocal; Minimal

SN N;
LFB/PAS slide present.

THAL/SN M;
LFB/PAS slide present.

THAL/STN L;
LFB/PAS slide present.

DRG, CERVICAL;
VACUOLATION; Ganglion cell; Minimal
MINERALIZATION; Minimal

DRG, THORACIC;
VACUOLATION; Ganglion cell; Minimal
MINERALIZATION; Minimal

Pathology - Individual Animal Data (Concise Edition)
15-RS-288 - 40-Week Toxicity Stud of Recombinant-Methionyl Human Glial Cell Line-Derived Neurotrophic Factor (r-metHuGDNF) via Intermittent Bilateral Intraputamenal Convection-Enhanced Delivery in Rhesus Monkeys with a 12-Week Recovery Period

Animal Ref.: V002613 Group: 1 Sex: Male (continued)

The following tissues were within normal limits:

BRAIN D	BS/PONS O-LT	BS/PONS O-RT	CB/MO U-CB	CB/MO U-LT	CB/MO U-RT	CB R-CB
CB V-CB	CD/PUT/GP J-RT	PUT/GP K-LT	SN N	THAL/SN M	THAL/STN L	THAL/STN L1-LT
SPINAL CORD, CERVICAL		SPINAL CORD, THORACIC		SPINAL CORD, LUMBAR	DRG, LUMBAR	TRIGEMINAL GANGLIA

Pathology - Individual Animal Data (Concise Edition)
15-RS-288 - 40-Week Toxicity Stud of Recombinant-Methionyl Human Glial Cell Line-Derived Neurotrophic Factor (r-metHuGDNF) via Intermittent Bilateral Intraputamenal Convection-Enhanced Delivery in Rhesus Monkeys with a 12-Week Recovery Period

---

Animal Ref.: V001935 Group: 2 Sex: Male Species: Monkey Strain: Rhesus

Test Material: r-metHuGDNF Dose: MG 87.1 µg Route: See Protocol Study Type: Regulated Repeat Dose
Date of Death : 07/16/2015 Study Day No. (Week): 366 (53) Mode of Death: Recovery Sacrifice
Date of Necropsy: 07/16/2015 ** NECROPSY COMPLETE **

** EXAMINATION COMPLETE **

Histo Pathology Observations:

CD/PUT H-RT;
- CATHETER TRACK (CT)
- CT FIBROSIS; Minimal
- CT GLIOSIS/ASTROCYTOSIS; Minimal
- CT PIGMENTED MACROPHAGES; Multifocal; Minimal
- CT FOREIGN BODY REACTION; Focal; Minimal
- CT INFILTRATION, EOSINOPHIL; Focal; Minimal
- GDNF IMMUNOSTAINING; Minimal

CD/PUT I-RT;
- INFILTRATION; Perivascular; Mononuclear cell; Focal; Minimal: The perivascular cuffs are located in the parenchyma away from (not adjacent to) the catheter track.
- CATHETER TRACK (CT)
- CT FOREIGN BODY REACTION; Focal; Minimal
- CT PIGMENTED MACROPHAGES; Focal; Minimal
- CT FIBROSIS; Multifocal; Minimal
- CT GLIOSIS/ASTROCYTOSIS; Multifocal; Minimal
- CT MINERALIZED MATERIAL; Minimal
- GDNF IMMUNOSTAINING; Mild

CD/PUT/AC I-LT;
- CATHETER TRACK (CT)
- CT PIGMENTED MACROPHAGES; Multifocal; Minimal
- CT FOREIGN BODY REACTION; Multifocal; Minimal
- CT INFILTRATION, MONONUCLEAR CELL; Multifocal; Minimal
- CT INFILTRATION, EOSINOPHIL; Multifocal; Minimal
- CT MINERALIZED MATERIAL; Minimal
- GDNF IMMUNOSTAINING; Mild

Animal Ref.: V001935 Group: 2 Sex: Male (continued)

Histo Pathology Observations:

CD/PUT/GP J-LT;
There is an area away from the catheter track with wide perivascular spaces
that were interpretted as an artefact of perfusion fixation.
CATHETER TRACK (CT)
CT FOREIGN BODY REACTION; Focal; Minimal
CT PIGMENTED MACROPHAGES; Focal; Minimal
CT FIBROSIS; Focal; Minimal
CT INFILTRATION, MONONUCLEAR CELL; Focal; Minimal
GDNF IMMUNOSTAINING; Mild

CD/PUT/GP J-RT;
CATHETER TRACK (CT)
CT FIBROSIS; Mild
CT PIGMENTED MACROPHAGES; Multifocal; Minimal
GDNF IMMUNOSTAINING; Mild

PUT/GP K-LT;
LFB/PAS slide present.
AXON SPHEROIDS; Focal; Minimal: Axon spheroids observed in H&E stained slide
and they fluoresce in FJC stained slide.

THAL/SN M;
LFB/PAS slide present.
INFILTRATION; Perivascular; Mononuclear cell; Focal; Minimal

THAL/STN L;
LFB/PAS slides (2) present.
AXON SPHEROIDS; Focal; Minimal: Axon spheroids observed in H&E stained slide
and they fluoresce in FJC stained slide.

DRG, THORACIC;
VACUOLATION; Ganglion cell; Minimal

DRG, LUMBAR;
VACUOLATION; Ganglion cell; Minimal

Pathology - Individual Animal Data (Concise Edition)
15-RS-288 - 40-Week Toxicity Stud of Recombinant-Methionyl Human Glial Cell Line-Derived Neurotrophic Factor (r-metHuGDNF) via Intermittent Bilateral Intraputamenal Convection-Enhanced Delivery in Rhesus Monkeys with a 12-Week Recovery Period

Animal Ref.: V001935 Group: 2 Sex: Male (continued)

The following tissues were within normal limits:

BRAIN D	BS/PONS O-LT	BS/PONS O-RT	CB/MO U-CB	CB/MO U-LT	CB/MO U-RT	CB R-CB
CB V-CB	CD G-LT	CD G-RT	CD/PUT H-LT	PUT/GP K-RT	SN N	THAL/STN L1-LT
SPINAL CORD, CERVICAL		SPINAL CORD, THORACIC		SPINAL CORD, LUMBAR	DRG, CERVICAL	TRIGEMINAL GANGLIA

Pathology - Individual Animal Data (Concise Edition)
15-RS-288 - 40-Week Toxicity Stud of Recombinant-Methionyl Human Glial Cell
Line-Derived Neurotrophic Factor (r-metHuGDNF) via Intermittent Bilateral
Intraputamenal Convection-Enhanced Delivery in Rhesus Monkeys with a 12-Week
Recovery Period

Animal Ref.: V001946 Group: 2 Sex: Male Species: Monkey Strain: Rhesus

Test Material: r-metHuGDNF Dose: MG 87.1 µg Route: See Protocol Study Type: Regulated Repeat Dose
Date of Death : 04/27/2015 Study Day No. (Week): 285 (41) Mode of Death: Terminal Sacrifice
Date of Necropsy: 04/27/2015 ** NECROPSY COMPLETE **

** EXAMINATION COMPLETE **

Histo Pathology Observations:

CB R-CB;
LFB slides (2) present.

CD F-RT;
CATHETER TRACK (CT)
CT MINERALIZED MATERIAL; Minimal

CD/PUT G-LT;
CATHETER TRACK (CT): The track is right at the surface of the cortex.
CT MINERALIZED MATERIAL; Mild: There is mineralized material in the catheter track and extending into the adjacent meninges.

CD/PUT G-RT;
CATHETER TRACK (CT): The catheter track is right at the surface of the cortex.
CT FOREIGN BODY REACTION; Focal; Minimal
CT PIGMENTED MACROPHAGES; Multifocal; Minimal
CT MINERALIZED MATERIAL; Minimal
GDNF IMMUNOSTAINING; Mild

CD/PUT H-LT;
CATHETER TRACK (CT)
CT INFILTRATION, MONONUCLEAR CELL; Multifocal; Minimal
CT PIGMENTED MACROPHAGES; Multifocal; Minimal
CT MINERALIZED MATERIAL; Minimal

CD/PUT H-RT;
CATHETER TRACK (CT)
CT INFILTRATION, MONONUCLEAR CELL; Multifocal; Minimal
CT PIGMENTED MACROPHAGES; Multifocal; Minimal
CT FOREIGN BODY REACTION; Focal; Minimal

Animal Ref.: V001946 Group: 2 Sex: Male (continued)

Histo Pathology Observations:

CD/PUT H-RT (continued);
CT MINERALIZED MATERIAL; Minimal
GDNF IMMUNOSTAINING; Moderate

CD/PUT/AC I-LT;
CATHETER TRACK (CT)
CT PIGMENTED MACROPHAGES; Multifocal; Minimal
CT FOREIGN BODY REACTION; Focal; Minimal
CT VACUOLATION, WHITE MATTER; Focal; Minimal
CT INFILTRATION, MONONUCLEAR CELL; Multifocal; Minimal
CT INFILTRATION, EOSINOPHIL; Focal; Minimal
CT GLIOSIS/ASTROCYTOSIS; Minimal
CT MINERALIZED MATERIAL; Minimal
GDNF IMMUNOSTAINING; Mild

CD/PUT/GP J-LT;
CATHETER TRACK (CT)
CT FOREIGN BODY REACTION; Focal; Minimal
CT PIGMENTED MACROPHAGES; Multifocal; Minimal
CT FIBROSIS; Focal; Mild
CT INFILTRATION, MONONUCLEAR CELL; Multifocal; Mild
CT HEMORRHAGE; Focal; Minimal
CT MINERALIZED MATERIAL; Minimal
GDNF IMMUNOSTAINING; Minimal

CD/PUT/GP J-RT;
CATHETER TRACK (CT): The track is right at the surface of the cortex.
CT INFILTRATION, MONONUCLEAR CELL; Multifocal; Minimal
CT FOREIGN BODY REACTION; Focal; Minimal
CT PIGMENTED MACROPHAGES; Multifocal; Minimal
GDNF IMMUNOSTAINING; Moderate

PUT/GP K-LT;
LFB/PAS slide present.

Pathology - Individual Animal Data (Concise Edition)
15-RS-288 - 40-Week Toxicity Stud of Recombinant-Methionyl Human Glial Cell Line-Derived Neurotrophic Factor (r-metHuGDNF) via Intermittent Bilateral Intraputamenal Convection-Enhanced Delivery in Rhesus Monkeys with a 12-Week Recovery Period

Animal Ref.: V001946 Group: 2 Sex: Male (continued)

Histo Pathology Observations:

PUT/GP K-RT;
GDNF IMMUNOSTAINING; Mild: A catheter track is not visible but there is focal GDNF immunostaining in small white matter tracks in the area of the globus pallidus.

THAL/SN M;
LFB/PAS slide present.

THAL/STN L;
LFB/PAS slides (2) present.

The following tissues were within normal limits:

BRAIN D	BS/PONS P-LT	BS/PONS P-RT	CB/MO T-CB	CB/MO T-LT	CB/MO T-RT	CB R-CB
CB U-CB	CD F-LT	PUT/GP K-LT	SN N	THAL/SN M	THAL/STN L	SPINAL CORD, CERVICAL
SPINAL CORD, THORACIC		SPINAL CORD, LUMBAR	DRG, CERVICAL	DRG, THORACIC	DRG, LUMBAR	TRIGEMINAL GANGLIA

Pathology - Individual Animal Data (Concise Edition)
15-RS-288 - 40-Week Toxicity Stud of Recombinant-Methionyl Human Glial Cell Line-Derived Neurotrophic Factor (r-metHuGDNF) via Intermittent Bilateral Intraputamenal Convection-Enhanced Delivery in Rhesus Monkeys with a 12-Week Recovery Period

---

Animal Ref.: V001954 Group: 2 Sex: Male Species: Monkey Strain: Rhesus

Test Material: r-metHuGDNF Dose: MG 87.1 µg Route: See Protocol Study Type: Regulated Repeat Dose
Date of Death : 04/25/2015 Study Day No. (Week): 283 (41) Mode of Death: Terminal Sacrifice
Date of Necropsy: 04/25/2015 ** NECROPSY COMPLETE **

** EXAMINATION COMPLETE **

Histo Pathology Observations:

BRAIN D;
The GDNF slide shows some brown staining in the meninges and along the surface. This stain is interpretted as non-specific "edge effect" because it includes nuclear staining and appears non-specific.
CT MINERALIZED MATERIAL; Focal; Minimal: The mineralized material is morphologically similar to the material observed in catheter tracks. The material is localized in to the mininges and a sulcus and the location and morphology suggest that is is associated with the start of catheter track that is just tangential to the section. Although the catheter track is not observed, the finding is entered as CT mineralized material because of the morphologic characteristics and the material may have been displaced during removal of the catheters at necropsy.

BS/PONS O-LT;
There is a focus in the brainstem where cells exhibit a punctate or granular brown staining in the GDNF-stained slide. However, the same brown, granular pigment is present in the H&E-stained slide. Therefore, this stain is interpretted as an endogenous pigment and not as specific GDNF immunostaining.

BS/PONS O-RT;
There is a focus in the brainstem where cells exhibit a punctate or granular brown staining in the GDNF-stained slide. However, the same brown, granular pigment is present in the H&E-stained slide. Therefore, this stain is interpretted as an endogenous pigment and not as specific GDNF immunostaining.

CB/MO T-CB;
LFB slide present.

CB R-CB;
LFB slide present.

Pathology - Individual Animal Data (Concise Edition)
15-RS-288 - 40-Week Toxicity Stud of Recombinant-Methionyl Human Glial Cell Line-Derived Neurotrophic Factor (r-metHuGDNF) via Intermittent Bilateral Intraputamenal Convection-Enhanced Delivery in Rhesus Monkeys with a 12-Week Recovery Period

---

Animal Ref.: V001954 Group: 2 Sex: Male (continued)

Histo Pathology Observations:

CD F-LT;
CATHETER TRACK (CT)
CT PIGMENTED MACROPHAGES; Multifocal; Minimal
CT GLIOSIS/ASTROCYTOSIS; Minimal
CT FIBROSIS; Focal; Minimal
CT INFILTRATION, EOSINOPHIL; Multifocal; Minimal
CT MINERALIZED MATERIAL; Minimal
GDNF IMMUNOSTAINING; Moderate

CD F-RT;
CATHETER TRACK (CT)
CT FIBROSIS; Minimal
CT GLIOSIS/ASTROCYTOSIS; Minimal
CT INFILTRATION, MONONUCLEAR CELL; Multifocal; Minimal
CT PIGMENTED MACROPHAGES; Multifocal; Minimal
CT INFILTRATION, EOSINOPHIL; Multifocal; Minimal
CT MINERALIZED MATERIAL; Minimal
GDNF IMMUNOSTAINING; Moderate

CD/PUT G-LT;
CATHETER TRACK (CT)
CT PIGMENTED MACROPHAGES; Multifocal; Minimal
CT GLIOSIS/ASTROCYTOSIS; Minimal
CT INFILTRATION, EOSINOPHIL; Multifocal; Minimal
CT FOREIGN BODY REACTION; Focal; Minimal
CT VACUOLATION, WHITE MATTER; Focal; Minimal
CT MINERALIZED MATERIAL; Minimal
GDNF IMMUNOSTAINING; Moderate

CD/PUT G-RT;
CATHETER TRACK (CT)
CT FIBROSIS; Minimal
CT FOREIGN BODY REACTION; Focal; Minimal
CT GLIOSIS/ASTROCYTOSIS; Minimal
CT INFILTRATION, MONONUCLEAR CELL; Multifocal; Minimal
CT PIGMENTED MACROPHAGES; Multifocal; Minimal

Animal Ref.: V001954 Group: 2 Sex: Male (continued)

Histo Pathology Observations:

CD/PUT G-RT (continued);
CT INFILTRATION, EOSINOPHIL; Multifocal; Minimal
CT MINERALIZED MATERIAL; Minimal
GDNF IMMUNOSTAINING; Moderate

CD/PUT H-LT;
CATHETER TRACK (CT)
CT GLIOSIS/ASTROCYTOSIS; Minimal
CT PIGMENTED MACROPHAGES; Multifocal; Mild
CT INFILTRATION, EOSINOPHIL; Multifocal; Minimal
CT FOREIGN BODY REACTION; Focal; Minimal
CT MINERALIZED MATERIAL; Minimal
GDNF IMMUNOSTAINING; Moderate

CD/PUT H-RT;
CATHETER TRACK (CT)
CT GLIOSIS/ASTROCYTOSIS; Minimal
CT PIGMENTED MACROPHAGES; Multifocal; Minimal
CT VACUOLATION, WHITE MATTER; Focal; Minimal
CT FOREIGN BODY REACTION; Focal; Minimal
CT INFILTRATION, EOSINOPHIL; Multifocal; Minimal
CT MINERALIZED MATERIAL; Minimal
GDNF IMMUNOSTAINING; Moderate

CD/PUT/AC I-LT;
INFILTRATION; Meninges; Mixed; Focal; Minimal
CATHETER TRACK (CT)
CT FIBROSIS; Mild
CT PERIVASCULAR CUFFS; Mixed; Multifocal; Mild
CT FOREIGN BODY REACTION; Focal; Minimal
CT INFILTRATION, EOSINOPHIL; Multifocal; Minimal
GDNF IMMUNOSTAINING; Moderate

CD/PUT/GP J-LT;
GDNF IMMUNOSTAINING; Moderate: There is a focus of moderate immunostaining that appears to be in the area of the globus pallidus. A catheter track is not apparent.

Pathology - Individual Animal Data (Concise Edition)
15-RS-288 - 40-Week Toxicity Stud of Recombinant-Methionyl Human Glial Cell Line-Derived Neurotrophic Factor (r-metHuGDNF) via Intermittent Bilateral Intraputamenal Convection-Enhanced Delivery in Rhesus Monkeys with a 12-Week Recovery Period

Animal Ref.: V001954 Group: 2 Sex: Male (continued)

Histo Pathology Observations:

PUT/GP K-RT;
LFB/PAS slide present.
AXON SPHEROIDS; Focal; Minimal: Axon spheroids observed in H&E stained slide and they fluoresce in FJC stained slide.

SN N;
There is a focus in the brainstem where cells exhibit a punctate or granular brown staining in the GDNF-stained slide. However, the same brown, granular pigment is present in the H&E-stained slide. Therefore, this stain is interpretted as an endogenous pigment and not as specific GDNF immunostaining.

THAL/SN M;
LFB/PAS slide present.
AXON SPHEROIDS; Focal; Minimal: Axon spheroids observed in H&E stained slide and they fluoresce in FJC stained slide.
GDNF IMMUNOSTAINING; Mild: There are two foci exhibiting GDNF immunoreactivity in the area of the thalamus although a catheter track is not visible.

THAL/STN L;
LFB/PAS slides (2) present.

DRG, THORACIC;
VACUOLATION; Ganglion cell; Mild

The following tissues were within normal limits:

BS/PONS O-LT	BS/PONS O-RT	CB/MO T-CB	CB/MO T-LT	CB/MO T-RT	CB R-CB	CB V-CB
CD/PUT/GP J-RT	PUT/GP K-LT	SN N	THAL/STN L	SPINAL CORD, CERVICAL		SPINAL CORD, THORACIC
SPINAL CORD, LUMBAR	DRG, CERVICAL	DRG, LUMBAR	TRIGEMINAL GANGLIA			

Pathology - Individual Animal Data (Concise Edition)
15-RS-288 - 40-Week Toxicity Stud of Recombinant-Methionyl Human Glial Cell Line-Derived Neurotrophic Factor (r-metHuGDNF) via Intermittent Bilateral Intraputamenal Convection-Enhanced Delivery in Rhesus Monkeys with a 12-Week Recovery Period

---

Animal Ref.: V002047 Group: 2 Sex: Male Species: Monkey Strain: Rhesus

Test Material: r-metHuGDNF Dose: MG 87.1 µg Route: See Protocol Study Type: Regulated Repeat Dose
Date of Death : 07/15/2015 Study Day No. (Week): 365 (53) Mode of Death: Recovery Sacrifice
Date of Necropsy: 07/15/2015 ** NECROPSY COMPLETE **

** EXAMINATION COMPLETE **

Histo Pathology Observations:

BS/PONS O-LT;

CB/MO U-RT;
FIBROSIS; Meninges; Focal; Mild

CD F-LT;
FIBROSIS; Meninges; Focal; Minimal
CATHETER TRACK (CT)
CT PIGMENTED MACROPHAGES; Focal; Minimal
CT INFILTRATION, MONONUCLEAR CELL; Focal; Minimal
CT FIBROSIS; Focal; Moderate
CT INFILTRATION, EOSINOPHIL; Focal; Minimal
CT FOREIGN BODY REACTION; Multifocal; Minimal
CT VACUOLATION, GRAY MATTER; Focal; Minimal
CT MINERALIZED MATERIAL; Minimal
GDNF IMMUNOSTAINING; Mild

CD F-RT;
FIBROSIS; Meninges; Focal; Minimal

CD/PUT H-LT;
CATHETER TRACK (CT)
CT FIBROSIS; Minimal
CT PIGMENTED MACROPHAGES; Multifocal; Minimal
CT FOREIGN BODY REACTION; Focal; Minimal
CT VACUOLATION, WHITE MATTER; Focal; Minimal
CT VACUOLATION, GRAY MATTER; Focal; Minimal
GDNF IMMUNOSTAINING; Mild

Animal Ref.: V002047 Group: 2 Sex: Male (continued)

Histo Pathology Observations:

CD/PUT H-RT;
CATHETER TRACK (CT)
CT FIBROSIS; Mild
CT GLIOSIS/ASTROCYTOSIS; Mild
CT PIGMENTED MACROPHAGES; Multifocal; Mild
CT PERIVASCULAR CUFFS; Mononuclear cell; Multifocal; Minimal: The cuffs primarily consist of pigmented macrophages.
CT VACUOLATION, WHITE MATTER; Multifocal; Mild
CT FOREIGN BODY REACTION; Focal; Minimal
CT MINERALIZED MATERIAL; Minimal
GDNF IMMUNOSTAINING; Mild

CD/PUT I-RT;
CATHETER TRACK (CT)
CT PIGMENTED MACROPHAGES; Focal; Minimal
CT GLIOSIS/ASTROCYTOSIS; Focal; Minimal

CD/PUT/AC I-LT;
FIBROSIS; Meninges; Focal; Minimal

SN M;
LFB/PAS slide present.

SN N;

THAL/SN L;
LFB/PAS slides (2) present.

THAL/SN M;

THAL/STN K;
LFB/PAS slide present.

Pathology - Individual Animal Data (Concise Edition)
15-RS-288 - 40-Week Toxicity Stud of Recombinant-Methionyl Human Glial Cell Line-Derived Neurotrophic Factor (r-metHuGDNF) via Intermittent Bilateral Intraputamenal Convection-Enhanced Delivery in Rhesus Monkeys with a 12-Week Recovery Period

---

Animal Ref.: V002047 Group: 2 Sex: Male (continued)

Histo Pathology Observations:

THAL/STN L;

The following tissues were within normal limits:

BRAIN D	BS/PONS N-LT	BS/PONS N-RT	BS/PONS O-RT	CB/MO U-CB	CB/MO U-LT	CB R-CB
CB V-CB	CD/PUT G-LT	CD/PUT G-RT	CD/PUT/GP J-LT	CD/PUT/GP J-RT	SN M	THAL/SN L
THAL/STN K	SPINAL CORD, CERVICAL		SPINAL CORD, THORACIC		SPINAL CORD, LUMBAR	DRG, CERVICAL
DRG, THORACIC	DRG, LUMBAR	TRIGEMINAL GANGLIA				

Pathology - Individual Animal Data (Concise Edition)
15-RS-288 - 40-Week Toxicity Stud of Recombinant-Methionyl Human Glial Cell Line-Derived Neurotrophic Factor (r-metHuGDNF) via Intermittent Bilateral Intraputamenal Convection-Enhanced Delivery in Rhesus Monkeys with a 12-Week Recovery Period

---

Animal Ref.: V002593 Group: 2 Sex: Male Species: Monkey Strain: Rhesus

Test Material: r-metHuGDNF Dose: MG 87.1 µg Route: See Protocol Study Type: Regulated Repeat Dose
Date of Death : 04/26/2015 Study Day No. (Week): 285 (41) Mode of Death: Terminal Sacrifice
Date of Necropsy: 04/26/2015 ** NECROPSY COMPLETE **

** EXAMINATION COMPLETE **

Histo Pathology Observations:

CB/MO T-CB;
LFB slide present. .

CB R-CB;
LFB slide present.

CD F-RT;
GDNF IMMUNOSTAINING; Moderate: There is an area of moderate GDNF immunoreactivity although the catheter track is not present in this section. The affected area lines a sulcus in the cortex.

CD/PUT G-LT;
CATHETER TRACK (CT)
CT FOREIGN BODY REACTION; Multifocal; Minimal
CT MINERALIZED MATERIAL; Minimal
GDNF IMMUNOSTAINING; Moderate: There is a focus of strong GDNF reactivity extending to a ventrical near to the catheter track.

CD/PUT G-RT;
CATHETER TRACK (CT)
CT FIBROSIS; Minimal
CT FOREIGN BODY REACTION; Multifocal; Minimal
CT INFILTRATION, MONONUCLEAR CELL; Multifocal; Minimal
CT PIGMENTED MACROPHAGES; Multifocal; Minimal
CT INFILTRATION, EOSINOPHIL; Multifocal; Minimal
GDNF IMMUNOSTAINING; Marked

CD/PUT H-LT;
CATHETER TRACK (CT)
CT FIBROSIS; Minimal
CT INFILTRATION, MONONUCLEAR CELL; Multifocal; Minimal

Animal Ref.: V002593 Group: 2 Sex: Male (continued)

Histo Pathology Observations:

CD/PUT H-LT (continued);
- CT PIGMENTED MACROPHAGES; Multifocal; Mild
- CT INFILTRATION, EOSINOPHIL; Multifocal; Mild: Small numbers of eosinophils extend into the neuropil peripheral to the catheter track.
- CT FOREIGN BODY REACTION; Multifocal; Minimal
- CT PERIVASCULAR CUFFS; Mixed; Multifocal; Minimal
- GDNF IMMUNOSTAINING; Marked

CD/PUT H-RT;
- CATHETER TRACK (CT)
- CT FIBROSIS; Mild
- CT INFILTRATION, MONONUCLEAR CELL; Multifocal; Minimal
- CT PIGMENTED MACROPHAGES; Multifocal; Mild
- CT PERIVASCULAR CUFFS; Mixed; Multifocal; Minimal
- CT INFILTRATION, EOSINOPHIL; Multifocal; Minimal
- GDNF IMMUNOSTAINING; Moderate

CD/PUT/AC I-LT;
- GDNF IMMUNOSTAINING; Moderate: There is GDNF immunostaining in gray and white matter tracks although a catheter track is not visible in this section.

CD/PUT/GP J-LT;
- GDNF IMMUNOSTAINING; Mild: There is focal staining, mainly in white matter tracks, although a catheter track is not visible.

CD/PUT/GP J-RT;
- GDNF IMMUNOSTAINING; Mild: There is focal staining, in white matter tracks, although a catheter track is not visible.

PUT/GP K-LT;
- LFB/PAS slide present.
- INFILTRATION; Meninges; Mononuclear cell; Focal; Minimal
- GDNF IMMUNOSTAINING; Mild: There is focal staining in white matter tracks as well as faint staining extending into gray matter, although a catheter track is not visible.

Pathology - Individual Animal Data (Concise Edition)
15-RS-288 - 40-Week Toxicity Stud of Recombinant-Methionyl Human Glial Cell Line-Derived Neurotrophic Factor (r-metHuGDNF) via Intermittent Bilateral Intraputamenal Convection-Enhanced Delivery in Rhesus Monkeys with a 12-Week Recovery Period

Animal Ref.: V002593 Group: 2 Sex: Male (continued)

Histo Pathology Observations:

PUT/GP K-RT;
INFILTRATION; Choroid plexus; Mononuclear cell; Focal; Minimal

THAL/SN M;
LFB/PAS slide present.

THAL/STN L;
LFB/PAS slides (2) present.
INFILTRATION; Meninges; Mononuclear cell; Focal; Minimal

DRG, THORACIC;
MINERALIZATION; Minimal

DRG, LUMBAR;
VACUOLATION; Ganglion cell; Minimal

The following tissues were within normal limits:

BRAIN D	BS/PONS O-LT	BS/PONS O-RT	CB/MO T-CB	CB/MO T-LT	CB/MO T-RT	CB R-CB
CB V-CB	CD F-LT	SN N	THAL/SN M	SPINAL CORD, CERVICAL		SPINAL CORD, THORACIC
SPINAL CORD, LUMBAR	DRG, CERVICAL	TRIGEMINAL GANGLIA				

Pathology - Individual Animal Data (Concise Edition)
15-RS-288 - 40-Week Toxicity Stud of Recombinant-Methionyl Human Glial Cell Line-Derived Neurotrophic Factor (r-metHuGDNF) via Intermittent Bilateral Intraputamenal Convection-Enhanced Delivery in Rhesus Monkeys with a 12-Week Recovery Period

---

Animal Ref.: V002608 Group: 2 Sex: Male Species: Monkey Strain: Rhesus

Test Material: r-metHuGDNF Dose: MG 87.1 µg Route: See Protocol Study Type: Regulated Repeat Dose
Date of Death : 07/16/2015 Study Day No. (Week): 365 (53) Mode of Death: Recovery Sacrifice
Date of Necropsy: 07/16/2015 ** NECROPSY COMPLETE **

** EXAMINATION COMPLETE **

Histo Pathology Observations:

CB R-CB;
There is a fixation or histologic artefact at the tip of the folia in one focus.

CD G-LT;
CATHETER TRACK (CT)
CT FOREIGN BODY REACTION; Multifocal; Minimal
CT INFILTRATION, NEUTROPHIL; Diffuse; Mild
CT INFILTRATION, EOSINOPHIL; Multifocal; Minimal
CT INFILTRATION, MONONUCLEAR CELL; Multifocal; Minimal
CT PERIVASCULAR CUFFS; Mononuclear cell; Multifocal; Minimal
CT VACUOLATION, WHITE MATTER; Focal; Minimal
CT FIBROSIS; Focal; Minimal
CT VACUOLATED MACROPHAGES; Multifocal; Minimal
GDNF IMMUNOSTAINING; Mild

CD G-RT;
CATHETER TRACK (CT)
CT FOREIGN BODY REACTION; Multifocal; Mild
CT PIGMENTED MACROPHAGES; Multifocal; Minimal
CT GLIOSIS/ASTROCYTOSIS; Minimal
CT FIBROSIS; Diffuse; Moderate
CT INFILTRATION, NEUTROPHIL; Diffuse; Moderate
CT INFILTRATION, MONONUCLEAR CELL; Multifocal; Mild
CT VACUOLATED MACROPHAGES; Multifocal; Mild
CT VACUOLATION, WHITE MATTER; Focal; Mild
GDNF IMMUNOSTAINING; Minimal

CD/PUT H-LT;
INFILTRATION; Meninges; Mononuclear cell; Focal; Minimal
CATHETER TRACK (CT)

Animal Ref.: V002608 Group: 2 Sex: Male (continued)

Histo Pathology Observations:

CD/PUT H-LT (continued);
CT FIBROSIS; Mild
CT GLIOSIS/ASTROCYTOSIS; Mild
CT INFILTRATION, MONONUCLEAR CELL; Multifocal; Mild
CT PIGMENTED MACROPHAGES; Multifocal; Minimal
CT INFILTRATION, EOSINOPHIL; Multifocal; Mild
CT FOREIGN BODY REACTION; Multifocal; Mild
CT VACUOLATION, WHITE MATTER; Multifocal; Moderate
CT INFILTRATION, NEUTROPHIL; Diffuse; Moderate
CT PERIVASCULAR CUFFS; Mixed; Multifocal; Minimal
GDNF IMMUNOSTAINING; Minimal

CD/PUT H-RT;
CATHETER TRACK (CT)
CT FIBROSIS; Moderate
CT GLIOSIS/ASTROCYTOSIS; Mild
CT INFILTRATION, MONONUCLEAR CELL; Multifocal; Mild
CT INFILTRATION, NEUTROPHIL; Diffuse; Moderate
CT PIGMENTED MACROPHAGES; Multifocal; Minimal
CT VACUOLATED MACROPHAGES; Multifocal; Mild
CT VACUOLATION, WHITE MATTER; Multifocal; Mild

CD/PUT I-RT;
CATHETER TRACK (CT)
CT PIGMENTED MACROPHAGES; Multifocal; Mild
CT INFILTRATION, MONONUCLEAR CELL; Multifocal; Mild
CT FIBROSIS; Focal; Moderate
CT VACUOLATION, WHITE MATTER; Multifocal; Mild
CT GLIOSIS/ASTROCYTOSIS; Multifocal; Mild
CT INFILTRATION, NEUTROPHIL; Diffuse; Moderate
CT VACUOLATED MACROPHAGES; Multifocal; Minimal
CT PERIVASCULAR CUFFS; Mononuclear cell; Multifocal; Minimal
CT INFILTRATION, EOSINOPHIL; Multifocal; Minimal

CD/PUT/AC I-LT;
CATHETER TRACK (CT)

Pathology - Individual Animal Data (Concise Edition)
15-RS-288 - 40-Week Toxicity Stud of Recombinant-Methionyl Human Glial Cell Line-Derived Neurotrophic Factor (r-metHuGDNF) via Intermittent Bilateral Intraputamenal Convection-Enhanced Delivery in Rhesus Monkeys with a 12-Week Recovery Period

---

Animal Ref.: V002608 Group: 2 Sex: Male (continued)

Histo Pathology Observations:

CD/PUT/AC I-LT (continued);
CT FIBROSIS; Mild
CT PERIVASCULAR CUFFS; Mixed; Multifocal; Minimal
CT PIGMENTED MACROPHAGES; Multifocal; Minimal
CT FOREIGN BODY REACTION; Multifocal; Minimal
CT VACUOLATION, WHITE MATTER; Multifocal; Mild
CT INFILTRATION, MONONUCLEAR CELL; Multifocal; Mild
CT GLIOSIS/ASTROCYTOSIS; Mild
CT INFILTRATION, NEUTROPHIL; Diffuse; Moderate
CT VACUOLATED MACROPHAGES; Multifocal; Mild
GDNF IMMUNOSTAINING; Minimal

CD/PUT/GP J-LT;
CATHETER TRACK (CT)
CT PIGMENTED MACROPHAGES; Multifocal; Minimal
CT FIBROSIS; Focal; Mild
CT INFILTRATION, MONONUCLEAR CELL; Multifocal; Mild
CT VACUOLATION, WHITE MATTER; Multifocal; Mild
CT GLIOSIS/ASTROCYTOSIS; Multifocal; Minimal
CT INFILTRATION, NEUTROPHIL; Diffuse; Moderate
CT VACUOLATED MACROPHAGES; Multifocal; Minimal
CT PERIVASCULAR CUFFS; Mixed; Focal; Minimal
GDNF IMMUNOSTAINING; Mild

CD/PUT/GP J-RT;
CATHETER TRACK (CT)
CT GLIOSIS/ASTROCYTOSIS; Minimal
CT INFILTRATION, MONONUCLEAR CELL; Multifocal; Mild
CT PERIVASCULAR CUFFS; Mononuclear cell; Multifocal; Minimal
CT VACUOLATION, WHITE MATTER; Multifocal; Mild
CT FIBROSIS; Moderate
CT PIGMENTED MACROPHAGES; Multifocal; Minimal
CT INFILTRATION, NEUTROPHIL; Diffuse; Moderate
CT VACUOLATED MACROPHAGES; Multifocal; Mild

Pathology - Individual Animal Data (Concise Edition)
15-RS-288 - 40-Week Toxicity Stud of Recombinant-Methionyl Human Glial Cell Line-Derived Neurotrophic Factor (r-metHuGDNF) via Intermittent Bilateral Intraputamenal Convection-Enhanced Delivery in Rhesus Monkeys with a 12-Week Recovery Period

---

Animal Ref.: V002608 Group: 2 Sex: Male (continued)

Histo Pathology Observations:

PUT/GP K-LT;
INFILTRATION; Perivascular; Mononuclear cell; Focal; Minimal

PUT/GP K-RT;
LFB/PAS slide present.
CATHETER TRACK (CT)
CT FIBROSIS; Focal; Mild
CT PERIVASCULAR CUFFS; Mononuclear cell; Multifocal; Minimal
CT INFILTRATION, MONONUCLEAR CELL; Multifocal; Mild
CT PIGMENTED MACROPHAGES; Multifocal; Minimal
CT GLIOSIS/ASTROCYTOSIS; Multifocal; Mild: Astrocytosis confirmed by GFAP stain. Enlarged astrocytes with diffuse cytoplasmic GFAP staining are present in the parenchyma surrounding the catheter track.
CT VACUOLATION, GRAY MATTER; Multifocal; Minimal

SN M;

SN N;
LFB/PAS slide present.

THAL/SN M;
LFB/PAS slide present.

THAL/STN L;
LFB/PAS slide present.

DRG, CERVICAL;
VACUOLATION; Ganglion cell; Minimal

DRG, THORACIC;
DEGENERATION; Axon; Multifocal; Minimal

DRG, LUMBAR;
VACUOLATION; Ganglion cell; Minimal

Pathology - Individual Animal Data (Concise Edition)
15-RS-288 - 40-Week Toxicity Stud of Recombinant-Methionyl Human Glial Cell Line-Derived Neurotrophic Factor (r-metHuGDNF) via Intermittent Bilateral Intraputamenal Convection-Enhanced Delivery in Rhesus Monkeys with a 12-Week Recovery Period

Animal Ref.: V002608 Group: 2 Sex: Male (continued)

The following tissues were within normal limits:

BRAIN D	BS/PONS O-LT	BS/PONS O-RT	CB/MO U-CB	CB/MO U-LT	CB/MO U-RT	CB R-CB
CB V-CB	SN N	THAL/SN M	THAL/STN L	THAL/STN L1-LT	SPINAL CORD, CERVICAL	
SPINAL CORD, THORACIC		SPINAL CORD, LUMBAR	TRIGEMINAL GANGLIA			

Pathology - Individual Animal Data (Concise Edition)
15-RS-288 - 40-Week Toxicity Stud of Recombinant-Methionyl Human Glial Cell Line-Derived Neurotrophic Factor (r-metHuGDNF) via Intermittent Bilateral Intraputamenal Convection-Enhanced Delivery in Rhesus Monkeys with a 12-Week Recovery Period

---

Animal Ref.: V002610 Group: 2 Sex: Male Species: Monkey Strain: Rhesus

Test Material: r-metHuGDNF Dose: MG 87.1 µg Route: See Protocol Study Type: Regulated Repeat Dose
Date of Death : 07/17/2015 Study Day No. (Week): 367 (53) Mode of Death: Recovery Sacrifice
Date of Necropsy: 07/17/2015 ** NECROPSY COMPLETE **

** EXAMINATION COMPLETE **

Histo Pathology Observations:

CB/MO T-CB;
INFILTRATION; Meninges; Mononuclear cell; Focal; Minimal
INFILTRATION; Perivascular; Mononuclear cell; Focal; Minimal

CB R-CB;
DEGENERATION; Axon; Focal; Minimal

CD G-LT;
CATHETER TRACK (CT)
CT PIGMENTED MACROPHAGES; Multifocal; Minimal
CT FOREIGN BODY REACTION; Focal; Minimal
CT GLIOSIS/ASTROCYTOSIS; Mild
CT MINERALIZED MATERIAL; Minimal
GDNF IMMUNOSTAINING; Minimal

CD G-RT;
CATHETER TRACK (CT)
CT FOREIGN BODY REACTION; Focal; Minimal
CT PIGMENTED MACROPHAGES; Multifocal; Minimal
CT GLIOSIS/ASTROCYTOSIS; Minimal
GDNF IMMUNOSTAINING; Mild

CD/PUT H-LT;
CATHETER TRACK (CT)
CT FIBROSIS; Mild
CT GLIOSIS/ASTROCYTOSIS; Minimal
CT INFILTRATION, MONONUCLEAR CELL; Multifocal; Minimal
CT PIGMENTED MACROPHAGES; Multifocal; Minimal
CT FOREIGN BODY REACTION; Multifocal; Minimal
GDNF IMMUNOSTAINING; Mild

Pathology - Individual Animal Data (Concise Edition)
15-RS-288 - 40-Week Toxicity Stud of Recombinant-Methionyl Human Glial Cell Line-Derived Neurotrophic Factor (r-metHuGDNF) via Intermittent Bilateral Intraputamenal Convection-Enhanced Delivery in Rhesus Monkeys with a 12-Week Recovery Period

Animal Ref.: V002610 Group: 2 Sex: Male (continued)

Histo Pathology Observations:

CD/PUT H-RT;
- CATHETER TRACK (CT)
- CT FIBROSIS; Mild
- CT INFILTRATION, MONONUCLEAR CELL; Multifocal; Minimal
- CT PIGMENTED MACROPHAGES; Multifocal; Minimal
- CT FOREIGN BODY REACTION; Multifocal; Minimal
- DEGENERATION; Axon; Focal; Minimal: The axonal degeneration is in the ventr-o-lateral medulla near the perivascular mononuclear cuff.
- CT MINERALIZED MATERIAL; Minimal
- GDNF IMMUNOSTAINING; Mild

PUT/GP K-LT;
- LFB/PAS slide present.

THAL/SN M;
- LFB/PAS slide present.

THAL/STN L;
- LFB/PAS slides (2) present.

SPINAL CORD, CERVICAL;
- DEGENERATION; Axon; Focal; Minimal

SPINAL CORD, THORACIC;
- DEGENERATION; Axon; Focal; Minimal

SPINAL CORD, LUMBAR;
- DEGENERATION; Axon; Focal; Minimal

DRG, CERVICAL;
- VACUOLATION; Ganglion cell; Minimal

DRG, LUMBAR;
- VACUOLATION; Ganglion cell; Minimal

Pathology - Individual Animal Data (Concise Edition)
15-RS-288 - 40-Week Toxicity Stud of Recombinant-Methionyl Human Glial Cell Line-Derived Neurotrophic Factor (r-metHuGDNF) via Intermittent Bilateral Intraputamenal Convection-Enhanced Delivery in Rhesus Monkeys with a 12-Week Recovery Period

Animal Ref.: V002610 Group: 2 Sex: Male (continued)

The following tissues were within normal limits:

BRAIN D	BS/PONS O-LT	BS/PONS O-RT	CB/MO T-LT	CB/MO T-RT	CB V-CB	CD/PUT I-RT
CD/PUT/AC I-LT	CD/PUT/GP J-LT	CD/PUT/GP J-RT	PUT/GP K-LT	PUT/GP K-RT	SN N	THAL/SN L1-LT
THAL/SN M	THAL/STN L	DRG, THORACIC	TRIGEMINAL GANGLIA			

Pathology - Individual Animal Data (Concise Edition)
15-RS-288 - 40-Week Toxicity Stud of Recombinant-Methionyl Human Glial Cell Line-Derived Neurotrophic Factor (r-metHuGDNF) via Intermittent Bilateral Intraputamenal Convection-Enhanced Delivery in Rhesus Monkeys with a 12-Week Recovery Period

---

Animal Ref.: V002611 Group: 2 Sex: Male Species: Monkey Strain: Rhesus

Test Material: r-metHuGDNF Dose: MG 87.1 µg Route: See Protocol Study Type: Regulated Repeat Dose
Date of Death : 04/26/2015 Study Day No. (Week): 285 (41) Mode of Death: Terminal Sacrifice
Date of Necropsy: 04/26/2015 ** NECROPSY COMPLETE **

** EXAMINATION COMPLETE **

Histo Pathology Observations:

BS/PONS P-LT;
FIBROSIS; Meninges; Focal; Minimal

BS/PONS P-RT;
FIBROSIS; Meninges; Focal; Minimal

CB/MO T-CB;
LFB slide present.

CB R-CB;
LFB slide present.

CD F-LT;
CATHETER TRACK (CT)
CT PIGMENTED MACROPHAGES; Multifocal; Moderate
CT GLIOSIS/ASTROCYTOSIS; Mild
GDNF IMMUNOSTAINING; Mild

CD F-RT;
GDNF IMMUNOSTAINING; Mild: The immunostaining extends along the meninges in the area with foreign material near a depression representing the start of a catheter track.

CD/PUT G-LT;
CATHETER TRACK (CT)
CT PIGMENTED MACROPHAGES; Multifocal; Moderate
CT GLIOSIS/ASTROCYTOSIS; Mild
CT FIBROSIS; Moderate
CT FOREIGN BODY REACTION; Multifocal; Minimal
CT MINERALIZED MATERIAL; Minimal
GDNF IMMUNOSTAINING; Mild

Pathology - Individual Animal Data (Concise Edition)
15-RS-288 - 40-Week Toxicity Stud of Recombinant-Methionyl Human Glial Cell Line-Derived Neurotrophic Factor (r-metHuGDNF) via Intermittent Bilateral Intraputamenal Convection-Enhanced Delivery in Rhesus Monkeys with a 12-Week Recovery Period

---

Animal Ref.: V002611 Group: 2 Sex: Male (continued)

Histo Pathology Observations:

CD/PUT G-RT;
- CATHETER TRACK (CT): A portion of the catheter track appears to have been pulled to the surface during catheter removal/necropsy. This displacement is an artefact.
- CT FIBROSIS; Moderate
- CT FOREIGN BODY REACTION; Multifocal; Mild
- CT GLIOSIS/ASTROCYTOSIS; Mild
- CT PIGMENTED MACROPHAGES; Multifocal; Mild
- CT MINERALIZED MATERIAL; Mild
- GDNF IMMUNOSTAINING; Mild

CD/PUT H-LT;
- INFILTRATION; Meninges; Mononuclear cell; Focal; Minimal
- CATHETER TRACK (CT)
- CT FIBROSIS; Moderate: There is mineralization of some of the collagen.
- CT GLIOSIS/ASTROCYTOSIS; Moderate
- CT INFILTRATION, MONONUCLEAR CELL; Multifocal; Minimal
- CT PIGMENTED MACROPHAGES; Multifocal; Moderate
- CT VACUOLATED MACROPHAGES; Multifocal; Mild
- CT FOREIGN BODY REACTION; Multifocal; Minimal
- CT VACUOLATION, WHITE MATTER; Multifocal; Minimal
- CT MINERALIZED MATERIAL; Minimal
- GDNF IMMUNOSTAINING; Moderate

CD/PUT H-RT;
- CATHETER TRACK (CT)
- CT FIBROSIS; Mild
- CT GLIOSIS/ASTROCYTOSIS; Mild
- CT PIGMENTED MACROPHAGES; Multifocal; Mild
- CT VACUOLATED MACROPHAGES; Multifocal; Minimal
- CT INFILTRATION, EOSINOPHIL; Multifocal; Minimal
- GDNF IMMUNOSTAINING; Moderate

CD/PUT/AC I-LT;
- CATHETER TRACK (CT)
- CT FIBROSIS; Mild

Pathology - Individual Animal Data (Concise Edition)
15-RS-288 - 40-Week Toxicity Stud of Recombinant-Methionyl Human Glial Cell Line-Derived Neurotrophic Factor (r-metHuGDNF) via Intermittent Bilateral Intraputamenal Convection-Enhanced Delivery in Rhesus Monkeys with a 12-Week Recovery Period

---

Animal Ref.: V002611 Group: 2 Sex: Male (continued)

Histo Pathology Observations:

CD/PUT/AC I-LT (continued);
- CT PERIVASCULAR CUFFS; Mixed; Focal; Minimal
- CT PIGMENTED MACROPHAGES; Multifocal; Mild
- CT FOREIGN BODY REACTION; Multifocal; Minimal
- CT VACUOLATION, WHITE MATTER; Focal; Minimal
- CT INFILTRATION, MONONUCLEAR CELL; Multifocal; Minimal
- CT INFILTRATION, EOSINOPHIL; Multifocal; Minimal
- CT GLIOSIS/ASTROCYTOSIS; Mild
- CT VACUOLATED MACROPHAGES; Multifocal; Minimal
- GDNF IMMUNOSTAINING; Moderate

CD/PUT/GP J-LT;
- GDNF IMMUNOSTAINING; Mild: There is focal staining although a catheter track is not visible.

CD/PUT/GP J-RT;
- GDNF IMMUNOSTAINING; Mild: There is focal staining although a catheter track is not visible.

PUT/GP K-LT;
- FIBROSIS; Meninges; Focal; Mild

PUT/GP K-RT;
- LFB/PAS slide present.

THAL/SN M;
- LFB/PAS slide present.
- GDNF IMMUNOSTAINING; Minimal: There is focal staining in the SN, bilaterall- y, although a catheter track is not visible.

THAL/STN L;
- LFB/PAS slides (2) present.

The following tissues were within normal limits:

BRAIN D	CB/MO T-CB	CB/MO T-LT	CB/MO T-RT	CB R-CB	CB V-CB	PUT/GP K-RT
SN N	THAL/STN L	SPINAL CORD, CERVICAL		SPINAL CORD, THORACIC		SPINAL CORD, LUMBAR

Pathology - Individual Animal Data (Concise Edition)
15-RS-288 - 40-Week Toxicity Stud of Recombinant-Methionyl Human Glial Cell Line-Derived Neurotrophic Factor (r-metHuGDNF) via Intermittent Bilateral Intraputamenal Convection-Enhanced Delivery in Rhesus Monkeys with a 12-Week Recovery Period

---

Animal Ref.: V002611 Group: 2 Sex: Male (continued)

The following tissues were within normal limits: (continued)

DRG, CERVICAL DRG, THORACIC DRG, LUMBAR TRIGEMINAL GANGLIA

Pathology - Individual Animal Data (Concise Edition)
15-RS-288 - 40-Week Toxicity Stud of Recombinant-Methionyl Human Glial Cell Line-Derived Neurotrophic Factor (r-metHuGDNF) via Intermittent Bilateral Intraputamenal Convection-Enhanced Delivery in Rhesus Monkeys with a 12-Week Recovery Period

---

Animal Ref.: V002614 Group: 2 Sex: Male Species: Monkey Strain: Rhesus

Test Material: r-metHuGDNF Dose: MG 87.1 µg Route: See Protocol Study Type: Regulated Repeat Dose
Date of Death : 07/17/2015 Study Day No. (Week): 367 (53) Mode of Death: Recovery Sacrifice
Date of Necropsy: 07/17/2015 ** NECROPSY COMPLETE **

** EXAMINATION COMPLETE **

Histo Pathology Observations:

BRAIN D;
INFILTRATION; Meninges; Mononuclear cell; Multifocal; Minimal

CB R-CB;
There is a fixation or histologic artefact at the tip of the folia in one focus.

CD/PUT G-LT;
CATHETER TRACK (CT)
CT FIBROSIS; Mild
CT FOREIGN BODY REACTION; Focal; Minimal
CT MINERALIZED MATERIAL; Minimal
GDNF IMMUNOSTAINING; Minimal: The GDNF immunostaining is in the catheter track and fibrous capsule.

CD/PUT H-LT;
FIBROSIS; Meninges; Focal; Minimal
CATHETER TRACK (CT)
CT FIBROSIS; Minimal
CT PIGMENTED MACROPHAGES; Focal; Minimal
CT FOREIGN BODY REACTION; Focal; Minimal
CT MINERALIZED MATERIAL; Minimal
GDNF IMMUNOSTAINING; Minimal

CD/PUT H-RT;
CATHETER TRACK (CT)
CT PIGMENTED MACROPHAGES; Multifocal; Minimal
CT VACUOLATION, WHITE MATTER; Focal; Minimal
CT FOREIGN BODY REACTION; Focal; Minimal
CT AXON SPHEROIDS; Focal; Minimal
CT MINERALIZED MATERIAL; Minimal

Animal Ref.: V002614 Group: 2 Sex: Male (continued)

Histo Pathology Observations:

CD/PUT H-RT (continued);
GDNF IMMUNOSTAINING; Mild

CD/PUT I-RT;
CATHETER TRACK (CT)
CT FOREIGN BODY REACTION; Focal; Minimal
CT PIGMENTED MACROPHAGES; Focal; Minimal
CT VACUOLATION, WHITE MATTER; Focal; Minimal
CT GLIOSIS/ASTROCYTOSIS; Focal; Minimal
CT MINERALIZED MATERIAL; Minimal
GDNF IMMUNOSTAINING; Minimal

CD/PUT/AC I-LT;
CATHETER TRACK (CT)
CT FIBROSIS; Mild
CT PIGMENTED MACROPHAGES; Multifocal; Minimal
CT FOREIGN BODY REACTION; Multifocal; Minimal
CT VACUOLATION, WHITE MATTER; Multifocal; Minimal
CT GLIOSIS/ASTROCYTOSIS; Minimal
GDNF IMMUNOSTAINING; Minimal

CD/PUT/GP J-RT;
CATHETER TRACK (CT)
CT FIBROSIS; Mild
CT PIGMENTED MACROPHAGES; Multifocal; Minimal
CT MINERALIZED MATERIAL; Minimal
GDNF IMMUNOSTAINING; Mild

PUT/GP K-RT;
LFB/PAS slide present.

THAL/SN M;
LFB/PAS slide present.

THAL/STN L;
LFB/PAS slides (2) present.

Pathology - Individual Animal Data (Concise Edition)
15-RS-288 - 40-Week Toxicity Stud of Recombinant-Methionyl Human Glial Cell Line-Derived Neurotrophic Factor (r-metHuGDNF) via Intermittent Bilateral Intraputamenal Convection-Enhanced Delivery in Rhesus Monkeys with a 12-Week Recovery Period

---

Animal Ref.: V002614 Group: 2 Sex: Male (continued)

Histo Pathology Observations:

THAL/STN L1-LT;
INFILTRATION; Meninges; Mononuclear cell; Focal; Minimal

DRG, LUMBAR;
VACUOLATION; Ganglion cell; Minimal

The following tissues were within normal limits:

BS/PONS O-LT	BS/PONS O-RT	CB/MO U-CB	CB/MO U-LT	CB/MO U-RT	CB R-CB	CB V-CB
CD/PUT G-RT	CD/PUT/GP J-LT	PUT/GP K-LT	PUT/GP K-RT	SN N	THAL/SN M	THAL/STN L
SPINAL CORD, CERVICAL		SPINAL CORD, THORACIC		SPINAL CORD, LUMBAR	DRG, CERVICAL	DRG, THORACIC

The following tissues have not been examined:

TRIGEMINAL GANGLIA; INSUFFICIENT TISSUE TO EVALUATE FOLLOWING RECUT OR REHARVEST

Pathology - Individual Animal Data (Concise Edition)
15-RS-288 - 40-Week Toxicity Stud of Recombinant-Methionyl Human Glial Cell Line-Derived Neurotrophic Factor (r-metHuGDNF) via Intermittent Bilateral Intraputamenal Convection-Enhanced Delivery in Rhesus Monkeys with a 12-Week Recovery Period

---

Animal Ref.: V002615 Group: 2 Sex: Male Species: Monkey Strain: Rhesus

Test Material: r-metHuGDNF Dose: MG 87.1 µg Route: See Protocol Study Type: Regulated Repeat Dose
Date of Death : 04/27/2015 Study Day No. (Week): 285 (41) Mode of Death: Terminal Sacrifice
Date of Necropsy: 04/27/2015 ** NECROPSY COMPLETE **

** EXAMINATION COMPLETE **

Histo Pathology Observations:

CB/MO T-CB;
LFB slide present.

CB R-CB;
LFB slide present.

CD F-LT;
GDNF IMMUNOSTAINING; Minimal: The immunostaining is located in the catheter track at the surface of the brain. This track is visible in the GDNF-stained slide.

CD F-RT;
FIBROSIS; Meninges; Focal; Minimal
CATHETER TRACK (CT)
CT FOREIGN BODY REACTION; Multifocal; Minimal
CT FIBROSIS; Minimal
CT GLIOSIS/ASTROCYTOSIS; Minimal
CT VACUOLATED MACROPHAGES; Focal; Minimal
CT PIGMENTED MACROPHAGES; Multifocal; Mild
CT MINERALIZED MATERIAL; Mild
GDNF IMMUNOSTAINING; Moderate: In addition to the immunostaining in and around the catheter track, there is immunostaining along the surface of the meninges, suggesting some leakage of GDNF onto the surface.

CD/PUT G-LT;
CATHETER TRACK (CT): The catheter track is evident in the GDNF immunostained slide, so the entry of catheter track present is made based on that slide.
GDNF IMMUNOSTAINING; Minimal: There is GDNF immunostaining in the catheter track, which is visible in the GDNF-stained slide. The catheter track was not present in the H&E slide.

Pathology - Individual Animal Data (Concise Edition)
15-RS-288 - 40-Week Toxicity Stud of Recombinant-Methionyl Human Glial Cell Line-Derived Neurotrophic Factor (r-metHuGDNF) via Intermittent Bilateral Intraputamenal Convection-Enhanced Delivery in Rhesus Monkeys with a 12-Week Recovery Period

---

Animal Ref.: V002615 Group: 2 Sex: Male (continued)

Histo Pathology Observations:

CD/PUT G-RT;
CATHETER TRACK (CT)
CT FIBROSIS; Minimal
CT FOREIGN BODY REACTION; Multifocal; Minimal
CT GLIOSIS/ASTROCYTOSIS; Minimal
CT INFILTRATION, MONONUCLEAR CELL; Focal; Minimal
CT PIGMENTED MACROPHAGES; Multifocal; Minimal
GDNF IMMUNOSTAINING; Moderate

CD/PUT H-LT;
INFILTRATION; Meninges; Mononuclear cell; Focal; Minimal
CATHETER TRACK (CT)
CT FIBROSIS; Minimal
CT GLIOSIS/ASTROCYTOSIS; Minimal
CT FOREIGN BODY REACTION; Multifocal; Minimal
GDNF IMMUNOSTAINING; Moderate

CD/PUT H-RT;
CATHETER TRACK (CT)
CT FIBROSIS; Moderate
CT GLIOSIS/ASTROCYTOSIS; Minimal
CT INFILTRATION, MONONUCLEAR CELL; Focal; Minimal
CT PIGMENTED MACROPHAGES; Multifocal; Minimal
CT FOREIGN BODY REACTION; Multifocal; Minimal
CT INFILTRATION, EOSINOPHIL; Multifocal; Minimal
GDNF IMMUNOSTAINING; Moderate

CD/PUT/AC I-LT;
CATHETER TRACK (CT)
CT PIGMENTED MACROPHAGES; Focal; Minimal
CT INFILTRATION, MONONUCLEAR CELL; Focal; Minimal
CT INFILTRATION, EOSINOPHIL; Focal; Minimal
CT GLIOSIS/ASTROCYTOSIS; Minimal
GDNF IMMUNOSTAINING; Moderate

Pathology - Individual Animal Data (Concise Edition)
15-RS-288 - 40-Week Toxicity Stud of Recombinant-Methionyl Human Glial Cell Line-Derived Neurotrophic Factor (r-metHuGDNF) via Intermittent Bilateral Intraputamenal Convection-Enhanced Delivery in Rhesus Monkeys with a 12-Week Recovery Period

---

Animal Ref.: V002615 Group: 2 Sex: Male (continued)

Histo Pathology Observations:

PUT/GP K-LT;
LFB/PAS slide present.

PUT/GP K-RT;
INFILTRATION; Choroid plexus; Mononuclear cell; Focal; Minimal

THAL/SN M;
LFB/PAS slide present.

THAL/STN L;
LFB/PAS slides (2) present.

DRG, CERVICAL;
VACUOLATION; Ganglion cell; Minimal

The following tissues were within normal limits:

BRAIN D	BS/PONS O-LT	BS/PONS O-RT	CB/MO T-CB	CB/MO T-LT	CB/MO T-RT	CB R-CB
CB V-CB	CD/PUT/GP J-LT	CD/PUT/GP J-RT	PUT/GP K-LT	SN N	THAL/SN M	THAL/STN L
SPINAL CORD, CERVICAL		SPINAL CORD, THORACIC		SPINAL CORD, LUMBAR	DRG, THORACIC	DRG, LUMBAR
TRIGEMINAL GANGLIA						

Pathology - Individual Animal Data (Concise Edition)
15-RS-288 - 40-Week Toxicity Stud of Recombinant-Methionyl Human Glial Cell Line-Derived Neurotrophic Factor (r-metHuGDNF) via Intermittent Bilateral Intraputamenal Convection-Enhanced Delivery in Rhesus Monkeys with a 12-Week Recovery Period

---

Animal Ref.: V001633 Group: 3 Sex: Male Species: Monkey Strain: Rhesus

Test Material: r-metHuGDNF Dose: SAT 87.1 µg Route: See Protocol Study Type: Regulated Repeat Dose
Date of Death : 06/05/2015 Study Day No. (Week): 292 (42) Mode of Death: Terminal Satellite Sacrifice
Date of Necropsy: 06/05/2015 ** NECROPSY COMPLETE **

** EXAMINATION COMPLETE **

Histo Pathology Observations:

CB/MO T-CB;
LFB slide present.

CB/MO T-LT;
LFB slide present.

CB R-CB;
LFB slide present.

CB V-CB;
The slide is cerebellum and it is labeled V-CB.

CD F-LT;
CATHETER TRACK (CT)
CT PIGMENTED MACROPHAGES; Focal; Minimal
CT FIBROSIS; Focal; Minimal
CT FOREIGN BODY REACTION; Focal; Minimal
GDNF IMMUNOSTAINING; Moderate

CD F-RT;
CATHETER TRACK (CT)
CT FOREIGN BODY REACTION; Multifocal; Minimal
CT FIBROSIS; Minimal
GDNF IMMUNOSTAINING; Moderate

CD/PUT G-LT;
CATHETER TRACK (CT): The track is in two segments.
CT PIGMENTED MACROPHAGES; Multifocal; Minimal
CT INFILTRATION, MONONUCLEAR CELL; Multifocal; Minimal
CT FIBROSIS; Minimal
CT FOREIGN BODY REACTION; Multifocal; Minimal

Animal Ref.: V001633 Group: 3 Sex: Male (continued)

Histo Pathology Observations:

CD/PUT G-LT (continued);
CT HEMORRHAGE; Multifocal; Minimal
GDNF IMMUNOSTAINING; Moderate

CD/PUT G-RT;
CATHETER TRACK (CT): The catheter track is in two segments.
CT FOREIGN BODY REACTION; Multifocal; Minimal
CT INFILTRATION, MONONUCLEAR CELL; Focal; Minimal
CT INFILTRATION, EOSINOPHIL; Focal; Minimal
CT PERIVASCULAR CUFFS; Mixed; Focal; Minimal
CT MINERALIZED MATERIAL; Minimal
GDNF IMMUNOSTAINING; Moderate

CD/PUT H-LT;
Slide is labeled CD/PUT/AC H-LT
CATHETER TRACK (CT): The catheter track is in two segments.
CT FIBROSIS; Mild
CT INFILTRATION, MONONUCLEAR CELL; Multifocal; Minimal
CT INFILTRATION, EOSINOPHIL; Multifocal; Minimal
CT MINERALIZED MATERIAL; Minimal
GDNF IMMUNOSTAINING; Moderate

CD/PUT H-RT;
Slide is labeled CD/PUT/AC H-RT.
CATHETER TRACK (CT)
CT FIBROSIS; Minimal
CT INFILTRATION, MONONUCLEAR CELL; Focal; Minimal
CT PIGMENTED MACROPHAGES; Multifocal; Minimal
CT FOREIGN BODY REACTION; Multifocal; Minimal
CT MINERALIZED MATERIAL; Minimal
GDNF IMMUNOSTAINING; Moderate

CD/PUT I-RT;
The slide is labeled CD/Put/GP I-rt.
CATHETER TRACK (CT)
CT FOREIGN BODY REACTION; Multifocal; Minimal

Animal Ref.: V001633 Group: 3 Sex: Male (continued)

Histo Pathology Observations:

CD/PUT I-RT (continued);
CT PIGMENTED MACROPHAGES; Multifocal; Minimal
CT INFILTRATION, MONONUCLEAR CELL; Focal; Minimal
CT FIBROSIS; Focal; Minimal

CD/PUT/AC I-LT;
Slide is labeled CD/PUT/GP I-LT.
CATHETER TRACK (CT)
CT FIBROSIS; Minimal
CT PIGMENTED MACROPHAGES; Focal; Minimal
CT FOREIGN BODY REACTION; Focal; Minimal
CT INFILTRATION, MONONUCLEAR CELL; Focal; Minimal
GDNF IMMUNOSTAINING; Moderate

CD/PUT/GP J-LT;
LFB/PAS slide present. The slides are labeled Put/GP J-lt.
AXON SPHEROIDS; Focal; Mild: Axon spheroids observed in H&E stained slide and they fluoresce in FJC stained slide.

CD/PUT/GP J-RT;
The slide is labeled Put/GP J-rt.

SN M;
LFB/PAS slide present, but it is labeled SN N. The GDNF slide is also labeled SN N.

THAL/SN L;
LFB/PAS slide present, but it is labeled Thal/STN L.
INFILTRATION; Perivascular; Mononuclear cell; Focal; Minimal
AXON SPHEROIDS; Focal; Minimal: Axon spheroids observed in H&E stained slide and they fluoresce in FJC stained slide.

THAL/STN K-LT;
AXON SPHEROIDS; Focal; Minimal

Animal Ref.: V001633 Group: 3 Sex: Male (continued)

Histo Pathology Observations:

THAL/STN K-RT;
LFB/PAS slide present.

SPINAL CORD, CERVICAL;
There are bone fragments and cells in the meninges that appear to be necropsy (post-mortem) artefact.

The following tissues were within normal limits:

BRAIN D	BS/PONS O-LT	BS/PONS O-RT	CB/MO T-CB	CB/MO T-LT	CB/MO T-RT	CB R-CB
CB V-CB	CD/PUT/GP J-RT	SN M	THAL/STN K-RT	SPINAL CORD, CERVICAL		SPINAL CORD, THORACIC
SPINAL CORD, LUMBAR	DRG, CERVICAL	DRG, THORACIC	DRG, LUMBAR	TRIGEMINAL GANGLIA		

Pathology - Individual Animal Data (Concise Edition)
15-RS-288 - 40-Week Toxicity Stud of Recombinant-Methionyl Human Glial Cell Line-Derived Neurotrophic Factor (r-metHuGDNF) via Intermittent Bilateral Intraputamenal Convection-Enhanced Delivery in Rhesus Monkeys with a 12-Week Recovery Period

---

Animal Ref.: V001963 Group: 3 Sex: Male Species: Monkey Strain: Rhesus

Test Material: r-metHuGDNF Dose: SAT 87.1 µg Route: See Protocol Study Type: Regulated Repeat Dose
Date of Death : 06/04/2015 Study Day No. (Week): 291 (42) Mode of Death: Terminal Satellite Sacrifice
Date of Necropsy: 06/04/2015 ** NECROPSY COMPLETE **

** EXAMINATION COMPLETE **

Histo Pathology Observations:

BRAIN D;
FIBROSIS; Meninges; Focal; Minimal

CB/MO U-CB;
LFB slide present.

CB S-CB;
LFB slide present.

CD G-LT;
CATHETER TRACK (CT)
CT FOREIGN BODY REACTION; Focal; Minimal
CT INFILTRATION, MONONUCLEAR CELL; Focal; Minimal
CT VACUOLATION, WHITE MATTER; Multifocal; Minimal
GDNF IMMUNOSTAINING; Moderate

CD G-RT;
CATHETER TRACK (CT)
CT PIGMENTED MACROPHAGES; Focal; Minimal
CT GLIOSIS/ASTROCYTOSIS; Minimal
CT VACUOLATED MACROPHAGES; Multifocal; Minimal
CT VACUOLATION, WHITE MATTER; Focal; Mild
GDNF IMMUNOSTAINING; Moderate

CD/PUT H-LT;
CATHETER TRACK (CT)
CT GLIOSIS/ASTROCYTOSIS; Mild
CT PIGMENTED MACROPHAGES; Multifocal; Mild
CT FOREIGN BODY REACTION; Focal; Minimal
CT VACUOLATION, GRAY MATTER; Focal; Minimal
CT MINERALIZED MATERIAL; Minimal

Animal Ref.: V001963 Group: 3 Sex: Male (continued)

Histo Pathology Observations:

CD/PUT H-LT (continued);
GDNF IMMUNOSTAINING; Moderate

CD/PUT H-RT;
CATHETER TRACK (CT): Catheter track is in 4 parts.
CT FIBROSIS; Minimal
CT GLIOSIS/ASTROCYTOSIS; Minimal
CT INFILTRATION, MONONUCLEAR CELL; Focal; Minimal
CT PIGMENTED MACROPHAGES; Multifocal; Minimal
CT VACUOLATED MACROPHAGES; Multifocal; Mild
CT VACUOLATION, WHITE MATTER; Multifocal; Minimal
CT FOREIGN BODY REACTION; Focal; Minimal
CT VACUOLATION, GRAY MATTER; Multifocal; Minimal
CT MINERALIZED MATERIAL; Minimal
GDNF IMMUNOSTAINING; Moderate

CD/PUT I-RT;
CATHETER TRACK (CT)
CT FOREIGN BODY REACTION; Multifocal; Minimal
CT PIGMENTED MACROPHAGES; Multifocal; Minimal
CT FIBROSIS; Multifocal; Minimal
CT GLIOSIS/ASTROCYTOSIS; Multifocal; Minimal
CT VACUOLATION, GRAY MATTER; Multifocal; Minimal
CT MINERALIZED MATERIAL; Minimal
GDNF IMMUNOSTAINING; Moderate

CD/PUT/AC I-LT;
CATHETER TRACK (CT)
CT PIGMENTED MACROPHAGES; Multifocal; Minimal
CT FOREIGN BODY REACTION; Multifocal; Minimal
CT VACUOLATION, WHITE MATTER; Focal; Minimal
CT INFILTRATION, MONONUCLEAR CELL; Multifocal; Minimal
CT GLIOSIS/ASTROCYTOSIS; Minimal
CT MINERALIZED MATERIAL; Minimal
GDNF IMMUNOSTAINING; Moderate

Animal Ref.: V001963 Group: 3 Sex: Male (continued)

Histo Pathology Observations:

PUT/GP K-LT;
LFB/PAS slide present.

SN N;
INFILTRATION; Meninges; Mononuclear cell; Focal; Minimal

THAL/SN M-LT;
LFB/PAS slide present.

THAL/STN L-LT;
LFB/PAS slide present.

THAL/STN L-RT;
LFB/PAS slide present.

The following tissues were within normal limits:

BS/PONS O-LT	BS/PONS O-RT	CB/MO U-CB	CB/MO U-LT	CB/MO U-RT	CB S-CB	CB V-CB
CD/PUT/GP J-LT	CD/PUT/GP J-RT	PUT/GP K-LT	PUT/GP K-RT	THAL/SN M-LT	THAL/SN M-RT	THAL/STN L-LT
THAL/STN L-RT	SPINAL CORD, CERVICAL		SPINAL CORD, THORACIC		SPINAL CORD, LUMBAR	DRG, CERVICAL
DRG, THORACIC	DRG, LUMBAR	TRIGEMINAL GANGLIA				

Pathology - Individual Animal Data (Concise Edition)
15-RS-288 - 40-Week Toxicity Stud of Recombinant-Methionyl Human Glial Cell Line-Derived Neurotrophic Factor (r-metHuGDNF) via Intermittent Bilateral Intraputamenal Convection-Enhanced Delivery in Rhesus Monkeys with a 12-Week Recovery Period

---

Animal Ref.: V002043   Group: 3   Sex: Male   Species: Monkey   Strain: Rhesus

Test Material: r-metHuGDNF   Dose: SAT 87.1 µg   Route: See Protocol   Study Type: Regulated Repeat Dose
Date of Death : 06/04/2015   Study Day No. (Week): 291 (42)   Mode of Death: Terminal Satellite Sacrifice
Date of Necropsy: 06/04/2015   ** NECROPSY COMPLETE **

** EXAMINATION COMPLETE **

Histo Pathology Observations:

BRAIN D;
FIBROSIS; Meninges; Focal; Minimal

CB/MO T-CB;
LFB slide present.

CB R-CB;
LFB slide present.

CD G-LT;
CATHETER TRACK (CT)
CT FOREIGN BODY REACTION; Multifocal; Minimal
CT INFILTRATION, MONONUCLEAR CELL; Multifocal; Minimal

CD G-RT;
CATHETER TRACK (CT)
CT FOREIGN BODY REACTION; Multifocal; Minimal
CT GLIOSIS/ASTROCYTOSIS; Minimal
CT VACUOLATION, WHITE MATTER; Multifocal; Minimal
GDNF IMMUNOSTAINING; Moderate

CD/PUT H-LT;
CATHETER TRACK (CT)
CT GLIOSIS/ASTROCYTOSIS; Minimal
CT INFILTRATION, MONONUCLEAR CELL; Focal; Minimal
CT FOREIGN BODY REACTION; Multifocal; Mild
GDNF IMMUNOSTAINING; Moderate

CD/PUT H-RT;
CATHETER TRACK (CT)
CT FIBROSIS; Mild

Animal Ref.: V002043 Group: 3 Sex: Male (continued)

Histo Pathology Observations:

CD/PUT H-RT (continued);
CT INFILTRATION, MONONUCLEAR CELL; Focal; Minimal
CT VACUOLATION, WHITE MATTER; Focal; Minimal
CT FOREIGN BODY REACTION; Multifocal; Minimal
CT MINERALIZED MATERIAL; Minimal
GDNF IMMUNOSTAINING; Moderate

CD/PUT I-RT;
CATHETER TRACK (CT)
CT FOREIGN BODY REACTION; Multifocal; Minimal
CT PIGMENTED MACROPHAGES; Focal; Minimal
CT FIBROSIS; Focal; Minimal
GDNF IMMUNOSTAINING; Marked

CD/PUT/AC I-LT;
CATHETER TRACK (CT)
CT FIBROSIS; Minimal
CT PIGMENTED MACROPHAGES; Multifocal; Minimal
CT VACUOLATION, WHITE MATTER; Focal; Minimal
CT INFILTRATION, MONONUCLEAR CELL; Multifocal; Minimal
GDNF IMMUNOSTAINING; Moderate

CD/PUT/GP J-LT;
CATHETER TRACK (CT)
CT FOREIGN BODY REACTION; Multifocal; Minimal
CT GLIOSIS/ASTROCYTOSIS; Focal; Minimal
CT MINERALIZED MATERIAL; Minimal
GDNF IMMUNOSTAINING; Moderate

CD/PUT/GP J-RT;
CATHETER TRACK (CT)
CT GLIOSIS/ASTROCYTOSIS; Minimal
CT FOREIGN BODY REACTION; Multifocal; Mild
CT MINERALIZED MATERIAL; Minimal

Pathology - Individual Animal Data (Concise Edition)
15-RS-288 - 40-Week Toxicity Stud of Recombinant-Methionyl Human Glial Cell Line-Derived Neurotrophic Factor (r-metHuGDNF) via Intermittent Bilateral Intraputamenal Convection-Enhanced Delivery in Rhesus Monkeys with a 12-Week Recovery Period

Animal Ref.: V002043 Group: 3 Sex: Male (continued)

Histo Pathology Observations:

PUT/GP K-RT;
LFB/PAS slide present.

THAL/SN M;
LFB/PAS slide present.

THAL/STN L-LT;
LFB/PAS slide present.

THAL/STN L-RT;
LFB/PAS slide present.

The following tissues were within normal limits:

BS/PONS O-LT	BS/PONS O-RT	CB/MO T-CB	CB/MO T-LT	CB/MO T-RT	CB R-CB	CB V-CB
PUT/GP K-LT	PUT/GP K-RT	SN N	THAL/SN M	THAL/STN L-LT	THAL/STN L-RT	SPINAL CORD, CERVICAL
SPINAL CORD, THORACIC		SPINAL CORD, LUMBAR	DRG, CERVICAL	DRG, THORACIC	DRG, LUMBAR	TRIGEMINAL GANGLIA

Pathology - Individual Animal Data (Concise Edition)
15-RS-288 - 40-Week Toxicity Stud of Recombinant-Methionyl Human Glial Cell Line-Derived Neurotrophic Factor (r-metHuGDNF) via Intermittent Bilateral Intraputamenal Convection-Enhanced Delivery in Rhesus Monkeys with a 12-Week Recovery Period

---

Animal Ref.: V002603 Group: 3 Sex: Male Species: Monkey Strain: Rhesus

Test Material: r-metHuGDNF Dose: SAT 87.1 µg Route: See Protocol Study Type: Regulated Repeat Dose
Date of Death : 06/05/2015 Study Day No. (Week): 292 (42) Mode of Death: Terminal Satellite Sacrifice
Date of Necropsy: 06/05/2015 ** NECROPSY COMPLETE **

** EXAMINATION COMPLETE **

Histo Pathology Observations:

BRAIN D;
CATHETER TRACK (CT)
CT MINERALIZED MATERIAL; Focal; Minimal

CB/MO T-CB;
LFB slide present.

CB R-CB;
LFB slide present.

CD/PUT G-LT;
CATHETER TRACK (CT)
CT PIGMENTED MACROPHAGES; Focal; Minimal
CT FOREIGN BODY REACTION; Focal; Minimal
CT VACUOLATION, WHITE MATTER; Focal; Minimal
CT MINERALIZED MATERIAL; Minimal
GDNF IMMUNOSTAINING; Moderate: The immunostaining involves the surface and a sulcus and appears to be at the start of a catheter site at the surface. The deeper catheter track does not show immunostaining.

CD/PUT G-RT;
CATHETER TRACK (CT)
CT FOREIGN BODY REACTION; Focal; Minimal
CT PIGMENTED MACROPHAGES; Focal; Minimal
CT VACUOLATION, WHITE MATTER; Focal; Minimal
CT MINERALIZED MATERIAL; Minimal

CD/PUT H-LT;
FIBROSIS; Meninges; Focal; Minimal
CATHETER TRACK (CT)
CT PIGMENTED MACROPHAGES; Focal; Minimal

Pathology - Individual Animal Data (Concise Edition)
15-RS-288 - 40-Week Toxicity Stud of Recombinant-Methionyl Human Glial Cell Line-Derived Neurotrophic Factor (r-metHuGDNF) via Intermittent Bilateral Intraputamenal Convection-Enhanced Delivery in Rhesus Monkeys with a 12-Week Recovery Period

Animal Ref.: V002603 Group: 3 Sex: Male (continued)

Histo Pathology Observations:

CD/PUT H-LT (continued);
CT FOREIGN BODY REACTION; Focal; Minimal
CT MINERALIZED MATERIAL; Minimal
GDNF IMMUNOSTAINING; Moderate: The immunostaining involves the surface and the neuropil below the surface whereas the deeper catheter track does not show immunostaining.

CD/PUT H-RT;
CATHETER TRACK (CT)
CT GLIOSIS/ASTROCYTOSIS; Minimal
CT PIGMENTED MACROPHAGES; Multifocal; Minimal
CT VACUOLATION, WHITE MATTER; Focal; Minimal
CT FOREIGN BODY REACTION; Focal; Minimal

CD/PUT I-RT;
GDNF IMMUNOSTAINING; Mild: A catheter track is not visible, but there is immunostaining in the neuropil that is considered specific for GDNF.

CD/PUT/AC I-LT;
CATHETER TRACK (CT)
GDNF IMMUNOSTAINING; Moderate

CD/PUT/GP J-LT;
CATHETER TRACK (CT)
CT FOREIGN BODY REACTION; Focal; Minimal
CT PIGMENTED MACROPHAGES; Multifocal; Minimal
CT FIBROSIS; Focal; Minimal
CT MINERALIZED MATERIAL; Minimal
GDNF IMMUNOSTAINING; Moderate

CD/PUT/GP J-RT;
CATHETER TRACK (CT)
CT FIBROSIS; Minimal
CT FOREIGN BODY REACTION; Focal; Minimal
CT PIGMENTED MACROPHAGES; Focal; Minimal
CT MINERALIZED MATERIAL; Minimal

Pathology - Individual Animal Data (Concise Edition)
15-RS-288 - 40-Week Toxicity Stud of Recombinant-Methionyl Human Glial Cell Line-Derived Neurotrophic Factor (r-metHuGDNF) via Intermittent Bilateral Intraputamenal Convection-Enhanced Delivery in Rhesus Monkeys with a 12-Week Recovery Period

Animal Ref.: V002603 Group: 3 Sex: Male (continued)

Histo Pathology Observations:

CD/PUT/GP J-RT (continued);
GDNF IMMUNOSTAINING; Moderate

PUT/GP K-LT;
LFB/PAS slide present.
GDNF IMMUNOSTAINING; Mild: A catheter track is not visible, but there is immunostaining in the neuropil that is considered specific for GDNF.

PUT/GP K-RT;
CATHETER TRACK (CT)
CT FIBROSIS; Focal; Minimal
CT PIGMENTED MACROPHAGES; Focal; Minimal
GDNF IMMUNOSTAINING; Moderate

THAL/SN M;
LFB/PAS slide present.

THAL/STN L;
LFB/PAS slides (2) present.

The following tissues were within normal limits:

BS/PONS O-LT	BS/PONS O-RT	CB/MO T-CB	CB/MO T-LT	CB/MO T-RT	CB R-CB	CB V-CB
SN N	THAL/SN M	THAL/STN L	SPINAL CORD, CERVICAL		SPINAL CORD, THORACIC	
SPINAL CORD, LUMBAR	DRG, CERVICAL	DRG, THORACIC	DRG, LUMBAR	TRIGEMINAL GANGLIA		

End Of Print

Table 1-3A. Histopathology Cross Reference Table—Terminal

Pathology - Histo Pathology Observations - Animal Cross Reference
15-RS-288 - 40-Week Toxicity Stud of Recombinant-Methionyl Human Glial Cell Line-Derived Neurotrophic Factor (r-metHuGDNF) via Intermittent Bilateral Intraputamenal Convection-Enhanced Delivery in Rhesus Monkeys with a 12-Week Recovery Period

Removal Reason: Terminal Sacrifice

	---- MALES -----	
	MC 0 µg	MG 87.1 µg
BRAIN D;		
Examined	(3)	(5)
Within Normal Limits	V000909<br>V002604<br>V002606	V001946<br>V002593<br>V002611<br>V002615
CT MINERALIZED MATERIAL; Focal; Minimal		V001954
BS/PONS O-LT;		
Examined	(0)	(3)
Within Normal Limits		V001954<br>V002593<br>V002615
BS/PONS O-RT;		
Examined	(0)	(3)
Within Normal Limits		V001954<br>V002593<br>V002615
BS/PONS P-LT;		
Examined	(3)	(2)
Within Normal Limits	V002606	V001946
Meninges; FIBROSIS; Focal; Minimal	V000909<br>V002604	V002611
BS/PONS P-RT;		
Examined	(3)	(2)
Within Normal Limits	V002604<br>V002606	V001946
Meninges; FIBROSIS; Focal; Minimal		V002611

Pathology - Histo Pathology Observations - Animal Cross Reference
15-RS-288 - 40-Week Toxicity Stud of Recombinant-Methionyl Human Glial Cell Line-Derived Neurotrophic Factor (r-metHuGDNF) via Intermittent Bilateral Intraputamenal Convection-Enhanced Delivery in Rhesus Monkeys with a 12-Week Recovery Period

Removal Reason: Terminal Sacrifice

	---- MALES -----	
	MC 0 µg	MG 87.1 µg
**BS/PONS P-RT; (continued)**		
Meninges; FIBROSIS; Multifocal; Mild	V000909	
**CB/MO T-CB;**		
Examined	(3)	(5)
Within Normal Limits	V000909<br>V002604<br>V002606	V001946<br>V001954<br>V002593<br>V002611<br>V002615
**CB/MO T-LT;**		
Examined	(3)	(5)
Within Normal Limits	V002604<br>V002606	V001946<br>V001954<br>V002593<br>V002611<br>V002615
Meninges; FIBROSIS; Focal; Mild	V000909	
**CB/MO T-RT;**		
Examined	(3)	(5)
Within Normal Limits	V000909<br>V002604<br>V002606	V001946<br>V001954<br>V002593<br>V002611<br>V002615
**CB R-CB;**		
Examined	(3)	(5)
Within Normal Limits	V000909<br>V002604<br>V002606	V001946<br>V001954<br>V002593<br>V002611<br>V002615

Pathology - Histo Pathology Observations - Animal Cross Reference
15-RS-288 - 40-Week Toxicity Stud of Recombinant-Methionyl Human Glial Cell Line-Derived Neurotrophic Factor (r-metHuGDNF) via Intermittent Bilateral Intraputamenal Convection-Enhanced Delivery in Rhesus Monkeys with a 12-Week Recovery Period

Removal Reason: Terminal Sacrifice

	---- MALES -----	
	MC 0 µg	MG 87.1 µg
CB U-CB;		
Examined	(0)	(1)
Within Normal Limits		V001946
CB V-CB;		
Examined	(3)	(4)
Within Normal Limits	V000909 V002604 V002606	V001954 V002593 V002611 V002615
CD F-LT;		
Examined	(3)	(5)
Within Normal Limits	V002604 V002606	V001946 V002593
CATHETER TRACK (CT)	V000909	V001954 V002611
CT VACUOLATED MACROPHAGES; Multifocal;		
Mild	V000909	
CT PIGMENTED MACROPHAGES; Multifocal;		
Minimal	V000909	V001954
Moderate		V002611
CT INFILTRATION, MONONUCLEAR CELL; Multifocal;		
Minimal	V000909	
CT GLIOSIS/ASTROCYTOSIS;		
Minimal		V001954
Mild	V000909	V002611
CT HEMORRHAGE; Focal;		
Minimal	V000909	

Pathology - Histo Pathology Observations - Animal Cross Reference
15-RS-288 - 40-Week Toxicity Stud of Recombinant-Methionyl Human Glial Cell Line-Derived Neurotrophic Factor (r-metHuGDNF) via Intermittent Bilateral Intraputamenal Convection-Enhanced Delivery in Rhesus Monkeys with a 12-Week Recovery Period

Removal Reason: Terminal Sacrifice

	MALES	
	MC 0 µg	MG 87.1 µg
CD F-LT; (continued)		
CT FIBROSIS; Focal;		
Minimal		V001954
CT INFILTRATION, EOSINOPHIL; Multifocal;		
Minimal		V001954
CT AXON SPHEROIDS;		
Minimal	V000909	
CT MINERALIZED MATERIAL;		
Minimal		V001954
GDNF IMMUNOSTAINING;		
Minimal		V002615
Mild		V002611
Moderate		V001954
Meninges; FIBROSIS; Focal;		
Minimal	V000909	
CD F-RT;		
Examined	(3)	(5)
Within Normal Limits	V002604<br>V002606	
CATHETER TRACK (CT)	V000909	V001946<br>V001954<br>V002615
CT INFILTRATION, NEUTROPHIL; Focal;		
Mild	V000909	
CT FOREIGN BODY REACTION; Focal;		
Mild	V000909	

Pathology - Histo Pathology Observations - Animal Cross Reference
15-RS-288 - 40-Week Toxicity Stud of Recombinant-Methionyl Human Glial Cell Line-Derived Neurotrophic Factor (r-metHuGDNF) via Intermittent Bilateral Intraputamenal Convection-Enhanced Delivery in Rhesus Monkeys with a 12-Week Recovery Period

Removal Reason: Terminal Sacrifice

	---- MALES -----	
	MC 0 µg	MG 87.1 µg
CD F-RT; (continued)		
CT FOREIGN BODY REACTION; Multifocal;		
Minimal		V002615
CT FIBROSIS;		
Minimal	V000909	V001954<br>V002615
CT HEMORRHAGE; Focal;		
Minimal	V000909	
CT GLIOSIS/ASTROCYTOSIS;		
Minimal		V001954<br>V002615
Mild	V000909	
CT INFILTRATION, MONONUCLEAR CELL; Multifocal;		
Minimal	V000909	V001954
CT VACUOLATED MACROPHAGES; Focal;		
Minimal		V002615
CT VACUOLATED MACROPHAGES; Multifocal;		
Mild	V000909	
CT PIGMENTED MACROPHAGES; Multifocal;		
Minimal		V001954
Mild		V002615
CT INFILTRATION, EOSINOPHIL; Multifocal;		
Minimal		V001954
CT VACUOLATION, WHITE MATTER; Focal;		
Minimal	V000909	

Pathology - Histo Pathology Observations - Animal Cross Reference
15-RS-288 - 40-Week Toxicity Stud of Recombinant-Methionyl Human Glial Cell Line-Derived Neurotrophic Factor (r-metHuGDNF) via Intermittent Bilateral Intraputamenal Convection-Enhanced Delivery in Rhesus Monkeys with a 12-Week Recovery Period

Removal Reason: Terminal Sacrifice

	MALES	
	MC 0 µg	MG 87.1 µg
CD F-RT; (continued)		
CT MINERALIZED MATERIAL;		
Minimal		V001946<br>V001954
Mild		V002615
GDNF IMMUNOSTAINING;		
Mild		V002611
Moderate		V001954<br>V002593<br>V002615
Meninges; FIBROSIS; Focal;		
Minimal		V002615
CD/PUT G-LT;		
Examined	(3)	(5)
Within Normal Limits	V002604	
CATHETER TRACK (CT)	V000909<br>V002606	V001946<br>V001954<br>V002593<br>V002611<br>V002615
CT VACUOLATED MACROPHAGES; Multifocal;		
Mild	V000909	
CT PIGMENTED MACROPHAGES; Multifocal;		
Minimal	V000909	V001954
Moderate		V002611
CT INFILTRATION, MONONUCLEAR CELL; Multifocal;		
Minimal	V000909	

Pathology - Histo Pathology Observations - Animal Cross Reference
15-RS-288 - 40-Week Toxicity Stud of Recombinant-Methionyl Human Glial Cell Line-Derived Neurotrophic Factor (r-metHuGDNF) via Intermittent Bilateral Intraputamenal Convection-Enhanced Delivery in Rhesus Monkeys with a 12-Week Recovery Period

Removal Reason: Terminal Sacrifice

	MALES	
	MC 0 µg	MG 87.1 µg
CD/PUT G-LT; (continued)		
CT GLIOSIS/ASTROCYTOSIS;		
Minimal		V001954
Mild	V000909	V002611
CT FIBROSIS;		
Minimal	V000909	
Moderate		V002611
CT INFILTRATION, EOSINOPHIL; Multifocal;		
Minimal		V001954
CT FOREIGN BODY REACTION; Focal;		
Minimal		V001954
CT FOREIGN BODY REACTION; Multifocal;		
Minimal		V002593<br>V002611
CT VACUOLATION, WHITE MATTER; Focal;		
Minimal		V001954
CT MINERALIZED MATERIAL;		
Minimal		V001954<br>V002593<br>V002611
Mild	V002606	V001946
GDNF IMMUNOSTAINING;		
Minimal		V002615
Mild		V002611
Moderate		V001954<br>V002593

Pathology - Histo Pathology Observations - Animal Cross Reference
15-RS-288 - 40-Week Toxicity Stud of Recombinant-Methionyl Human Glial Cell Line-Derived Neurotrophic Factor (r-metHuGDNF) via Intermittent Bilateral Intraputamenal Convection-Enhanced Delivery in Rhesus Monkeys with a 12-Week Recovery Period

Removal Reason: Terminal Sacrifice

	MALES	
	MC 0 µg	MG 87.1 µg
CD/PUT G-LT; (continued)		
Meninges; FIBROSIS; Focal;		
Minimal	V000909	
CD/PUT G-RT;		
Examined	(3)	(5)
Within Normal Limits	V002604	
CATHETER TRACK (CT)	V000909<br>V002606	V001946<br>V001954<br>V002593<br>V002611<br>V002615
CT FIBROSIS;		
Minimal		V001954<br>V002593<br>V002615
Moderate	V000909	V002611
CT FOREIGN BODY REACTION; Focal;		
Minimal	V000909<br>V002606	V001946<br>V001954
CT FOREIGN BODY REACTION; Multifocal;		
Minimal		V002593<br>V002615
Mild		V002611
CT GLIOSIS/ASTROCYTOSIS;		
Minimal		V001954<br>V002615
Mild	V000909	V002611

Pathology - Histo Pathology Observations - Animal Cross Reference
15-RS-288 - 40-Week Toxicity Stud of Recombinant-Methionyl Human Glial Cell Line-Derived Neurotrophic Factor (r-metHuGDNF) via Intermittent Bilateral Intraputamenal Convection-Enhanced Delivery in Rhesus Monkeys with a 12-Week Recovery Period

Removal Reason: Terminal Sacrifice

	---- MALES -----	
	MC 0 µg	MG 87.1 µg
CD/PUT G-RT; (continued)		
CT INFILTRATION, MONONUCLEAR CELL; Focal;		
Minimal		V002615
CT INFILTRATION, MONONUCLEAR CELL; Multifocal;		
Minimal		V001954<br>V002593
Mild	V000909	
CT INFILTRATION, NEUTROPHIL; Focal;		
Mild	V000909	
CT VACUOLATED MACROPHAGES; Multifocal;		
Mild	V000909	
CT PIGMENTED MACROPHAGES; Multifocal;		
Minimal	V000909	V001946<br>V001954<br>V002593<br>V002615
Mild		V002611
CT VACUOLATION, WHITE MATTER; Multifocal;		
Minimal	V000909	
CT INFILTRATION, EOSINOPHIL; Multifocal;		
Minimal		V001954<br>V002593
CT MINERALIZED MATERIAL;		
Minimal		V001946<br>V001954
Mild		V002611

Pathology - Histo Pathology Observations - Animal Cross Reference
15-RS-288 - 40-Week Toxicity Stud of Recombinant-Methionyl Human Glial Cell Line-Derived Neurotrophic Factor (r-metHuGDNF) via Intermittent Bilateral Intraputamenal Convection-Enhanced Delivery in Rhesus Monkeys with a 12-Week Recovery Period

Removal Reason: Terminal Sacrifice

	MALES	
	MC 0 µg	MG 87.1 µg
CD/PUT G-RT; (continued)		
GDNF IMMUNOSTAINING;		
Mild		V001946<br>V002611
Moderate		V001954<br>V002615
Marked		V002593
Meninges; FIBROSIS; Focal;		
Minimal	V000909	
CD/PUT H-LT;		
Examined	(3)	(5)
Within Normal Limits	V002604	
CATHETER TRACK (CT)	V000909<br>V002606	V001946<br>V001954<br>V002593<br>V002611<br>V002615
CT FIBROSIS;		
Minimal	V000909<br>V002606	V002593<br>V002615
Moderate		V002611
CT GLIOSIS/ASTROCYTOSIS;		
Minimal	V000909<br>V002606	V001954<br>V002615
Moderate		V002611
CT INFILTRATION, MONONUCLEAR CELL; Multifocal;		
Minimal	V000909	V001946<br>V002593<br>V002611

Pathology - Histo Pathology Observations - Animal Cross Reference
15-RS-288 - 40-Week Toxicity Stud of Recombinant-Methionyl Human Glial Cell Line-Derived Neurotrophic Factor (r-metHuGDNF) via Intermittent Bilateral Intraputamenal Convection-Enhanced Delivery in Rhesus Monkeys with a 12-Week Recovery Period

Removal Reason: Terminal Sacrifice

	MALES	
	MC<br>0 µg	MG<br>87.1 µg
CD/PUT H-LT; (continued)		
CT PIGMENTED MACROPHAGES; Focal;		
Minimal	V002606	
CT PIGMENTED MACROPHAGES; Multifocal;		
Minimal	V000909	V001946
Mild		V001954<br>V002593
Moderate		V002611
CT VACUOLATED MACROPHAGES; Multifocal;		
Mild	V000909	V002611
CT INFILTRATION, EOSINOPHIL; Multifocal;		
Minimal		V001954
Mild		V002593
CT FOREIGN BODY REACTION; Focal;		
Minimal		V001954
CT FOREIGN BODY REACTION; Multifocal;		
Minimal		V002593<br>V002611<br>V002615
CT VACUOLATION, WHITE MATTER; Multifocal;		
Minimal		V002611
CT PERIVASCULAR CUFFS; Mixed; Multifocal;		
Minimal		V002593
CT MINERALIZED MATERIAL;		
Minimal		V001946<br>V001954<br>V002611

Pathology - Histo Pathology Observations - Animal Cross Reference
15-RS-288 - 40-Week Toxicity Stud of Recombinant-Methionyl Human Glial Cell Line-Derived Neurotrophic Factor (r-metHuGDNF) via Intermittent Bilateral Intraputamenal Convection-Enhanced Delivery in Rhesus Monkeys with a 12-Week Recovery Period

Removal Reason: Terminal Sacrifice

	MALES	
	MC 0 µg	MG 87.1 µg
CD/PUT H-LT; (continued)		
GDNF IMMUNOSTAINING;		
Moderate		V001954<br>V002611<br>V002615
Marked		V002593
Meninges; INFILTRATION; Mononuclear cell; Focal;		
Minimal		V002611<br>V002615
CD/PUT H-RT;		
Examined	(3)	(5)
Within Normal Limits	V002604	
CATHETER TRACK (CT)	V000909<br>V002606	V001946<br>V001954<br>V002593<br>V002611<br>V002615
CT FIBROSIS;		
Minimal	V002606	
Mild		V002593<br>V002611
Moderate	V000909	V002615
CT GLIOSIS/ASTROCYTOSIS;		
Minimal		V001954<br>V002615
Mild	V000909	V002611
CT INFILTRATION, MONONUCLEAR CELL; Focal;		
Minimal		V002615

Pathology - Histo Pathology Observations - Animal Cross Reference
15-RS-288 - 40-Week Toxicity Stud of Recombinant-Methionyl Human Glial Cell Line-Derived Neurotrophic Factor (r-metHuGDNF) via Intermittent Bilateral Intraputamenal Convection-Enhanced Delivery in Rhesus Monkeys with a 12-Week Recovery Period

Removal Reason: Terminal Sacrifice

	---- MALES -----	
	MC 0 µg	MG 87.1 µg
CD/PUT H-RT; (continued)		
CT INFILTRATION, MONONUCLEAR CELL; Multifocal;		
Minimal		V001946<br>V002593
Mild	V000909	
CT INFILTRATION, NEUTROPHIL; Focal;		
Moderate	V000909	
CT PIGMENTED MACROPHAGES; Multifocal;		
Minimal	V000909	V001946<br>V001954<br>V002615
Mild		V002593<br>V002611
CT VACUOLATED MACROPHAGES; Multifocal;		
Minimal		V002611
Mild	V000909	
CT PERIVASCULAR CUFFS; Mixed; Multifocal;		
Minimal		V002593
Mild	V000909	
CT VACUOLATION, WHITE MATTER; Focal;		
Minimal		V001954
CT VACUOLATION, WHITE MATTER; Multifocal;		
Mild	V000909	
CT FOREIGN BODY REACTION; Focal;		
Minimal		V001946<br>V001954

Pathology - Histo Pathology Observations - Animal Cross Reference
15-RS-288 - 40-Week Toxicity Stud of Recombinant-Methionyl Human Glial Cell Line-Derived Neurotrophic Factor (r-metHuGDNF) via Intermittent Bilateral Intraputamenal Convection-Enhanced Delivery in Rhesus Monkeys with a 12-Week Recovery Period

Removal Reason: Terminal Sacrifice

	---- MALES -----	
	MC 0 µg	MG 87.1 µg
CD/PUT H-RT; (continued)		
CT FOREIGN BODY REACTION; Multifocal;		
Minimal		V002615
CT INFILTRATION, EOSINOPHIL; Multifocal;		
Minimal		V001954<br>V002593<br>V002611<br>V002615
CT MINERALIZED MATERIAL;		
Minimal	V002606	V001946<br>V001954
GDNF IMMUNOSTAINING;		
Moderate		V001946<br>V001954<br>V002593<br>V002611<br>V002615
CD/PUT/AC I-LT;		
Examined	(3)	(5)
CATHETER TRACK (CT)	V000909<br>V002604<br>V002606	V001946<br>V001954<br>V002611<br>V002615
CT FIBROSIS;		
Minimal	V002606	
Mild	V002604	V001954<br>V002611
CT PERIVASCULAR CUFFS; Mixed; Focal;		
Minimal		V002611

Pathology - Histo Pathology Observations - Animal Cross Reference
15-RS-288 - 40-Week Toxicity Stud of Recombinant-Methionyl Human Glial Cell Line-Derived Neurotrophic Factor (r-metHuGDNF) via Intermittent Bilateral Intraputamenal Convection-Enhanced Delivery in Rhesus Monkeys with a 12-Week Recovery Period

Removal Reason: Terminal Sacrifice

	MALES MC 0 µg	MALES MG 87.1 µg
CD/PUT/AC I-LT; (continued)		
CT PERIVASCULAR CUFFS; Mixed; Multifocal;		
Minimal	V002604	
Mild		V001954
CT PIGMENTED MACROPHAGES; Focal;		
Minimal		V002615
CT PIGMENTED MACROPHAGES; Multifocal;		
Minimal	V002604<br>V002606	V001946
Mild		V002611
CT FOREIGN BODY REACTION; Focal;		
Minimal	V002604	V001946<br>V001954
CT FOREIGN BODY REACTION; Multifocal;		
Minimal		V002611
CT VACUOLATION, WHITE MATTER; Focal;		
Minimal	V002604<br>V002606	V001946<br>V002611
CT INFILTRATION, MONONUCLEAR CELL; Focal;		
Minimal	V002606	V002615
CT INFILTRATION, MONONUCLEAR CELL; Multifocal;		
Minimal	V002604	V001946<br>V002611
CT INFILTRATION, EOSINOPHIL; Focal;		
Minimal		V001946<br>V002615

Pathology - Histo Pathology Observations - Animal Cross Reference
15-RS-288 - 40-Week Toxicity Stud of Recombinant-Methionyl Human Glial Cell Line-Derived Neurotrophic Factor (r-metHuGDNF) via Intermittent Bilateral Intraputamenal Convection-Enhanced Delivery in Rhesus Monkeys with a 12-Week Recovery Period

Removal Reason: Terminal Sacrifice

	MALES	
	MC 0 µg	MG 87.1 µg
CD/PUT/AC I-LT; (continued)		
CT INFILTRATION, EOSINOPHIL; Multifocal;		
Minimal	V002604<br>V002606	V001954<br>V002611
CT GLIOSIS/ASTROCYTOSIS;		
Minimal	V002604	V001946<br>V002615
Mild		V002611
CT VACUOLATED MACROPHAGES; Multifocal;		
Minimal		V002611
CT MINERALIZED MATERIAL;		
Minimal	V000909	V001946
GDNF IMMUNOSTAINING;		
Mild		V001946
Moderate		V001954<br>V002593<br>V002611<br>V002615
Meninges; INFILTRATION; Mixed; Focal;		
Minimal		V001954
CD/PUT/GP J-LT;		
Examined	(3)	(5)
Within Normal Limits	V000909<br>V002604<br>V002606	V002615
CATHETER TRACK (CT)		V001946
CT FOREIGN BODY REACTION; Focal;		
Minimal		V001946

Pathology - Histo Pathology Observations - Animal Cross Reference
15-RS-288 - 40-Week Toxicity Stud of Recombinant-Methionyl Human Glial Cell Line-Derived Neurotrophic Factor (r-metHuGDNF) via Intermittent Bilateral Intraputamenal Convection-Enhanced Delivery in Rhesus Monkeys with a 12-Week Recovery Period

Removal Reason: Terminal Sacrifice

	---- MALES -----	
	MC 0 µg	MG 87.1 µg
CD/PUT/GP J-LT; (continued)		
CT PIGMENTED MACROPHAGES; Multifocal;		
Minimal		V001946
CT FIBROSIS; Focal;		
Mild		V001946
CT INFILTRATION, MONONUCLEAR CELL; Multifocal;		
Mild		V001946
CT HEMORRHAGE; Focal;		
Minimal		V001946
CT MINERALIZED MATERIAL;		
Minimal		V001946
GDNF IMMUNOSTAINING;		
Minimal		V001946
Mild		V002593<br>V002611
Moderate		V001954
CD/PUT/GP J-RT;		
Examined	(3)	(5)
Within Normal Limits	V002604	V001954<br>V002615
CATHETER TRACK (CT)	V000909<br>V002606	V001946
CT GLIOSIS/ASTROCYTOSIS;		
Minimal	V000909	
CT INFILTRATION, MONONUCLEAR CELL; Multifocal;		
Minimal	V000909	V001946

Pathology - Histo Pathology Observations - Animal Cross Reference
15-RS-288 - 40-Week Toxicity Stud of Recombinant-Methionyl Human Glial Cell Line-Derived Neurotrophic Factor (r-metHuGDNF) via Intermittent Bilateral Intraputamenal Convection-Enhanced Delivery in Rhesus Monkeys with a 12-Week Recovery Period

Removal Reason: Terminal Sacrifice

	---- MALES -----	
	MC 0 µg	MG 87.1 µg
CD/PUT/GP J-RT; (continued)		
CT PERIVASCULAR CUFFS; Mixed; Multifocal;		
Mild	V000909	
CT VACUOLATION, WHITE MATTER; Multifocal;		
Mild	V000909	
CT FIBROSIS;		
Mild	V002606	
CT FOREIGN BODY REACTION; Focal;		
Minimal	V002606	V001946
CT PIGMENTED MACROPHAGES; Multifocal;		
Minimal		V001946
CT VACUOLATION, GRAY MATTER; Focal;		
Mild	V000909	
GDNF IMMUNOSTAINING;		
Mild		V002593<br>V002611
Moderate		V001946
PUT/GP K-LT;		
Examined	(3)	(5)
Within Normal Limits	V000909<br>V002604<br>V002606	V001946<br>V001954<br>V002615
GDNF IMMUNOSTAINING;		
Mild		V002593
Meninges; INFILTRATION; Mononuclear cell; Focal;		
Minimal		V002593

Pathology - Histo Pathology Observations - Animal Cross Reference
15-RS-288 - 40-Week Toxicity Stud of Recombinant-Methionyl Human Glial Cell Line-Derived Neurotrophic Factor (r-metHuGDNF) via Intermittent Bilateral Intraputamenal Convection-Enhanced Delivery in Rhesus Monkeys with a 12-Week Recovery Period

Removal Reason: Terminal Sacrifice

	MALES MC 0 µg	MALES MG 87.1 µg
PUT/GP K-LT; (continued)		
Meninges; FIBROSIS; Focal;		
Mild		V002611
PUT/GP K-RT;		
Examined	(3)	(5)
Within Normal Limits	V002604<br>V002606	V002611
AXON SPHEROIDS; Focal;		
Minimal		V001954
GDNF IMMUNOSTAINING;		
Mild		V001946
White matter; VACUOLATION; Multifocal;		
Minimal	V000909	
Choroid plexus; INFILTRATION; Mononuclear cell; Focal;		
Minimal		V002593<br>V002615
SN N;		
Examined	(3)	(5)
Within Normal Limits	V000909<br>V002604<br>V002606	V001946<br>V001954<br>V002593<br>V002611<br>V002615
THAL/SN M;		
Examined	(3)	(5)
Within Normal Limits	V000909<br>V002604<br>V002606	V001946<br>V002593<br>V002615
AXON SPHEROIDS; Focal;		
Minimal		V001954

Pathology - Histo Pathology Observations - Animal Cross Reference
15-RS-288 - 40-Week Toxicity Stud of Recombinant-Methionyl Human Glial Cell Line-Derived Neurotrophic Factor (r-metHuGDNF) via Intermittent Bilateral Intraputamenal Convection-Enhanced Delivery in Rhesus Monkeys with a 12-Week Recovery Period

Removal Reason: Terminal Sacrifice

	---- MALES -----	
	MC 0 µg	MG 87.1 µg
THAL/SN M; (continued)		
GDNF IMMUNOSTAINING;		
Minimal		V002611
Mild		V001954
THAL/STN L;		
Examined	(3)	(5)
Within Normal Limits	V002606	V001946<br>V001954<br>V002611<br>V002615
White matter; VACUOLATION; Bilateral;		
Minimal	V002604	
White matter; VACUOLATION; Focal;		
Minimal	V000909	
Meninges; INFILTRATION; Mononuclear cell; Focal;		
Minimal		V002593
THAL/STN L1-LT;		
Examined	(1)	(0)
Meninges; INFILTRATION; Mononuclear cell; Focal;		
Minimal	V000909	
SPINAL CORD, CERVICAL;		
Examined	(3)	(5)
Within Normal Limits	V000909<br>V002604<br>V002606	V001946<br>V001954<br>V002593<br>V002611<br>V002615
SPINAL CORD, THORACIC;		
Examined	(3)	(5)
Within Normal Limits	V000909	V001946

Pathology - Histo Pathology Observations - Animal Cross Reference
15-RS-288 - 40-Week Toxicity Stud of Recombinant-Methionyl Human Glial Cell Line-Derived Neurotrophic Factor (r-metHuGDNF) via Intermittent Bilateral Intraputamenal Convection-Enhanced Delivery in Rhesus Monkeys with a 12-Week Recovery Period

Removal Reason: Terminal Sacrifice

	---- MALES -----	
	MC<br>0 µg	MG<br>87.1 µg
SPINAL CORD, THORACIC; (continued)		
Within Normal Limits (continued)	V002604<br>V002606	V001954<br>V002593<br>V002611<br>V002615
SPINAL CORD, LUMBAR;		
Examined	(3)	(5)
Within Normal Limits	V000909<br>V002604<br>V002606	V001946<br>V001954<br>V002593<br>V002611<br>V002615
DRG, CERVICAL;		
Examined	(3)	(5)
Within Normal Limits	V000909<br>V002604<br>V002606	V001946<br>V001954<br>V002593<br>V002611
Ganglion cell; VACUOLATION;		
Minimal		V002615
DRG, THORACIC;		
Examined	(3)	(5)
Within Normal Limits	V000909<br>V002604	V001946<br>V002611<br>V002615
MINERALIZATION;		
Minimal		V002593
Ganglion cell; VACUOLATION;		
Minimal	V002606	
Mild		V001954

Pathology - Histo Pathology Observations - Animal Cross Reference
15-RS-288 - 40-Week Toxicity Stud of Recombinant-Methionyl Human Glial Cell Line-Derived Neurotrophic Factor (r-metHuGDNF) via Intermittent Bilateral Intraputamenal Convection-Enhanced Delivery in Rhesus Monkeys with a 12-Week Recovery Period

Removal Reason: Terminal Sacrifice

	---- MALES -----	
	MC 0 µg	MG 87.1 µg
DRG, LUMBAR;		
Examined	(3)	(5)
Within Normal Limits	V000909<br>V002604<br>V002606	V001946<br>V001954<br>V002611<br>V002615
Ganglion cell; VACUOLATION;		
Minimal		V002593
TRIGEMINAL GANGLIA;		
Examined	(3)	(5)
Within Normal Limits	V000909<br>V002606	V001946<br>V001954<br>V002593<br>V002611<br>V002615
MINERALIZATION;		
Minimal	V002604	

Pathology - Histo Pathology Observations - Animal Cross Reference
15-RS-288 - 40-Week Toxicity Stud of Recombinant-Methionyl Human Glial Cell Line-Derived Neurotrophic Factor (r-metHuGDNF) via Intermittent Bilateral Intraputamenal Convection-Enhanced Delivery in Rhesus Monkeys with a 12-Week Recovery Period

---

End Of Print

Table 1-3B. Histopathology Cross Reference Table —Recovery

PTA010-05/00 Provantis Version 9.1 Date: 11/24/2015 9:43 Page: 1

Pathology - Histo Pathology Observations - Animal Cross Reference
15-RS-288 - 40-Week Toxicity Stud of Recombinant-Methionyl Human Glial Cell Line-Derived Neurotrophic Factor (r-metHuGDNF) via Intermittent Bilateral Intraputamenal Convection-Enhanced Delivery in Rhesus Monkeys with a 12-Week Recovery Period

Removal Reason: Recovery Sacrifice

	---- MALES -----	
	MC 0 µg	MG 87.1 µg
**BRAIN D;**		
Examined	(3)	(5)
Within Normal Limits	V002591<br>V002613	V001935<br>V002047<br>V002608<br>V002610
Meninges; FIBROSIS; Focal; Minimal	V002597	
Meninges; INFILTRATION; Mononuclear cell; Multifocal; Minimal	V002597	V002614
**BS/PONS N-LT;**		
Examined	(0)	(1)
Within Normal Limits		V002047
**BS/PONS N-RT;**		
Examined	(0)	(1)
Within Normal Limits		V002047
**BS/PONS O-LT;**		
Examined	(3)	(5)
Within Normal Limits	V002591<br>V002597<br>V002613	V001935<br>V002608<br>V002610<br>V002614
**BS/PONS O-RT;**		
Examined	(3)	(5)
Within Normal Limits	V002591<br>V002597<br>V002613	V001935<br>V002047<br>V002608<br>V002610<br>V002614
**CB/MO T-CB;**		
Examined	(0)	(1)

Pathology - Histo Pathology Observations - Animal Cross Reference
15-RS-288 - 40-Week Toxicity Stud of Recombinant-Methionyl Human Glial Cell Line-Derived Neurotrophic Factor (r-metHuGDNF) via Intermittent Bilateral Intraputamenal Convection-Enhanced Delivery in Rhesus Monkeys with a 12-Week Recovery Period

Removal Reason: Recovery Sacrifice

	MALES	
	MC 0 µg	MG 87.1 µg
**CB/MO T-CB; (continued)**		
Meninges; INFILTRATION; Mononuclear cell; Focal; Minimal		V002610
Perivascular; INFILTRATION; Mononuclear cell; Focal; Minimal		V002610
**CB/MO T-LT;**		
Examined	(0)	(1)
Within Normal Limits		V002610
**CB/MO T-RT;**		
Examined	(0)	(1)
Within Normal Limits		V002610
**CB/MO U-CB;**		
Examined	(3)	(4)
Within Normal Limits	V002591<br>V002597<br>V002613	V001935<br>V002047<br>V002608<br>V002614
**CB/MO U-LT;**		
Examined	(3)	(4)
Within Normal Limits	V002591<br>V002597<br>V002613	V001935<br>V002047<br>V002608<br>V002614
**CB/MO U-RT;**		
Examined	(3)	(4)
Within Normal Limits	V002591<br>V002597<br>V002613	V001935<br>V002608<br>V002614
Meninges; FIBROSIS; Focal; Mild		V002047

Pathology - Histo Pathology Observations - Animal Cross Reference
15-RS-288 - 40-Week Toxicity Stud of Recombinant-Methionyl Human Glial Cell Line-Derived Neurotrophic Factor (r-metHuGDNF) via Intermittent Bilateral Intraputamenal Convection-Enhanced Delivery in Rhesus Monkeys with a 12-Week Recovery Period

Removal Reason: Recovery Sacrifice

	MALES	
	MC 0 µg	MG 87.1 µg
CB R-CB;		
Examined	(3)	(5)
Within Normal Limits	V002591<br>V002597<br>V002613	V001935<br>V002047<br>V002608<br>V002614
Axon; DEGENERATION; Focal;		
Minimal		V002610
CB V-CB;		
Examined	(3)	(5)
Within Normal Limits	V002591<br>V002597<br>V002613	V001935<br>V002047<br>V002608<br>V002610<br>V002614
CD F-LT;		
Examined	(1)	(1)
CATHETER TRACK (CT)	V002597	V002047
CT PIGMENTED MACROPHAGES; Focal;		
Minimal		V002047
CT INFILTRATION, MONONUCLEAR CELL; Focal;		
Minimal		V002047
CT INFILTRATION, MONONUCLEAR CELL; Multifocal;		
Minimal	V002597	
CT FIBROSIS; Focal;		
Moderate		V002047
CT INFILTRATION, EOSINOPHIL; Focal;		
Minimal		V002047

Pathology - Histo Pathology Observations - Animal Cross Reference
15-RS-288 - 40-Week Toxicity Stud of Recombinant-Methionyl Human Glial Cell Line-Derived Neurotrophic Factor (r-metHuGDNF) via Intermittent Bilateral Intraputamenal Convection-Enhanced Delivery in Rhesus Monkeys with a 12-Week Recovery Period

Removal Reason: Recovery Sacrifice

	MALES	
	MC 0 µg	MG 87.1 µg
CD F-LT; (continued)		
CT INFILTRATION, EOSINOPHIL; Multifocal; Minimal	V002597	
CT FOREIGN BODY REACTION; Multifocal; Minimal	V002597	V002047
CT PERIVASCULAR CUFFS; Mononuclear cell; Focal; Minimal	V002597	
CT VACUOLATION, GRAY MATTER; Focal; Minimal		V002047
CT MINERALIZED MATERIAL; Minimal		V002047
GDNF IMMUNOSTAINING; Mild		V002047
Meninges; FIBROSIS; Focal; Minimal		V002047
CD F-RT; Examined	(1)	(1)
CATHETER TRACK (CT)	V002597	
CT FOREIGN BODY REACTION; Multifocal; Minimal	V002597	
CT INFILTRATION, MONONUCLEAR CELL; Multifocal; Minimal	V002597	
CT INFILTRATION, EOSINOPHIL; Multifocal; Minimal	V002597	
CT MINERALIZED MATERIAL; Minimal	V002597	

Pathology - Histo Pathology Observations - Animal Cross Reference
15-RS-288 - 40-Week Toxicity Stud of Recombinant-Methionyl Human Glial Cell Line-Derived Neurotrophic Factor (r-metHuGDNF) via Intermittent Bilateral Intraputamenal Convection-Enhanced Delivery in Rhesus Monkeys with a 12-Week Recovery Period

Removal Reason: Recovery Sacrifice

	MALES	
	MC 0 µg	MG 87.1 µg
CD F-RT; (continued)		
Meninges; FIBROSIS; Focal;		
Minimal		V002047
CD G-LT;		
Examined	(0)	(3)
Within Normal Limits		V001935
CATHETER TRACK (CT)		V002608<br>V002610
CT PIGMENTED MACROPHAGES; Multifocal;		
Minimal		V002610
CT FOREIGN BODY REACTION; Focal;		
Minimal		V002610
CT FOREIGN BODY REACTION; Multifocal;		
Minimal		V002608
CT GLIOSIS/ASTROCYTOSIS;		
Mild		V002610
CT INFILTRATION, NEUTROPHIL; Diffuse;		
Mild		V002608
CT INFILTRATION, EOSINOPHIL; Multifocal;		
Minimal		V002608
CT INFILTRATION, MONONUCLEAR CELL; Multifocal;		
Minimal		V002608
CT PERIVASCULAR CUFFS; Mononuclear cell; Multifocal;		
Minimal		V002608
CT VACUOLATION, WHITE MATTER; Focal;		
Minimal		V002608

Pathology - Histo Pathology Observations - Animal Cross Reference
15-RS-288 - 40-Week Toxicity Stud of Recombinant-Methionyl Human Glial Cell Line-Derived Neurotrophic Factor (r-metHuGDNF) via Intermittent Bilateral Intraputamenal Convection-Enhanced Delivery in Rhesus Monkeys with a 12-Week Recovery Period

Removal Reason: Recovery Sacrifice

	---- MALES -----	
	MC 0 µg	MG 87.1 µg
CD G-LT; (continued)		
CT FIBROSIS; Focal;		
Minimal		V002608
CT VACUOLATED MACROPHAGES; Multifocal;		
Minimal		V002608
CT MINERALIZED MATERIAL;		
Minimal		V002610
GDNF IMMUNOSTAINING;		
Minimal		V002610
Mild		V002608
CD G-RT;		
Examined	(0)	(3)
Within Normal Limits		V001935
CATHETER TRACK (CT)		V002608<br>V002610
CT FOREIGN BODY REACTION; Focal;		
Minimal		V002610
CT FOREIGN BODY REACTION; Multifocal;		
Mild		V002608
CT PIGMENTED MACROPHAGES; Multifocal;		
Minimal		V002608<br>V002610
CT GLIOSIS/ASTROCYTOSIS;		
Minimal		V002608<br>V002610
CT FIBROSIS; Diffuse;		
Moderate		V002608

Pathology - Histo Pathology Observations - Animal Cross Reference
15-RS-288 - 40-Week Toxicity Stud of Recombinant-Methionyl Human Glial Cell Line-Derived Neurotrophic Factor (r-metHuGDNF) via Intermittent Bilateral Intraputamenal Convection-Enhanced Delivery in Rhesus Monkeys with a 12-Week Recovery Period

Removal Reason: Recovery Sacrifice

	---- MALES -----	
	MC 0 µg	MG 87.1 µg
CD G-RT; (continued)		
CT INFILTRATION, NEUTROPHIL; Diffuse;		
Moderate		V002608
CT INFILTRATION, MONONUCLEAR CELL; Multifocal;		
Mild		V002608
CT VACUOLATED MACROPHAGES; Multifocal;		
Mild		V002608
CT VACUOLATION, WHITE MATTER; Focal;		
Mild		V002608
GDNF IMMUNOSTAINING;		
Minimal		V002608
Mild		V002610
CD/PUT G-LT;		
Examined	(3)	(2)
Within Normal Limits	V002591	V002047
CATHETER TRACK (CT)	V002597<br>V002613	V002614
CT PIGMENTED MACROPHAGES; Focal;		
Minimal	V002613	
CT PIGMENTED MACROPHAGES; Multifocal;		
Minimal	V002597	
CT INFILTRATION, MONONUCLEAR CELL; Multifocal;		
Minimal	V002597	
CT GLIOSIS/ASTROCYTOSIS;		
Minimal	V002597	

Pathology - Histo Pathology Observations - Animal Cross Reference
15-RS-288 - 40-Week Toxicity Stud of Recombinant-Methionyl Human Glial Cell Line-Derived Neurotrophic Factor (r-metHuGDNF) via Intermittent Bilateral Intraputamenal Convection-Enhanced Delivery in Rhesus Monkeys with a 12-Week Recovery Period

Removal Reason: Recovery Sacrifice

	---- MALES -----	
	MC 0 µg	MG 87.1 µg
CD/PUT G-LT; (continued)		
CT FIBROSIS;		
Minimal	V002597	
Mild		V002614
CT INFILTRATION, EOSINOPHIL; Multifocal;		
Minimal	V002597	
CT FOREIGN BODY REACTION; Focal;		
Minimal		V002614
CT FOREIGN BODY REACTION; Multifocal;		
Minimal	V002597	
CT VACUOLATION, WHITE MATTER; Focal;		
Minimal	V002597	
CT PERIVASCULAR CUFFS; Mononuclear cell; Multifocal;		
Minimal	V002597	
CT MINERALIZED MATERIAL;		
Minimal		V002614
GDNF IMMUNOSTAINING;		
Minimal		V002614
CD/PUT G-RT;		
Examined	(3)	(2)
Within Normal Limits	V002591	V002047<br>V002614
CATHETER TRACK (CT)	V002597<br>V002613	
CT FOREIGN BODY REACTION; Multifocal;		
Minimal	V002597	

Pathology - Histo Pathology Observations - Animal Cross Reference
15-RS-288 - 40-Week Toxicity Stud of Recombinant-Methionyl Human Glial Cell Line-Derived Neurotrophic Factor (r-metHuGDNF) via Intermittent Bilateral Intraputamenal Convection-Enhanced Delivery in Rhesus Monkeys with a 12-Week Recovery Period

Removal Reason: Recovery Sacrifice

	MALES MC 0 µg	MALES MG 87.1 µg
CD/PUT G-RT; (continued)		
CT INFILTRATION, MONONUCLEAR CELL; Multifocal;		
Minimal	V002597	
CT PIGMENTED MACROPHAGES; Focal;		
Minimal	V002613	
CT INFILTRATION, EOSINOPHIL; Multifocal;		
Minimal	V002597	
Meninges; FIBROSIS; Focal;		
Minimal	V002597	
CD/PUT H-LT;		
Examined	(3)	(5)
Within Normal Limits	V002591	V001935
CATHETER TRACK (CT)	V002597<br>V002613	V002047<br>V002608<br>V002610<br>V002614
CT FIBROSIS;		
Minimal	V002613	V002047<br>V002614
Mild		V002608<br>V002610
Moderate	V002597	
CT GLIOSIS/ASTROCYTOSIS;		
Minimal	V002597	V002610
Mild		V002608
CT INFILTRATION, MONONUCLEAR CELL; Multifocal;		
Minimal		V002610

Pathology - Histo Pathology Observations - Animal Cross Reference
15-RS-288 - 40-Week Toxicity Stud of Recombinant-Methionyl Human Glial Cell Line-Derived Neurotrophic Factor (r-metHuGDNF) via Intermittent Bilateral Intraputamenal Convection-Enhanced Delivery in Rhesus Monkeys with a 12-Week Recovery Period

Removal Reason: Recovery Sacrifice

	MALES	
	MC 0 µg	MG 87.1 µg
CD/PUT H-LT; (continued)		
Mild	V002597	V002608
CT PIGMENTED MACROPHAGES; Focal;		
Minimal	V002613	V002614
CT PIGMENTED MACROPHAGES; Multifocal;		
Minimal		V002047<br>V002608<br>V002610
CT INFILTRATION, EOSINOPHIL; Multifocal;		
Mild		V002608
CT FOREIGN BODY REACTION; Focal;		
Minimal	V002613	V002047<br>V002614
CT FOREIGN BODY REACTION; Multifocal;		
Minimal	V002597	V002610
Mild		V002608
CT VACUOLATION, WHITE MATTER; Focal;		
Minimal		V002047
CT VACUOLATION, WHITE MATTER; Multifocal;		
Minimal	V002597	
Moderate		V002608
CT VACUOLATION, GRAY MATTER; Focal;		
Minimal		V002047
CT INFILTRATION, NEUTROPHIL; Diffuse;		
Moderate		V002608

Pathology - Histo Pathology Observations - Animal Cross Reference
15-RS-288 - 40-Week Toxicity Stud of Recombinant-Methionyl Human Glial Cell Line-Derived Neurotrophic Factor (r-metHuGDNF) via Intermittent Bilateral Intraputamenal Convection-Enhanced Delivery in Rhesus Monkeys with a 12-Week Recovery Period

Removal Reason: Recovery Sacrifice

	---- MALES -----	
	MC 0 µg	MG 87.1 µg
CD/PUT H-LT; (continued)		
CT PERIVASCULAR CUFFS; Mixed; Multifocal;		
Minimal		V002608
CT MINERALIZED MATERIAL;		
Minimal	V002597	V002614
GDNF IMMUNOSTAINING;		
Minimal		V002608<br>V002614
Mild		V002047<br>V002610
Meninges; INFILTRATION; Mononuclear cell; Focal;		
Minimal		V002608
Meninges; FIBROSIS; Focal;		
Minimal		V002614
CD/PUT H-RT;		
Examined	(3)	(5)
CATHETER TRACK (CT)	V002591<br>V002597<br>V002613	V001935<br>V002047<br>V002608<br>V002610<br>V002614
CT FIBROSIS;		
Minimal	V002591<br>V002597	V001935
Mild		V002047<br>V002610
Moderate		V002608

Pathology - Histo Pathology Observations - Animal Cross Reference
15-RS-288 - 40-Week Toxicity Stud of Recombinant-Methionyl Human Glial Cell Line-Derived Neurotrophic Factor (r-metHuGDNF) via Intermittent Bilateral Intraputamenal Convection-Enhanced Delivery in Rhesus Monkeys with a 12-Week Recovery Period

Removal Reason: Recovery Sacrifice

	MALES MC 0 µg	MALES MG 87.1 µg
CD/PUT H-RT; (continued)		
CT GLIOSIS/ASTROCYTOSIS;		
Minimal	V002597	V001935
Mild		V002047<br>V002608
CT INFILTRATION, MONONUCLEAR CELL; Focal;		
Minimal	V002591	
CT INFILTRATION, MONONUCLEAR CELL; Multifocal;		
Minimal	V002597	V002610
Mild		V002608
CT INFILTRATION, NEUTROPHIL; Diffuse;		
Moderate		V002608
CT PIGMENTED MACROPHAGES; Multifocal;		
Minimal		V001935<br>V002608<br>V002610<br>V002614
Mild		V002047
CT VACUOLATED MACROPHAGES; Multifocal;		
Mild		V002608
CT PERIVASCULAR CUFFS; Mononuclear cell; Multifocal;		
Minimal	V002597	V002047
CT VACUOLATION, WHITE MATTER; Focal;		
Minimal		V002614
CT VACUOLATION, WHITE MATTER; Multifocal;		
Mild		V002047<br>V002608

Pathology - Histo Pathology Observations - Animal Cross Reference
15-RS-288 - 40-Week Toxicity Stud of Recombinant-Methionyl Human Glial Cell Line-Derived Neurotrophic Factor (r-metHuGDNF) via Intermittent Bilateral Intraputamenal Convection-Enhanced Delivery in Rhesus Monkeys with a 12-Week Recovery Period

Removal Reason: Recovery Sacrifice

	---- MALES -----	
	MC<br>0 µg	MG<br>87.1 µg
CD/PUT H-RT; (continued)		
CT FOREIGN BODY REACTION; Focal;		
Minimal		V001935<br>V002047<br>V002614
CT FOREIGN BODY REACTION; Multifocal;		
Minimal	V002591	V002610
CT INFILTRATION, EOSINOPHIL; Focal;		
Minimal	V002591	V001935
CT INFILTRATION, EOSINOPHIL; Multifocal;		
Minimal	V002597	
CT AXON SPHEROIDS; Focal;		
Minimal		V002614
CT MINERALIZED MATERIAL;		
Minimal	V002591<br>V002613	V002047<br>V002610<br>V002614
GDNF IMMUNOSTAINING;		
Minimal		V001935
Mild		V002047<br>V002610<br>V002614
Meninges; FIBROSIS; Multifocal;		
Minimal	V002597	
Axon; DEGENERATION; Focal;		
Minimal		V002610
CD/PUT I-RT;		
Examined	(3)	(5)
Within Normal Limits	V002597	V002610

Pathology - Histo Pathology Observations - Animal Cross Reference
15-RS-288 - 40-Week Toxicity Stud of Recombinant-Methionyl Human Glial Cell Line-Derived Neurotrophic Factor (r-metHuGDNF) via Intermittent Bilateral Intraputamenal Convection-Enhanced Delivery in Rhesus Monkeys with a 12-Week Recovery Period

Removal Reason: Recovery Sacrifice

	MALES MC 0 µg	MALES MG 87.1 µg
CD/PUT I-RT; (continued)		
CATHETER TRACK (CT)	V002591<br>V002613	V001935<br>V002047<br>V002608<br>V002614
CT FOREIGN BODY REACTION; Focal; Minimal		V001935<br>V002614
CT FOREIGN BODY REACTION; Multifocal; Minimal	V002591<br>V002613	
CT PIGMENTED MACROPHAGES; Focal; Minimal	V002591	V001935<br>V002047<br>V002614
CT PIGMENTED MACROPHAGES; Multifocal; Minimal	V002613	
Mild		V002608
CT INFILTRATION, MONONUCLEAR CELL; Focal; Minimal	V002613	
CT INFILTRATION, MONONUCLEAR CELL; Multifocal; Mild		V002608
CT FIBROSIS; Focal; Minimal	V002613	
Moderate		V002608
CT FIBROSIS; Multifocal; Minimal		V001935

Pathology - Histo Pathology Observations - Animal Cross Reference
15-RS-288 - 40-Week Toxicity Stud of Recombinant-Methionyl Human Glial Cell Line-Derived Neurotrophic Factor (r-metHuGDNF) via Intermittent Bilateral Intraputamenal Convection-Enhanced Delivery in Rhesus Monkeys with a 12-Week Recovery Period

Removal Reason: Recovery Sacrifice

	MALES MC 0 µg	MALES MG 87.1 µg
CD/PUT I-RT; (continued)		
CT VACUOLATION, WHITE MATTER; Focal;		
Minimal		V002614
CT VACUOLATION, WHITE MATTER; Multifocal;		
Mild		V002608
CT GLIOSIS/ASTROCYTOSIS; Focal;		
Minimal		V002047<br>V002614
CT GLIOSIS/ASTROCYTOSIS; Multifocal;		
Minimal		V001935
Mild		V002608
CT INFILTRATION, NEUTROPHIL; Diffuse;		
Moderate		V002608
CT VACUOLATED MACROPHAGES; Multifocal;		
Minimal		V002608
CT PERIVASCULAR CUFFS; Mononuclear cell; Multifocal;		
Minimal		V002608
CT INFILTRATION, EOSINOPHIL; Focal;		
Minimal	V002591	
CT INFILTRATION, EOSINOPHIL; Multifocal;		
Minimal		V002608
CT MINERALIZED MATERIAL;		
Minimal	V002591<br>V002613	V001935<br>V002614
GDNF IMMUNOSTAINING;		
Minimal		V002614

Pathology - Histo Pathology Observations - Animal Cross Reference
15-RS-288 - 40-Week Toxicity Stud of Recombinant-Methionyl Human Glial Cell Line-Derived Neurotrophic Factor (r-metHuGDNF) via Intermittent Bilateral Intraputamenal Convection-Enhanced Delivery in Rhesus Monkeys with a 12-Week Recovery Period

Removal Reason: Recovery Sacrifice

	---- MALES -----	
	MC 0 µg	MG 87.1 µg
CD/PUT I-RT; (continued)		
Mild		V001935
Perivascular; INFILTRATION; Mononuclear cell; Focal;		
Minimal		V001935
CD/PUT/AC I-LT;		
Examined	(3)	(5)
Within Normal Limits		V002610
CATHETER TRACK (CT)	V002591<br>V002597<br>V002613	V001935<br>V002608<br>V002614
CT FIBROSIS;		
Minimal	V002591<br>V002597	
Mild		V002608<br>V002614
CT PERIVASCULAR CUFFS; Mixed; Multifocal;		
Minimal		V002608
CT PIGMENTED MACROPHAGES; Focal;		
Minimal	V002591<br>V002613	
CT PIGMENTED MACROPHAGES; Multifocal;		
Minimal	V002597	V001935<br>V002608<br>V002614
CT FOREIGN BODY REACTION; Focal;		
Minimal	V002597<br>V002613	

Pathology - Histo Pathology Observations - Animal Cross Reference
15-RS-288 - 40-Week Toxicity Stud of Recombinant-Methionyl Human Glial Cell Line-Derived Neurotrophic Factor (r-metHuGDNF) via Intermittent Bilateral Intraputamenal Convection-Enhanced Delivery in Rhesus Monkeys with a 12-Week Recovery Period

Removal Reason: Recovery Sacrifice

	---- MALES -----	
	MC<br>0 µg	MG<br>87.1 µg
CD/PUT/AC I-LT; (continued)		
CT FOREIGN BODY REACTION; Multifocal;		
Minimal	V002591	V001935<br>V002608<br>V002614
CT VACUOLATION, WHITE MATTER; Focal;		
Minimal	V002613	
CT VACUOLATION, WHITE MATTER; Multifocal;		
Minimal		V002614
Mild		V002608
CT INFILTRATION, MONONUCLEAR CELL; Focal;		
Minimal	V002597	
CT INFILTRATION, MONONUCLEAR CELL; Multifocal;		
Minimal		V001935
Mild		V002608
CT INFILTRATION, EOSINOPHIL; Multifocal;		
Minimal		V001935
CT GLIOSIS/ASTROCYTOSIS;		
Minimal	V002591<br>V002613	V002614
Mild		V002608
CT INFILTRATION, NEUTROPHIL; Diffuse;		
Moderate		V002608
CT VACUOLATED MACROPHAGES; Multifocal;		
Mild		V002608

Pathology - Histo Pathology Observations - Animal Cross Reference
15-RS-288 - 40-Week Toxicity Stud of Recombinant-Methionyl Human Glial Cell Line-Derived Neurotrophic Factor (r-metHuGDNF) via Intermittent Bilateral Intraputamenal Convection-Enhanced Delivery in Rhesus Monkeys with a 12-Week Recovery Period

Removal Reason: Recovery Sacrifice

	---- MALES -----	
	MC 0 µg	MG 87.1 µg
CD/PUT/AC I-LT; (continued)		
CT MINERALIZED MATERIAL;		
Minimal	V002591 V002597	V001935
GDNF IMMUNOSTAINING;		
Minimal		V002608 V002614
Mild		V001935
Meninges; FIBROSIS; Focal;		
Minimal		V002047
CD/PUT/GP J-LT;		
Examined	(3)	(5)
Within Normal Limits	V002597	V002047 V002610 V002614
CATHETER TRACK (CT)	V002613	V001935 V002608
CT FOREIGN BODY REACTION; Focal;		
Minimal		V001935
CT PIGMENTED MACROPHAGES; Focal;		
Minimal		V001935
CT PIGMENTED MACROPHAGES; Multifocal;		
Minimal		V002608
CT FIBROSIS; Focal;		
Minimal	V002613	V001935
Mild		V002608

Pathology - Histo Pathology Observations - Animal Cross Reference
15-RS-288 - 40-Week Toxicity Stud of Recombinant-Methionyl Human Glial Cell Line-Derived Neurotrophic Factor (r-metHuGDNF) via Intermittent Bilateral Intraputamenal Convection-Enhanced Delivery in Rhesus Monkeys with a 12-Week Recovery Period

Removal Reason: Recovery Sacrifice

	---- MALES -----	
	MC 0 µg	MG 87.1 µg
**CD/PUT/GP J-LT; (continued)**		
CT INFILTRATION, MONONUCLEAR CELL; Focal;		
Minimal		V001935
CT INFILTRATION, MONONUCLEAR CELL; Multifocal;		
Mild		V002608
CT VACUOLATION, WHITE MATTER; Multifocal;		
Minimal	V002613	
Mild		V002608
CT GLIOSIS/ASTROCYTOSIS; Multifocal;		
Minimal	V002613	V002608
CT INFILTRATION, NEUTROPHIL; Diffuse;		
Moderate		V002608
CT VACUOLATED MACROPHAGES; Focal;		
Minimal	V002613	
CT VACUOLATED MACROPHAGES; Multifocal;		
Minimal		V002608
CT PERIVASCULAR CUFFS; Mixed; Focal;		
Minimal		V002608
GDNF IMMUNOSTAINING;		
Mild		V001935<br>V002608
Perivascular; INFILTRATION; Mononuclear cell; Focal;		
Minimal	V002591	
**CD/PUT/GP J-RT;**		
Examined	(3)	(5)
Within Normal Limits	V002597<br>V002613	V002047<br>V002610

Pathology - Histo Pathology Observations - Animal Cross Reference
15-RS-288 - 40-Week Toxicity Stud of Recombinant-Methionyl Human Glial Cell Line-Derived Neurotrophic Factor (r-metHuGDNF) via Intermittent Bilateral Intraputamenal Convection-Enhanced Delivery in Rhesus Monkeys with a 12-Week Recovery Period

Removal Reason: Recovery Sacrifice

	---- MALES -----	
	MC 0 µg	MG 87.1 µg
CD/PUT/GP J-RT; (continued)		
CATHETER TRACK (CT)	V002591	V001935<br>V002608<br>V002614
CT GLIOSIS/ASTROCYTOSIS;		
Minimal	V002591	V002608
CT INFILTRATION, MONONUCLEAR CELL; Focal;		
Minimal	V002591	
CT INFILTRATION, MONONUCLEAR CELL; Multifocal;		
Mild		V002608
CT PERIVASCULAR CUFFS; Mononuclear cell; Multifocal;		
Minimal		V002608
CT VACUOLATION, WHITE MATTER; Multifocal;		
Mild		V002608
CT FIBROSIS;		
Mild	V002591	V001935<br>V002614
Moderate		V002608
CT PIGMENTED MACROPHAGES; Multifocal;		
Minimal	V002591	V001935<br>V002608<br>V002614
CT INFILTRATION, EOSINOPHIL; Focal;		
Minimal	V002591	
CT INFILTRATION, NEUTROPHIL; Diffuse;		
Moderate		V002608

Pathology - Histo Pathology Observations - Animal Cross Reference
15-RS-288 - 40-Week Toxicity Stud of Recombinant-Methionyl Human Glial Cell Line-Derived Neurotrophic Factor (r-metHuGDNF) via Intermittent Bilateral Intraputamenal Convection-Enhanced Delivery in Rhesus Monkeys with a 12-Week Recovery Period

Removal Reason: Recovery Sacrifice

	---- MALES ----- MC 0 µg	MG 87.1 µg
CD/PUT/GP J-RT; (continued)		
CT VACUOLATED MACROPHAGES; Multifocal; Mild		V002608
CT MINERALIZED MATERIAL; Minimal		V002614
GDNF IMMUNOSTAINING; Mild		V001935<br>V002614
PUT/GP K-LT;		
Examined	(2)	(4)
Within Normal Limits	V002591<br>V002613	V002610<br>V002614
AXON SPHEROIDS; Focal; Minimal		V001935
Perivascular; INFILTRATION; Mononuclear cell; Focal; Minimal		V002608
PUT/GP K-RT;		
Examined	(2)	(4)
Within Normal Limits	V002591	V001935<br>V002610<br>V002614
CATHETER TRACK (CT)		V002608
CT FIBROSIS; Focal; Mild		V002608
CT PERIVASCULAR CUFFS; Mononuclear cell; Multifocal; Minimal		V002608
CT INFILTRATION, MONONUCLEAR CELL; Multifocal; Mild		V002608

Pathology - Histo Pathology Observations - Animal Cross Reference
15-RS-288 - 40-Week Toxicity Stud of Recombinant-Methionyl Human Glial Cell Line-Derived Neurotrophic Factor (r-metHuGDNF) via Intermittent Bilateral Intraputamenal Convection-Enhanced Delivery in Rhesus Monkeys with a 12-Week Recovery Period

Removal Reason: Recovery Sacrifice

	---- MALES ----- MC 0 µg	MG 87.1 µg
PUT/GP K-RT; (continued)		
CT PIGMENTED MACROPHAGES; Multifocal;		
Minimal		V002608
CT GLIOSIS/ASTROCYTOSIS; Multifocal;		
Mild		V002608
CT VACUOLATION, GRAY MATTER; Multifocal;		
Minimal		V002608
Meninges; FIBROSIS; Multifocal;		
Minimal	V002613	
SN M;		
Examined	(0)	(2)
Within Normal Limits		V002047
SN N;		
Examined	(3)	(5)
Within Normal Limits	V002613	V001935<br>V002608<br>V002610<br>V002614
Meninges; INFILTRATION; Mononuclear cell; Focal;		
Minimal	V002597	
Meninges; FIBROSIS; Focal;		
Minimal	V002591	
SN N-RT;		
Examined	(1)	(0)
Within Normal Limits	V002597	
THAL/SN L;		
Examined	(0)	(1)
Within Normal Limits		V002047

Pathology - Histo Pathology Observations - Animal Cross Reference
15-RS-288 - 40-Week Toxicity Stud of Recombinant-Methionyl Human Glial Cell Line-Derived Neurotrophic Factor (r-metHuGDNF) via Intermittent Bilateral Intraputamenal Convection-Enhanced Delivery in Rhesus Monkeys with a 12-Week Recovery Period

Removal Reason: Recovery Sacrifice

	---- MALES -----	
	MC 0 µg	MG 87.1 µg
THAL/SN L1-LT;		
Examined	(0)	(1)
Within Normal Limits		V002610
THAL/SN M;		
Examined	(3)	(5)
Within Normal Limits	V002591<br>V002597<br>V002613	V002608<br>V002610<br>V002614
Perivascular; INFILTRATION; Mononuclear cell; Focal;		
Minimal		V001935
THAL/STN K;		
Examined	(0)	(1)
Within Normal Limits		V002047
THAL/STN L;		
Examined	(3)	(5)
Within Normal Limits	V002597<br>V002613	V002608<br>V002610<br>V002614
AXON SPHEROIDS; Focal;		
Minimal		V001935
Meninges; INFILTRATION; Mononuclear cell; Focal;		
Minimal	V002591	
THAL/STN L1-LT;		
Examined	(2)	(3)
Within Normal Limits	V002613	V001935<br>V002608
INFILTRATION; Mononuclear cell; Focal;		
Minimal	V002591	

Pathology - Histo Pathology Observations - Animal Cross Reference
15-RS-288 - 40-Week Toxicity Stud of Recombinant-Methionyl Human Glial Cell Line-Derived Neurotrophic Factor (r-metHuGDNF) via Intermittent Bilateral Intraputamenal Convection-Enhanced Delivery in Rhesus Monkeys with a 12-Week Recovery Period

Removal Reason: Recovery Sacrifice

	---- MALES -----	
	MC<br>0 µg	MG<br>87.1 µg
THAL/STN L1-LT; (continued)		
INFILTRATION; Neutrophilic; Focal;		
Minimal	V002591	
PERIVASCULAR CUFFS, MIXED CELL; Multifocal;		
Minimal	V002591	
Meninges; INFILTRATION; Mononuclear cell; Focal;		
Minimal		V002614
SPINAL CORD, CERVICAL;		
Examined	(3)	(5)
Within Normal Limits	V002591<br>V002597<br>V002613	V001935<br>V002047<br>V002608<br>V002614
Axon; DEGENERATION; Focal;		
Minimal		V002610
SPINAL CORD, THORACIC;		
Examined	(3)	(5)
Within Normal Limits	V002591<br>V002597<br>V002613	V001935<br>V002047<br>V002608<br>V002614
Axon; DEGENERATION; Focal;		
Minimal		V002610
SPINAL CORD, LUMBAR;		
Examined	(3)	(5)
Within Normal Limits	V002597<br>V002613	V001935<br>V002047<br>V002608<br>V002614

Pathology - Histo Pathology Observations - Animal Cross Reference
15-RS-288 - 40-Week Toxicity Stud of Recombinant-Methionyl Human Glial Cell Line-Derived Neurotrophic Factor (r-metHuGDNF) via Intermittent Bilateral Intraputamenal Convection-Enhanced Delivery in Rhesus Monkeys with a 12-Week Recovery Period

Removal Reason: Recovery Sacrifice

	MALES MC 0 µg	MALES MG 87.1 µg
SPINAL CORD, LUMBAR; (continued)		
Meninges; INFILTRATION; Mononuclear cell; Multifocal; Minimal	V002591	
Axon; DEGENERATION; Focal; Minimal		V002610
DRG, CERVICAL;		
Examined	(3)	(5)
Within Normal Limits	V002591	V001935<br>V002047<br>V002614
MINERALIZATION; Minimal	V002613	
Ganglion cell; VACUOLATION; Minimal	V002597<br>V002613	V002608<br>V002610
DRG, THORACIC;		
Examined	(3)	(5)
Within Normal Limits		V002047<br>V002610<br>V002614
MINERALIZATION; Minimal	V002613	
Ganglion cell; VACUOLATION; Minimal	V002591<br>V002597<br>V002613	V001935
Axon; DEGENERATION; Multifocal; Minimal		V002608

Pathology - Histo Pathology Observations - Animal Cross Reference
15-RS-288 - 40-Week Toxicity Stud of Recombinant-Methionyl Human Glial Cell Line-Derived Neurotrophic Factor (r-metHuGDNF) via Intermittent Bilateral Intraputamenal Convection-Enhanced Delivery in Rhesus Monkeys with a 12-Week Recovery Period

Removal Reason: Recovery Sacrifice

	---- MALES -----	
	MC<br>0 µg	MG<br>87.1 µg
DRG, LUMBAR;		
Examined	(3)	(5)
Within Normal Limits	V002597<br>V002613	V002047
Ganglion cell; VACUOLATION;		
Minimal	V002591	V001935<br>V002608<br>V002610<br>V002614
TRIGEMINAL GANGLIA;		
Examined	(3)	(4)
Within Normal Limits	V002591<br>V002597<br>V002613	V001935<br>V002047<br>V002608<br>V002610

End Of Print

Table 1-3C. Histopathology Cross Reference Table —Satellite

Pathology - Histo Pathology Observations - Animal Cross Reference
15-RS-288 - 40-Week Toxicity Stud of Recombinant-Methionyl Human Glial Cell Line-Derived Neurotrophic Factor (r-metHuGDNF) via Intermittent Bilateral Intraputamenal Convection-Enhanced Delivery in Rhesus Monkeys with a 12-Week Recovery Period

Removal Reason: Terminal Satellite Sacrifice

	MALES SAT 87.1 µg
BRAIN D;	
Examined	(4)
Within Normal Limits	V001633
CATHETER TRACK (CT)	V002603
CT MINERALIZED MATERIAL; Focal; Minimal	V002603
Meninges; FIBROSIS; Focal; Minimal	V001963<br>V002043
BS/PONS O-LT;	
Examined	(4)
Within Normal Limits	V001633<br>V001963<br>V002043<br>V002603
BS/PONS O-RT;	
Examined	(4)
Within Normal Limits	V001633<br>V001963<br>V002043<br>V002603
CB/MO T-CB;	
Examined	(3)
Within Normal Limits	V001633<br>V002043<br>V002603
CB/MO T-LT;	
Examined	(3)
Within Normal Limits	V001633<br>V002043<br>V002603

Pathology - Histo Pathology Observations - Animal Cross Reference
15-RS-288 - 40-Week Toxicity Stud of Recombinant-Methionyl Human Glial Cell Line-Derived Neurotrophic Factor (r-metHuGDNF) via Intermittent Bilateral Intraputamenal Convection-Enhanced Delivery in Rhesus Monkeys with a 12-Week Recovery Period

Removal Reason: Terminal Satellite Sacrifice

	MALES SAT 87.1 µg
CB/MO T-RT;	
Examined	(3)
Within Normal Limits	V001633<br>V002043<br>V002603
CB/MO U-CB;	
Examined	(1)
Within Normal Limits	V001963
CB/MO U-LT;	
Examined	(1)
Within Normal Limits	V001963
CB/MO U-RT;	
Examined	(1)
Within Normal Limits	V001963
CB R-CB;	
Examined	(3)
Within Normal Limits	V001633<br>V002043<br>V002603
CB S-CB;	
Examined	(1)
Within Normal Limits	V001963
CB V-CB;	
Examined	(4)
Within Normal Limits	V001633<br>V001963<br>V002043<br>V002603
CD F-LT;	
Examined	(1)
CATHETER TRACK (CT)	V001633

Pathology - Histo Pathology Observations - Animal Cross Reference
15-RS-288 - 40-Week Toxicity Stud of Recombinant-Methionyl Human Glial Cell Line-Derived Neurotrophic Factor (r-metHuGDNF) via Intermittent Bilateral Intraputamenal Convection-Enhanced Delivery in Rhesus Monkeys with a 12-Week Recovery Period

Removal Reason: Terminal Satellite Sacrifice

	MALES SAT 87.1 µg
CD F-LT; (continued)	
CT PIGMENTED MACROPHAGES; Focal; Minimal	V001633
CT FIBROSIS; Focal; Minimal	V001633
CT FOREIGN BODY REACTION; Focal; Minimal	V001633
GDNF IMMUNOSTAINING; Moderate	V001633
CD F-RT; Examined	(1)
CATHETER TRACK (CT)	V001633
CT FOREIGN BODY REACTION; Multifocal; Minimal	V001633
CT FIBROSIS; Minimal	V001633
GDNF IMMUNOSTAINING; Moderate	V001633
CD G-LT; Examined	(2)
CATHETER TRACK (CT)	V001963<br>V002043
CT FOREIGN BODY REACTION; Focal; Minimal	V001963
CT FOREIGN BODY REACTION; Multifocal; Minimal	V002043

Pathology - Histo Pathology Observations - Animal Cross Reference
15-RS-288 - 40-Week Toxicity Stud of Recombinant-Methionyl Human Glial Cell Line-Derived Neurotrophic Factor (r-metHuGDNF) via Intermittent Bilateral Intraputamenal Convection-Enhanced Delivery in Rhesus Monkeys with a 12-Week Recovery Period

Removal Reason: Terminal Satellite Sacrifice

	MALES SAT 87.1 µg
CD G-LT; (continued)	
CT INFILTRATION, MONONUCLEAR CELL; Focal; Minimal	V001963
CT INFILTRATION, MONONUCLEAR CELL; Multifocal; Minimal	V002043
CT VACUOLATION, WHITE MATTER; Multifocal; Minimal	V001963
GDNF IMMUNOSTAINING; Moderate	V001963
CD G-RT; Examined	(2)
CATHETER TRACK (CT)	V001963<br>V002043
CT FOREIGN BODY REACTION; Multifocal; Minimal	V002043
CT PIGMENTED MACROPHAGES; Focal; Minimal	V001963
CT GLIOSIS/ASTROCYTOSIS; Minimal	V001963<br>V002043
CT VACUOLATED MACROPHAGES; Multifocal; Minimal	V001963
CT VACUOLATION, WHITE MATTER; Focal; Mild	V001963
CT VACUOLATION, WHITE MATTER; Multifocal; Minimal	V002043

Pathology - Histo Pathology Observations - Animal Cross Reference
15-RS-288 - 40-Week Toxicity Stud of Recombinant-Methionyl Human Glial Cell Line-Derived Neurotrophic Factor (r-metHuGDNF) via Intermittent Bilateral Intraputamenal Convection-Enhanced Delivery in Rhesus Monkeys with a 12-Week Recovery Period

Removal Reason: Terminal Satellite Sacrifice	MALES SAT 87.1 µg
CD G-RT; (continued)	
GDNF IMMUNOSTAINING;	
Moderate	V001963<br>V002043
CD/PUT G-LT;	
Examined	(2)
CATHETER TRACK (CT)	V001633<br>V002603
CT PIGMENTED MACROPHAGES; Focal;	
Minimal	V002603
CT PIGMENTED MACROPHAGES; Multifocal;	
Minimal	V001633
CT INFILTRATION, MONONUCLEAR CELL; Multifocal;	
Minimal	V001633
CT FIBROSIS;	
Minimal	V001633
CT FOREIGN BODY REACTION; Focal;	
Minimal	V002603
CT FOREIGN BODY REACTION; Multifocal;	
Minimal	V001633
CT VACUOLATION, WHITE MATTER; Focal;	
Minimal	V002603
CT HEMORRHAGE; Multifocal;	
Minimal	V001633
CT MINERALIZED MATERIAL;	
Minimal	V002603

Pathology - Histo Pathology Observations - Animal Cross Reference
15-RS-288 - 40-Week Toxicity Stud of Recombinant-Methionyl Human Glial Cell Line-Derived Neurotrophic Factor (r-metHuGDNF) via Intermittent Bilateral Intraputamenal Convection-Enhanced Delivery in Rhesus Monkeys with a 12-Week Recovery Period

Removal Reason: Terminal Satellite Sacrifice

	MALES SAT 87.1 µg
CD/PUT G-LT; (continued)	
GDNF IMMUNOSTAINING;	
Moderate	V001633<br>V002603
CD/PUT G-RT;	
Examined	(2)
CATHETER TRACK (CT)	V001633<br>V002603
CT FOREIGN BODY REACTION; Focal;	
Minimal	V002603
CT FOREIGN BODY REACTION; Multifocal;	
Minimal	V001633
CT INFILTRATION, MONONUCLEAR CELL; Focal;	
Minimal	V001633
CT PIGMENTED MACROPHAGES; Focal;	
Minimal	V002603
CT VACUOLATION, WHITE MATTER; Focal;	
Minimal	V002603
CT INFILTRATION, EOSINOPHIL; Focal;	
Minimal	V001633
CT PERIVASCULAR CUFFS; Mixed; Focal;	
Minimal	V001633
CT MINERALIZED MATERIAL;	
Minimal	V001633<br>V002603
GDNF IMMUNOSTAINING;	
Moderate	V001633

Pathology - Histo Pathology Observations - Animal Cross Reference
15-RS-288 - 40-Week Toxicity Stud of Recombinant-Methionyl Human Glial Cell Line-Derived Neurotrophic Factor (r-metHuGDNF) via Intermittent Bilateral Intraputamenal Convection-Enhanced Delivery in Rhesus Monkeys with a 12-Week Recovery Period

Removal Reason: Terminal Satellite Sacrifice	MALES<br>SAT<br>87.1 µg
CD/PUT H-LT;	
Examined	(4)
CATHETER TRACK (CT)	V001633<br>V001963<br>V002043<br>V002603
CT FIBROSIS;	
Mild	V001633
CT GLIOSIS/ASTROCYTOSIS;	
Minimal	V002043
Mild	V001963
CT INFILTRATION, MONONUCLEAR CELL; Focal;	
Minimal	V002043
CT INFILTRATION, MONONUCLEAR CELL; Multifocal;	
Minimal	V001633
CT PIGMENTED MACROPHAGES; Focal;	
Minimal	V002603
CT PIGMENTED MACROPHAGES; Multifocal;	
Mild	V001963
CT INFILTRATION, EOSINOPHIL; Multifocal;	
Minimal	V001633
CT FOREIGN BODY REACTION; Focal;	
Minimal	V001963<br>V002603
CT FOREIGN BODY REACTION; Multifocal;	
Mild	V002043

Pathology - Histo Pathology Observations - Animal Cross Reference
15-RS-288 - 40-Week Toxicity Stud of Recombinant-Methionyl Human Glial Cell Line-Derived Neurotrophic Factor (r-metHuGDNF) via Intermittent Bilateral Intraputamenal Convection-Enhanced Delivery in Rhesus Monkeys with a 12-Week Recovery Period

Removal Reason: Terminal Satellite Sacrifice	MALES<br>SAT<br>87.1 µg
CD/PUT H-LT; (continued)	
CT VACUOLATION, GRAY MATTER; Focal;	
Minimal	V001963
CT MINERALIZED MATERIAL;	
Minimal	V001633<br>V001963<br>V002603
GDNF IMMUNOSTAINING;	
Moderate	V001633<br>V001963<br>V002043<br>V002603
Meninges; FIBROSIS; Focal;	
Minimal	V002603
CD/PUT H-RT;	
Examined	(4)
CATHETER TRACK (CT)	V001633<br>V001963<br>V002043<br>V002603
CT FIBROSIS;	
Minimal	V001633<br>V001963
Mild	V002043
CT GLIOSIS/ASTROCYTOSIS;	
Minimal	V001963<br>V002603
CT INFILTRATION, MONONUCLEAR CELL; Focal;	
Minimal	V001633<br>V001963

Pathology - Histo Pathology Observations - Animal Cross Reference
15-RS-288 - 40-Week Toxicity Stud of Recombinant-Methionyl Human Glial Cell Line-Derived Neurotrophic Factor (r-metHuGDNF) via Intermittent Bilateral Intraputamenal Convection-Enhanced Delivery in Rhesus Monkeys with a 12-Week Recovery Period

Removal Reason: Terminal Satellite Sacrifice	MALES<br>SAT<br>87.1 µg
CD/PUT H-RT; (continued)	
Minimal (continued)	V002043
CT PIGMENTED MACROPHAGES; Multifocal;	
Minimal	V001633<br>V001963<br>V002603
CT VACUOLATED MACROPHAGES; Multifocal;	
Mild	V001963
CT VACUOLATION, WHITE MATTER; Focal;	
Minimal	V002043<br>V002603
CT VACUOLATION, WHITE MATTER; Multifocal;	
Minimal	V001963
CT FOREIGN BODY REACTION; Focal;	
Minimal	V001963<br>V002603
CT FOREIGN BODY REACTION; Multifocal;	
Minimal	V001633<br>V002043
CT VACUOLATION, GRAY MATTER; Multifocal;	
Minimal	V001963
CT MINERALIZED MATERIAL;	
Minimal	V001633<br>V001963<br>V002043
GDNF IMMUNOSTAINING;	
Moderate	V001633<br>V001963<br>V002043

Pathology - Histo Pathology Observations - Animal Cross Reference
15-RS-288 - 40-Week Toxicity Stud of Recombinant-Methionyl Human Glial Cell Line-Derived Neurotrophic Factor (r-metHuGDNF) via Intermittent Bilateral Intraputamenal Convection-Enhanced Delivery in Rhesus Monkeys with a 12-Week Recovery Period

Removal Reason: Terminal Satellite Sacrifice

	MALES SAT 87.1 µg
CD/PUT I-RT;	
Examined	(4)
CATHETER TRACK (CT)	V001633<br>V001963<br>V002043
CT FOREIGN BODY REACTION; Multifocal; Minimal	V001633<br>V001963<br>V002043
CT PIGMENTED MACROPHAGES; Focal; Minimal	V002043
CT PIGMENTED MACROPHAGES; Multifocal; Minimal	V001633<br>V001963
CT INFILTRATION, MONONUCLEAR CELL; Focal; Minimal	V001633
CT FIBROSIS; Focal; Minimal	V001633<br>V002043
CT FIBROSIS; Multifocal; Minimal	V001963
CT GLIOSIS/ASTROCYTOSIS; Multifocal; Minimal	V001963
CT VACUOLATION, GRAY MATTER; Multifocal; Minimal	V001963
CT MINERALIZED MATERIAL; Minimal	V001963

Pathology - Histo Pathology Observations - Animal Cross Reference
15-RS-288 - 40-Week Toxicity Stud of Recombinant-Methionyl Human Glial Cell Line-Derived Neurotrophic Factor (r-metHuGDNF) via Intermittent Bilateral Intraputamenal Convection-Enhanced Delivery in Rhesus Monkeys with a 12-Week Recovery Period

Removal Reason: Terminal Satellite Sacrifice	MALES SAT 87.1 µg
CD/PUT I-RT; (continued)	
GDNF IMMUNOSTAINING;	
Mild	V002603
Moderate	V001963
Marked	V002043
CD/PUT/AC I-LT;	
Examined	(4)
CATHETER TRACK (CT)	V001633<br>V001963<br>V002043<br>V002603
CT FIBROSIS;	
Minimal	V001633<br>V002043
CT PIGMENTED MACROPHAGES; Focal;	
Minimal	V001633
CT PIGMENTED MACROPHAGES; Multifocal;	
Minimal	V001963<br>V002043
CT FOREIGN BODY REACTION; Focal;	
Minimal	V001633
CT FOREIGN BODY REACTION; Multifocal;	
Minimal	V001963
CT VACUOLATION, WHITE MATTER; Focal;	
Minimal	V001963<br>V002043

Pathology - Histo Pathology Observations - Animal Cross Reference
15-RS-288 - 40-Week Toxicity Stud of Recombinant-Methionyl Human Glial Cell Line-Derived Neurotrophic Factor (r-metHuGDNF) via Intermittent Bilateral Intraputamenal Convection-Enhanced Delivery in Rhesus Monkeys with a 12-Week Recovery Period

Removal Reason: Terminal Satellite Sacrifice

	MALES SAT 87.1 µg
CD/PUT/AC I-LT; (continued)	
CT INFILTRATION, MONONUCLEAR CELL; Focal;	
Minimal	V001633
CT INFILTRATION, MONONUCLEAR CELL; Multifocal;	
Minimal	V001963<br>V002043
CT GLIOSIS/ASTROCYTOSIS;	
Minimal	V001963
CT MINERALIZED MATERIAL;	
Minimal	V001963
GDNF IMMUNOSTAINING;	
Moderate	V001633<br>V001963<br>V002043<br>V002603
CD/PUT/GP J-LT;	
Examined	(4)
Within Normal Limits	V001963
CATHETER TRACK (CT)	V002043<br>V002603
CT FOREIGN BODY REACTION; Focal;	
Minimal	V002603
CT FOREIGN BODY REACTION; Multifocal;	
Minimal	V002043
CT PIGMENTED MACROPHAGES; Multifocal;	
Minimal	V002603
CT FIBROSIS; Focal;	
Minimal	V002603

Pathology - Histo Pathology Observations - Animal Cross Reference
15-RS-288 - 40-Week Toxicity Stud of Recombinant-Methionyl Human Glial Cell Line-Derived Neurotrophic Factor (r-metHuGDNF) via Intermittent Bilateral Intraputamenal Convection-Enhanced Delivery in Rhesus Monkeys with a 12-Week Recovery Period

Removal Reason: Terminal Satellite Sacrifice

	MALES SAT 87.1 µg
CD/PUT/GP J-LT; (continued)	
CT GLIOSIS/ASTROCYTOSIS; Focal;	
Minimal	V002043
CT MINERALIZED MATERIAL;	
Minimal	V002043<br>V002603
AXON SPHEROIDS; Focal;	
Mild	V001633
GDNF IMMUNOSTAINING;	
Moderate	V002043<br>V002603
CD/PUT/GP J-RT;	
Examined	(4)
Within Normal Limits	V001633<br>V001963
CATHETER TRACK (CT)	V002043<br>V002603
CT GLIOSIS/ASTROCYTOSIS;	
Minimal	V002043
CT FIBROSIS;	
Minimal	V002603
CT FOREIGN BODY REACTION; Focal;	
Minimal	V002603
CT FOREIGN BODY REACTION; Multifocal;	
Mild	V002043
CT PIGMENTED MACROPHAGES; Focal;	
Minimal	V002603

Pathology - Histo Pathology Observations - Animal Cross Reference
15-RS-288 - 40-Week Toxicity Stud of Recombinant-Methionyl Human Glial Cell Line-Derived Neurotrophic Factor (r-metHuGDNF) via Intermittent Bilateral Intraputamenal Convection-Enhanced Delivery in Rhesus Monkeys with a 12-Week Recovery Period

Removal Reason: Terminal Satellite Sacrifice

	MALES SAT 87.1 µg
CD/PUT/GP J-RT; (continued)	
CT MINERALIZED MATERIAL;	
Minimal	V002043<br>V002603
GDNF IMMUNOSTAINING;	
Moderate	V002603
PUT/GP K-LT;	
Examined	(3)
Within Normal Limits	V001963<br>V002043
GDNF IMMUNOSTAINING;	
Mild	V002603
PUT/GP K-RT;	
Examined	(3)
Within Normal Limits	V001963<br>V002043
CATHETER TRACK (CT)	V002603
CT FIBROSIS; Focal;	
Minimal	V002603
CT PIGMENTED MACROPHAGES; Focal;	
Minimal	V002603
GDNF IMMUNOSTAINING;	
Moderate	V002603
SN M;	
Examined	(1)
Within Normal Limits	V001633
SN N;	
Examined	(3)
Within Normal Limits	V002043

Pathology - Histo Pathology Observations - Animal Cross Reference
15-RS-288 - 40-Week Toxicity Stud of Recombinant-Methionyl Human Glial Cell Line-Derived Neurotrophic Factor (r-metHuGDNF) via Intermittent Bilateral Intraputamenal Convection-Enhanced Delivery in Rhesus Monkeys with a 12-Week Recovery Period

Removal Reason: Terminal Satellite Sacrifice

	MALES<br>SAT<br>87.1 µg
SN N; (continued)	
Within Normal Limits (continued)	V002603
Meninges; INFILTRATION; Mononuclear cell; Focal; Minimal	V001963
THAL/SN L;	
Examined	(1)
AXON SPHEROIDS; Focal; Minimal	V001633
Perivascular; INFILTRATION; Mononuclear cell; Focal; Minimal	V001633
THAL/SN M;	
Examined	(2)
Within Normal Limits	V002043<br>V002603
THAL/SN M-LT;	
Examined	(1)
Within Normal Limits	V001963
THAL/SN M-RT;	
Examined	(1)
Within Normal Limits	V001963
THAL/STN K-LT;	
Examined	(1)
AXON SPHEROIDS; Focal; Minimal	V001633
THAL/STN K-RT;	
Examined	(1)
Within Normal Limits	V001633

Pathology - Histo Pathology Observations - Animal Cross Reference
15-RS-288 - 40-Week Toxicity Stud of Recombinant-Methionyl Human Glial Cell Line-Derived Neurotrophic Factor (r-metHuGDNF) via Intermittent Bilateral Intraputamenal Convection-Enhanced Delivery in Rhesus Monkeys with a 12-Week Recovery Period

Removal Reason: Terminal Satellite Sacrifice

	MALES SAT 87.1 µg
**THAL/STN L;**	
Examined	(1)
Within Normal Limits	V002603
**THAL/STN L-LT;**	
Examined	(2)
Within Normal Limits	V001963<br>V002043
**THAL/STN L-RT;**	
Examined	(2)
Within Normal Limits	V001963<br>V002043
**SPINAL CORD, CERVICAL;**	
Examined	(4)
Within Normal Limits	V001633<br>V001963<br>V002043<br>V002603
**SPINAL CORD, THORACIC;**	
Examined	(4)
Within Normal Limits	V001633<br>V001963<br>V002043<br>V002603
**SPINAL CORD, LUMBAR;**	
Examined	(4)
Within Normal Limits	V001633<br>V001963<br>V002043<br>V002603
**DRG, CERVICAL;**	
Examined	(4)
Within Normal Limits	V001633<br>V001963

Pathology - Histo Pathology Observations - Animal Cross Reference
15-RS-288 - 40-Week Toxicity Stud of Recombinant-Methionyl Human Glial Cell Line-Derived Neurotrophic Factor (r-metHuGDNF) via Intermittent Bilateral Intraputamenal Convection-Enhanced Delivery in Rhesus Monkeys with a 12-Week Recovery Period

Removal Reason: Terminal Satellite Sacrifice

	MALES SAT 87.1 µg
DRG, CERVICAL; (continued)	
Within Normal Limits (continued)	V002043<br>V002603
DRG, THORACIC;	
Examined	(4)
Within Normal Limits	V001633<br>V001963<br>V002043<br>V002603
DRG, LUMBAR;	
Examined	(4)
Within Normal Limits	V001633<br>V001963<br>V002043<br>V002603
TRIGEMINAL GANGLIA;	
Examined	(4)
Within Normal Limits	V001633<br>V001963<br>V002043<br>V002603

End Of Print

APPENDIX 2: GLOSSARY OF MORPHOLOGIC TERMS AND EXPLANATION OF SEVERITY GRADES

Catheter Track

Catheter Track (CT): This entry was made (and recorded only as "Present", not graded) if a hole where the catheter passed was visible or, in some sections, a characteristic reaction associated with the catheter track was observed although the lumen of the hole was not visible in the section.

CT Axonal Spheroids: Denotes swollen axonal profiles with eosinophilic, granular staining in the neuropil adjacent to the catheter track.

CT Fibrosis: Denotes fibrous connective tissue surrounding the catheter track.

CT Foreign Body Reaction: Denotes multinucleated giant cells lining the catheter track.

CT Gliosis/Astrocytosis: Denotes an increase is glial cells (astrocytes or microglia) adjacent to the catheter track.

CT Hemorrhage: Denotes the presence of red blood cells outside the vascular space adjacent to the catheter track.

CT Infiltration (Mononuclear cell, Neutrophil, or Eosinophil): Denotes the presence of inflammatory cells adjacent to the catheter track.

CT Mineralized Material: Denotes the presence of mineralized material in the catheter track. This material was interpreted as representing dystrophic mineralization of debris surrounding the catheter. Debris was likely created by the physical disruption of the neurophil due to catheterization.

CT Perivascular Cuffs (Mononuclear cell or mixed cell): Denotes the presence of inflammatory cells surrounding blood vessels in the parenchyma in the area surrounding the catheter track.

CT Pigmented Macrophages: Denotes the presence of macrophages containing brown or gray-brown pigment adjacent to the catheter track. Most often, the pigment was brown and was interpreted as hemosiderin indicating previous hemorrhage at the catheter track.

CT Vacuolated Macrophages: Denotes the presence of vacuolated macrophages in the vicinity of the catheter track. The vacuolated appearance of the macrophages suggests the phagocytosis of a lipid rich material, most likely myelin, from the degeneration of axons in the area of the catheter track. However, in some instances, the macrophages had both a vacuolated and a granular

appearance. The granular material was eosinophilic and was interpreted as phagocytosis of cellular debris due to cell degeneration in the neuropil.

CT Vacuolation, White Matter or Gray Matter: Denotes the presence of a separation of parenchyma (white or gray as indicated) in the vicinity of the catheter track. This separation of the parenchyma could have been due to fluid flowing out of the catheter, fluid (fixative) leaking from the vasculature during intravascular perfusion at necropsy, or edema (fluid leaking from the vascular system in life).

Non Catheter Track

Axonal Degeneration (brain or spinal cord): Denotes the presence of dilated myelin sheaths, axonal debris, and/or macrophages within myelin sheaths, which are features of axonal degeneration.

Axonal Spheroids: Denotes swollen axonal profiles with granular, eosinophilic staining in the neuropil in a section that did not contain a catheter track.

Fibrosis, Meninges: Denotes foci with an increase in collagen in the meninges.

Ganglion Cells, Satellitosis: An increase of satellite cells surrounding sensory ganglia neurons. This finding is commonly observed in ganglia and is considered a sporadic change of no significance or within normal variation.

Ganglion Cells, Vacuolation: Denotes the presence of large vacuoles in ganglion cells in the trigeminal ganglion or dorsal root ganglia. These vacuoles are commonly observed in ganglion cells and are considered a sporadic change of no significance.

Infiltrate, Mononuclear Cell or mixed, Meninges or Choroid Plexus: Denotes the presence of mononuclear or mixed cell infiltrates in the meninges or choroid plexus.

Perivascular Cuffs (Mononuclear or Mixed): Denotes the presence of mononuclear or mixed inflammatory cells surrounding blood vessels in the parenchyma away from the catheter track or in a section that did not contain a catheter track.

Vacuolation, White Matter: Denotes the presence of a separation of parenchyma (white or gray as indicated) in an area away from a catheter track or in a section that did not contain a catheter track. This separation of the parenchyma could have been due to fluid (fixative) leaking from the vasculature during intravascular perfusion at necropsy or edema (fluid leaking from the vascular system in life). Because of the lack of other evidence of vascular damage, it was interpreted as most likely representing an artefact of vascular perfusion.

GDNF Immunostaining

GDNF Immunostaining: Denotes the presence of positive immunostaining for GDNF in the immunohistiochemically stained slide. In sections that contained a catheter track, the GDNF immunostaining was in the catheter track and/or neuropil adjacent to the catheter track. The staining was graded as minimal if immunostaining was confined to the catheter track or the fibrous capsule of the catheter track. The immunostaining was graded as mild if it was present in the catheter track or capsule and extended into any of the neuropil adjacent to the catheter track. Grades of moderate or marked indicated a more extensive immunostaining in the neuropil. If GDNF immunostaining was observed away from the catheter track or in a section that did not contain a catheter track, a grade was entered to indicate the relative amount of immunostaining and a comment was added in Provantis to note the presence of staining away from the catheter track.

Grading of Findings

Severity Grades for Histopathology Findings: For findings in the catheter track or adjacent to the catheter track, the grading scale reflects the extent and/or severity of the finding in the area around the catheter track and therefore relates to a local severity.

APPENDIX 3: DEVIATION REPORT FOR MISSING TISSUES

SEVENTH WAVE DEVIATION REPORT

Study Title: 40-Week Toxicity Study of Recombinant-Methionyl Human Glial Cell Line-Derived Neurotrophic Factor (r-metHuGDNF) via Intermittent Bilateral Intraputamenal Convection-Enhanced Delivery in Rhesus Monkeys with a 12-Week Recovery Period

Seventh Wave Number: 15-RS-288
Valley Biosystems Study Number: S14-10463
MedGenesis Therapeutix, Inc Number: MGT03-PRE003

Type of Deviation: Protocol

Nature of Incident:
The tissue as listed below was not examined.

Animal Number	Missing Tissue	Pathologist Comments
V002614	Trigeminal Ganglia	Insufficient tissue to evaluate following recut or reharvest

Corrective Action:
The following is a description of actions taken for specific non-examination reasons:

- For tissues that were insufficient to evaluate, a recut or reharvest (as appropriate) was performed.

Impact of Deviation:
The tissue that was suboptimal (insufficient) or missing was not target tissue and was present in a sufficient number of animals to allow the pathologist to evaluate the test article effect within the dose group. Therefore, this deviation from the protocol has no adverse impact on the pathologist's ability to evaluate test article effect.
